# Genome-wide identification, stress- and hormone-responsive expression characteristics, and regulatory pattern analysis of *Scutellaria baicalensis SbSPLs*

**DOI:** 10.1007/s11103-023-01410-z

**Published:** 2024-02-16

**Authors:** Jia-wen Wu, Zi-yi Zhao, Ren-chuan Hu, Yun-feng Huang

**Affiliations:** 1 College of Horticulture and Landscape Architecture, Northeast Agricultural University, Harbin, 150000 China; 2 Guangxi Key Laboratory of Traditional Chinese Medicine Quality Standards, Guangxi Institute of Chinese Medicine and Pharmaceutical Science, Nanning, 530022 China

**Keywords:** *Scutellaria baicalensis* SPL transcription factor, Expression pattern, Genetic and evolutionary information

## Abstract

SQUAMOSA PROMOTER BINDING PROTEIN-LIKEs (*SPLs*) encode plant-specific transcription factors that regulate plant growth and development, stress response, and metabolite accumulation. However, there is limited information on *Scutellaria baicalensis SPLs*. In this study, 14 *SbSPLs* were identified and divided into 8 groups based on phylogenetic relationships. *SbSPLs* in the same group had similar structures. Abscisic acid-responsive (ABRE) and MYB binding site (MBS) cis-acting elements were found in the promoters of 8 and 6 *SbSPLs*. Segmental duplications and transposable duplications were the main causes of *SbSPL* expansion. Expression analysis based on transcriptional profiling showed that *SbSPL1*, *SbSPL10*, and *SbSPL13* were highly expressed in roots, stems, and flowers, respectively. Expression analysis based on quantitative real-time polymerase chain reaction (RT‒qPCR) showed that most *SbSPLs* responded to low temperature, drought, abscisic acid (ABA) and salicylic acid (SA), among which the expression levels of *SbSPL7/9/10/12* were significantly upregulated in response to abiotic stress. These results indicate that *SbSPLs* are involved in the growth, development and stress response of *S. baicalensis*. In addition, 8 Sba-miR156/157 s were identified, and *SbSPL1-5* was a potential target of Sba-miR156/157 s. The results of target gene prediction and coexpression analysis together indicated that *SbSPLs* may be involved in the regulation of L-phenylalanine (L-Phe), lignin and jasmonic acid (JA) biosynthesis. In summary, the identification and characterization of the *SbSPL* gene family lays the foundation for functional research and provides a reference for improved breeding of *S. baicalensis* stress resistance and quality traits.

**Supplementary Information:**

The online version contains supplementary material available at 10.1007/s11103-023-01410-z.

## Key message

This report details the characteristics and potential regulatory network of the *S. baicalensis SbSPL* gene family and demonstrates the potential role of *SbSPLs* in the abiotic stress response and material accumulation.

**Supplementary Information:**

The online version contains supplementary material available at 10.1007/s11103-023-01410-z.

## Introduction

*Scutellaria baicalensis* is called Huang-Qin in Chinese, which means golden (precious) herb. It belongs to the perennial herb family Lamiaceae and is commonly used in traditional medicine. *S. baicalensis* is distributed in East Asia, Europe and North America, and it is used for adjuvant therapy of various diseases (Wen et al. 2022; Zhao et al. 2016). It has been officially listed in the Chinese Pharmacopoeia (2020), European Pharmacopoeia (EP 9.0) and British Pharmacopoeia (BP 2018). The root is the main medicinal part of *S. baicalensis* (Zhao et al. 2016), and flavonoids and their glycosides are the main medicinal components that have important antiviral (Zhao et al. 2019b), antidiabetic (Huang et al. 2017; Na and Lee 2019), and anticancer (Park et al. 2021; Xiang et al. 2022) functions. It is worth noting that the secondary metabolites of *S. baicalensis* have an important effect when used to treat coronavirus disease 2019 (COVID-19) (Malekmohammad and Rafieian-Kopaei 2021; Song et al. 2020). Liu et al. and Su et al. found that baicalein and wogonin in *S. baicalensis* extract show strong anti-COVID-19 activity in cell systems (Liu et al. 2021; Su et al. 2020). Therefore, research on *S. baicalensis* is of great medical importance.

Abiotic stress is a key factor affecting the yield and quality of *S. baicalensis* under natural conditions. First, abiotic stress produces reactive oxygen species (ROS) to damage the tissue cells of *S. baicalensis*. For example, Zhang et al. found that short-term drought led to an imbalance in ROS metabolism, and *S. baicalensis* regulated ROS levels by increasing the activities of protective enzymes such as superoxide dismutase (SOD) and peroxidase (POD) (Zhang et al. 2013). Second, the accumulation of secondary metabolites in *S. baicalensis* is associated with abiotic stress. Ultraviolet-B (UV-B) radiation can significantly affect the accumulation of secondary metabolite aglycones (including baicalein, wogonin and scutellarein) in tissue culture and produce oxidative stress (Yun et al. 2022). Yuan et al. found that abnormal temperature (10 °C and 40 °C) reduced the content of flavonoids in *S. baicalensis*, and high temperature affected the synthesis of two key enzymes, 7-O-glucuronosyltransferase and β-glucuronidase, in the interconversion of baicalin and baicalein (Yuan et al. 2012). Guo et al. also found that the content of secondary metabolites was positively correlated with temperature changes within a certain range (Guo et al. 2013). In addition, heavy metals such as cadmium, mercury and plumbum were also unfavorable factors for the growth and accumulation of medicinal components in *S. baicalensis* (Meng et al. 2022).

The transcription initiation process of eukaryotes is very complex and often requires the assistance of a variety of protein factors. Transcription factors (TFs) form a complex with RNA polymerase and participate in the transcription initiation process together. TFs in plants generally exist in the form of gene families and play an important role in plant signal transduction (Wani et al. 2021). SQUAMOSA-promoter binding protein-like (SPL) proteins are a family of plant-specific TFs found in many green plants. The first discovery of SPL TFs was in 1996, when Peter Huijser et al. cloned two genes in the *Antirrhinum majus* flower meristem. The genes both contain a conserved squamosa-promoter binding protein (SBP)-box domain and can bind to the promoter of the floral meristem characteristic gene *SQUAMOSA* (*SQUA*). The genes were later named *AmSBP1* and *AmSBP2* (Klein et al. 1996). Birkenbihl et al. found that the SBP-box protein is a highly conserved domain consisting of 76 amino acid residues and includes two finger-like zinc finger structures formed by the coordination of Zn^2+^, namely, Cys-Cys-Cys-His and Cys-Cys-His-Cys, and a nuclear localization signal (NLS) at the C-terminus of the SBP domain that partially overlaps the second zinc finger (Birkenbihl et al. 2005). In previous studies, members of the *Arabidopsis thaliana* AtSPL TFs were divided into 8 groups (I–VIII) (Cardon et al. 1999). With the development of plant whole-genome sequencing and bioinformatics, SPL TFs have been identified and analyzed in many species, including the dicotyledons *Ziziphus jujuba* (Shao et al. 2017), *Fragaria vesca* (Xiong et al. 2018), and *Solanum lycopersicum* (Salinas et al. 2012), and monocotyledons *Oryza sativa* (Xie et al. 2006), *Triticum aestivum* (Zhu et al. 2020), and *Senna italica* (Lai et al. 2022). An increasing number of studies have shown that *SPL* genes can actively respond to abiotic stresses such as drought, salt, and abnormal temperature. Overexpression of *Vitis pseudoreticulata VpSBP16* could enhance the scavenging ability of ROS to enhance the salt tolerance and drought tolerance of grapes (Hou et al. 2018). In addition, 3 differentially expressed *MsSPLs* (*MsSPL17*, *MsSPL23* and *MsSPL36*) were found in *Medicago sativa* under salt stress, which provided comprehensive information for the mechanism underlying salt tolerance improvement in *M. sativa* (He et al. 2022b).

MiR156/157 s are small noncoding RNAs (approximately 20 nt in length) produced by nuclease cutting and processing long-chain pri-miRNAs and are highly conserved in plants (Lopez-Ortiz et al. 2021). The sequences of miR156s and miR157s are highly similar, with only 1/2 different nucleotides (Zhou et al. 2021). Posttranscriptional mRNAs of *SPLs* with miRNA response elements (MREs) complementary to miR156 were cleaved and/or translationally repressed by miR156 (Rhoades et al. 2002). The key role of the miR156/157-SPL regulatory module in plant responses to abiotic stress has become increasingly clear. Eight of the 17 *DgSPLs* of *Dactylis glomerata* may be potential targets of miR156, and 2 of these 8 genes (*DG1G01828.1* and *DG0G01071.1*) can simultaneously respond to drought, salt and heat stress (Feng et al. 2021). The tolerance of *M. sativa* to heat stress (40 °C) was increased after overexpression of miR156 and knockdown of *MsSPL13* RNAi, suggesting that the miR156/SPL13 pathway contributes to the improvement of heat tolerance in *M. sativa* (Matthews et al. 2019). When *A. thaliana* was subjected to repeated heat stress, *AtSPLs* were posttranscriptionally downregulated by miR156, indicating that the miR156-SPL module mediated the *A. thaliana* response to repeated heat stress (Stief et al. 2014). In addition, SPL TFs can regulate plant physiological and metabolic processes to adapt to element-deficient environments. For example, microRNAs (Cu-miRNAs) can reduce the consumption of Cu by plants during periods of copper deficiency. Perea-García et al. found that Cu-deficiency responses mediated by *AtSPL7* binding to Cu-miRNA promoters were often competitively inhibited by the miR156-SPL3 module in *A. thaliana* (Perea-García et al. 2021). In conclusion, previous studies have revealed the responsive function of miR156s-SPLs in a wide range of plant adaptive responses to the environment.

Although TFs have been widely studied in a large number of plants, the exploration of TFs in *S. baicalensis*, a precious Chinese herbal medicine, is rare. We searched Chinese and international journals and found only studies on WRKY TFs (Zhang et al. 2022a) and R2R3-MYB TFs in *S. baicalensis* (Wang et al. 2022). However, research on the *SPL* genes of *S. baicalensis* is completely lacking, and the upstream and downstream regulatory mechanisms of *SbSPLs* are also unknown. To this end, we screened *SbSPLs* and predicted and analyzed their gene structure, motifs, evolution, sequence conservation, cis-acting elements, expression patterns, and upstream and downstream regulatory relationships. The results of this study provide comprehensive information for understanding *SbSPLs* and provide clues for the interpretation of the *S. baicalensis* stress response and the regulation of metabolite biosynthesis.

## Materials and methods

### Identification and chromosomal localization of *SbSPLs*

The genome, protein and annotation files of *S. baicalensis* were downloaded from the National Genomics Data Center (https://ngdc.cncb.ac.cn/search/?dbId=gwh&q=PRJCA009554&page=1, Accessed on September 20, 2022) (Hu et al. 2022). Hidden Markov Model (HMM) seed files for SBP domains were downloaded from the Pfam database (http://pfam.sanger.ac.uk/, Accessed on 20 September 2022) (Punta et al. 2012). Hmmsearch was used to identify the SPL proteins of *S. baicalensis*, and the E-value threshold was 10^–20^ (Potter et al. 2018). Proteins with SBP domains were obtained through Pfam and SMART (http://smart.embl.de/, Accessed on September 20, 2022) database screening (Letunic and Bork 2018). The online ExPASy Bioinformatics Resource Portal (https://web.ExPASy.org/compute_pi/, Accessed on September 20, 2022) was used to predict the molecular weight (MW) and physicochemical properties of proteins (Duvaud et al. 2021). ProtComp 9.0 (http://linux1.softberry.com/berry.phtml?topic=protcomppl&group=programs&subgroup=proloc, Accessed on September 20, 2022) was used for subcellular localization prediction (Emanuelsson et al. 2000). TBtools was used to draw the chromosomal location map of *SbSPLs* based on the annotation files and genome files (Chen et al. 2020).

### Sequence alignment and phylogenetic analysis

We aligned SbSPL and *A. thaliana* AtSPL protein sequences (UniProt database, https://www.UniProt.org) using MAFFT (Consortium 2017; Nakamura et al. 2018). The results are presented by group using Texmaker (Beitz 2000). Subsequently, a phylogenetic tree was constructed based on the alignment results using the maximum likelihood (ML) method in fasttree with a maximum of 1000 bootstrap replicates (Price et al. 2009). The R package ggtree was used for visualization (Yu et al. 2018).

### Gene structure and conserved motif analysis

The location and length of the *SbSPL* CDS were clarified according to the annotation file. MEME Suite version 5.0.5 (http://meme.nbcr.net/meme/) was used to analyze conserved motifs in proteins (Bailey et al. 2009). The parameters were set as follows: maximum number of motifs = 10; optimum width of motifs = 5 to 50. The R package gggenes was used for the visualization of gene structure and conserved motifs (Gómez-Rubio 2017).

### Prediction of cis-acting elements

Bedtools was used to extract the 2.0 kb promoter sequence upstream of *SbSPLs* (Quinlan 2014). Cis-acting elements were analyzed using the PLACE database (https://bioinformatics.psb.ugent.be/webtools/plantcare/html/, Accessed on September 20, 2022) (Higo et al. 1998). TBtools was used to generate heatmaps, and the R package gggenes was used to map the distribution of cis-acting elements (Chen et al. 2020; Gómez-Rubio 2017).

### Gene duplication and evolutionary analysis

MCScanX-transposed was used to analyze the duplication type and calculate the synonymous substitution rate (Ks) and nonsynonymous substitution rate (Ka) (Wang et al. 2013). Subsequently, the differentiation time of the duplicated genes was further calculated according to T = Ks/2λ × 10^–6^ million years ago (Mya). λ is the substitution rate of 6.5 × 10^–9^ bases per synonymous substitution site per year (Li et al. 2023; Zhao et al. 2019a). Circos V0.69 was used to visualize gene duplication relationships (Krzywinski et al. 2009). MCScanX (python) was used to analyze the synteny of *S. baicalensis* with *A. thaliana*, *Salvia miltiorrhiza*, *S. lycopersicum*, *O. sativa*, *Zea mays* and *Glycine max* and for visualization (Wang et al. 2012).

### RNA-seq analysis

Raw RNA-seq data for roots, stems, leaves, and flowers were downloaded from the European Nucleotide Archive (https://www.ebi.ac.uk/ena/browser/view/PRJNA263255) (Hu et al. 2022). Sequences were requalified using fastp to filter out low-quality and linker sequences (Chen et al. 2018). The clean reads (75.26–81.99% of the total reads) were matched to the *S. baicalensis* CDSs using salmon, and the gene expression levels in the form of TPM (transcripts per kilobase of exon model per million mapped reads) were obtained (Patro et al. 2017). Subsequently, expression levels were displayed in the form of a heatmap by TBtools (Chen et al. 2020). Since this dataset had no biological replicates, for a more robust analysis of regulatory pathways, we downloaded the raw RNA-seq data of *S. baicalensis* roots, stems, and leaves published in earlier studies (https://www.ebi.ac.uk/ena/browser/text-search?query=PRJNA515574) (Gao et al. 2019). The TPM gene expression levels were obtained in the same way. The clean reads matched to CDSs accounted for 66.48–84.70% of the total reads.

### Plants and treatments

*S. baicalensis* seeds of the same size with full grains were selected from Yangcheng County, Shanxi Province, China. The seeds were removed and placed in sterile water for imbibition for 12 h. Plants were grown under a 12 h photoperiod, day/night temperatures of 25/18 °C, an irradiance of 50 µmol m^−2^·s^−1^, and air humidity of 50%. Subsequent experiments were carried out after the seedlings had grown for 45 days.

*S. baicalensis* seedlings were used in abiotic stress and hormone treatment experiments according to Wang et al. (Wang et al. 2021). Details are as follows: (1) roots soaked in 20% (m/v) polyethylene glycol 6000 solution (PEG) to simulate drought; (2) 4 °C low temperature treatment; (3) roots soaked in 200 μM SA; (4) roots soaked in 100 μM ABA. Each treatment was replicated 3 times, with 10 seedlings per replicate. At 0, 3, 6, 12 and 24 h after treatment (HAT), 3 whole seedlings from the same replicate were randomly selected and mixed into one sample.

### RT‒qPCR analysis of *SbSPLs*

The FastPure Universal Plant Total RNA Isolation Kit (Vazyme, Nanjing, China) was used to extract total RNA from entire *S. baicalensis* seedlings. Hifair® II 1st Strand cDNA Synthesis SuperMix for qPCR (gDNA digester plus) (Yeasen Biotechnology, Shanghai, China) was used for reverse transcription of the extracted RNA into cDNA. Hieff UNICON® qPCR SYBR Green Master Mix (Yeasen Biotechnology, Shanghai, China) was used for quantitative real-time polymerase chain reaction (RT‒qPCR). The *S. baicalensis* β-actin gene (GenBank: HQ847728) was used as the internal reference gene (Lu et al. 2022). Gene-specific primers for RT‒qPCR were independently designed based on nucleotide polymorphisms in the cDNA sequences of *SbSPL1*—*SbSPL14* (Table S1). The RT‒qPCR procedure was as follows: predenaturation at 95 °C for 30 s; 40 cycles of 95 °C for 5 s and 60 °C for 20 s; and detection of the signal at 72 °C. The 2^−ΔΔCT^ method was used to calculate the expression levels. Three technical replicates were performed for each sample. Fisher’s least significant difference (LSD) method was used for difference analysis in Origin 2023 (Zhang et al. 2022b). The R package ggplot2 was used to draw histograms and annotate the significance analysis results (Gómez-Rubio 2017). The R package CluserGVis was used to conduct time series clustering analysis on the expression of *SbSPLs* under different treatments (Ni et al. 2020). By manually adjusting the number of clusters and observing the membership value, optimal clustering was achieved.

### Prediction of *S. baicalensis miR156/157* genes and their target *SPL* genes

To identify the *miR156/157* genes in *S. baicalensis*, we downloaded the miR156/157 precursor sequences of *A. thaliana* from the miRBase database (Kozomara et al. 2019) and performed a homologous sequence search in the *S. baicalensis* genomic sequence using BLAST + (Shah et al. 2019). After manually removing redundant sequences, the secondary structure of the obtained sequences was predicted using MFold (http://www.mfold.org/, Accessed on 15 October 2022) (Zuker 2003). TBtools was used to map the chromosome location (Chen et al. 2020). In addition, we used the TAPIR tool (http://bioinformatics.psb.ugent.be/webtools/tapir/) to analyze *SbSPLs* potentially targeted by Sba-miR156/157 (Bonnet et al. 2010). The targeting relationship was displayed in the form of a heatmap by TBtools (Chen et al. 2020). Targeted *SbSPLs* were aligned with the reverse-complement sequences of Sba-miR156/157 using MAFFT (Nakamura et al. 2018). The miR156/157 sequences of *A. thaliana* and *S. baicalensis* were aligned in the same way. Alignment results were visualized using Texmaker (Beitz 2000).

### Transcriptional regulation prediction and functional annotation

The 1.5 kb upstream sequence of the transcription start site of all genes was extracted, and the fimo tool of MEME Suite version 5.0.5 was used to predict the target genes that could bind to the conserved motifs of SbSPL proteins (Bailey et al. 2009). The coexpression coefficients of the *SbSPLs* and their possible target genes were calculated according to the expression levels. A correlation coefficient ≥ 0.5 (p value ≤ 0.05) represents a close positive correlation, and a correlation coefficient ≤ − 0.5 (p value ≤ 0.05) represents a close negative correlation. The CDS of *S. baicalensis* was uploaded to the Kyoto Encyclopedia of Genes and Genomes database (https://www.genome.jp/kegg/, Accessed on October 15, 2022) (Ogata et al. 1999), and *A. thaliana* was used as the reference species to obtain the functional annotation information of potential target genes. Cytoscape (v3.7.1) was used to visualize metabolic regulatory networks (Otasek et al. 2019).

## Results

### Chromosome distribution and basic protein information

We identified 14 *SbSPLs* and named them (SbSPL1—SbSPL14) according to chromosome positions (Figure S1). Except for Chr 3 and 5, *SbSPLs* were unevenly distributed on the other 7 chromosomes. *SbSPLs* were most distributed on Chr 2 and 8, with 3 each. Second, there are 2 *SbSPLs* on Chr 1, 7 and 9 and only 1 *SbSPL* on Chr 4 and 6. *SbSPLs* on Chr 1, 6, 7 and 8 were clearly localized to the distal telomeric regions of chromosomes.

The characteristics of SbSPL TFs are shown in Table S2. The protein sequence length varied greatly, ranging from 131 (SbSPL14) to 1076 (SbSPL8) amino acids (aa), and the MW ranged between 14.97 and 118.85 kDa. Most SbSPLs (SbSPL1/2/3/4/6/8/9/11/12/14) are basic (isoelectric point (Ip) > 7). The instability index (II) values were all greater than 40, and the grand average of hydropathicity (GRAVY) scores were all negative, indicating the instability and hydrophilicity of SbSPLs. Furthermore, proteins had different action sites. Subcellular localization predictions suggested that SbSPL2/3/5 were located outside the cell, while others were located in the nucleus (Table S2).

### Phylogenetic and multiple sequence alignment analysis of the SbSPL TFs

**Fig. 1:**
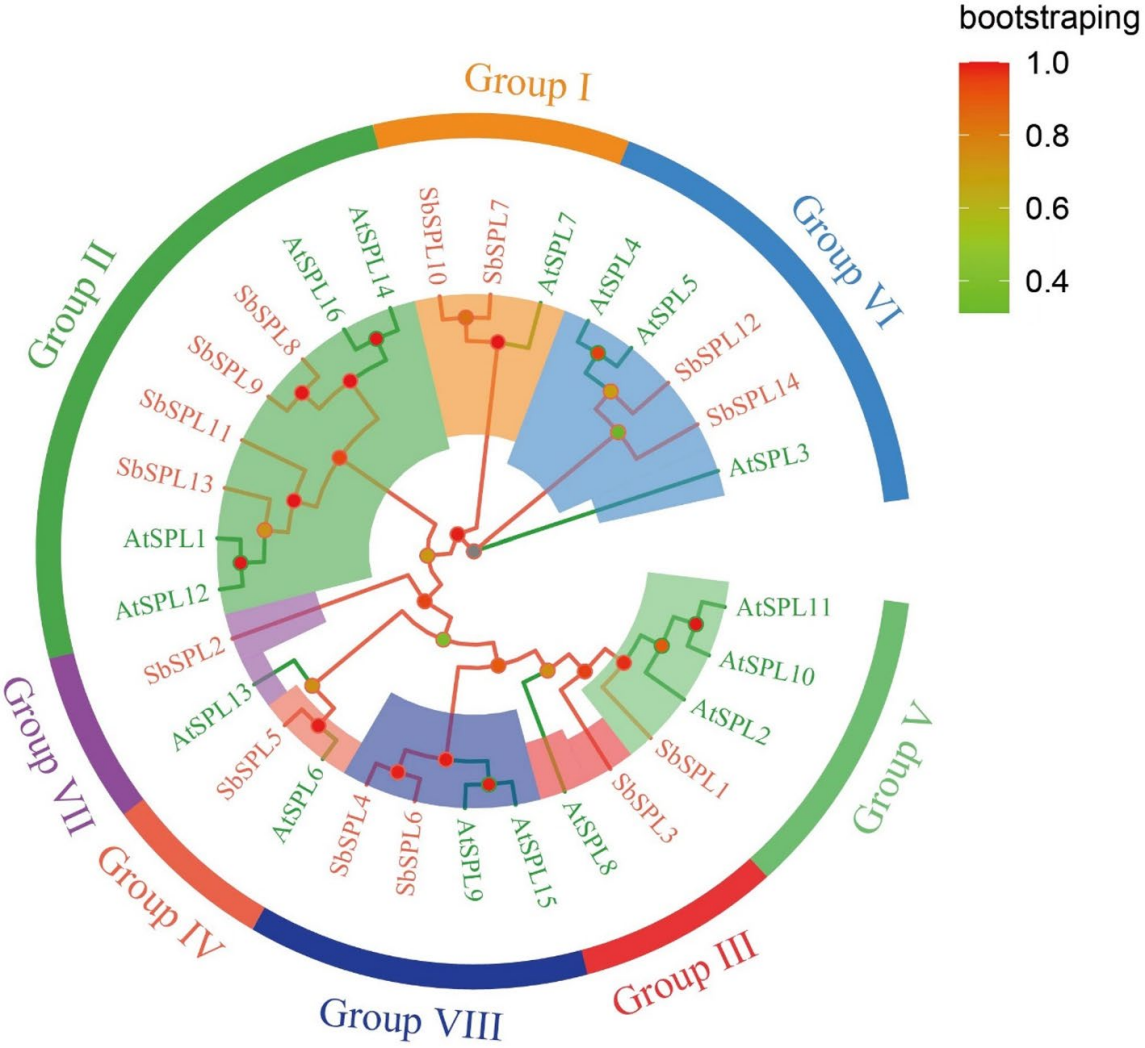
Phylogenetic analysis of *S. baicalensis* and *A. thaliana* SPLs. Circles of different sizes and colors indicate the magnitude of the bootstrap value. The 8 main branches are individually marked with colored ranges

Fourteen SbSPL proteins were phylogenetically analyzed together with *A. thaliana* AtSPLs and divided into 8 groups (I–VIII) according to the grouping method of AtSPLs (Fig. 1). Groups III, IV, V and VII had the fewest members with one each, namely, SbSPL3, SbSPL5, SbSPL1 and SbSPL2. Group II had the most members (4), namely, SbSPL8/9/11/13. Groups I, VI and VIII each had 2 members, namely, SbSPL7/10, SbSPL12/14 and SbSPL4/6.

The integrity of the SBP domain (~ 76 aa) reflects the functionality of SbSPLs. As shown in Figure S2, the structural domain contained highly conserved sequences, such as CQQC, SCR, and RRR. Most SbSPLs contained 2 zinc finger domains (Zn-1 and Zn-2) and 1 NLS. Only Zn-1 of SbSPL2 (group VII) and Zn-2 of SbSPL4 (group VIII) were missing. Zn-1 of all Group I members (SbSPL7/10) was CCCC, while that of other members was CCCH. All SbSPLs contained a highly conserved NLS at the C-terminus of the SBP domain. The NLS of SbSPL3/7 was RRRR and that of other SbSPLs was RRRK.

### Motif composition and gene structure

**Fig. 2:**
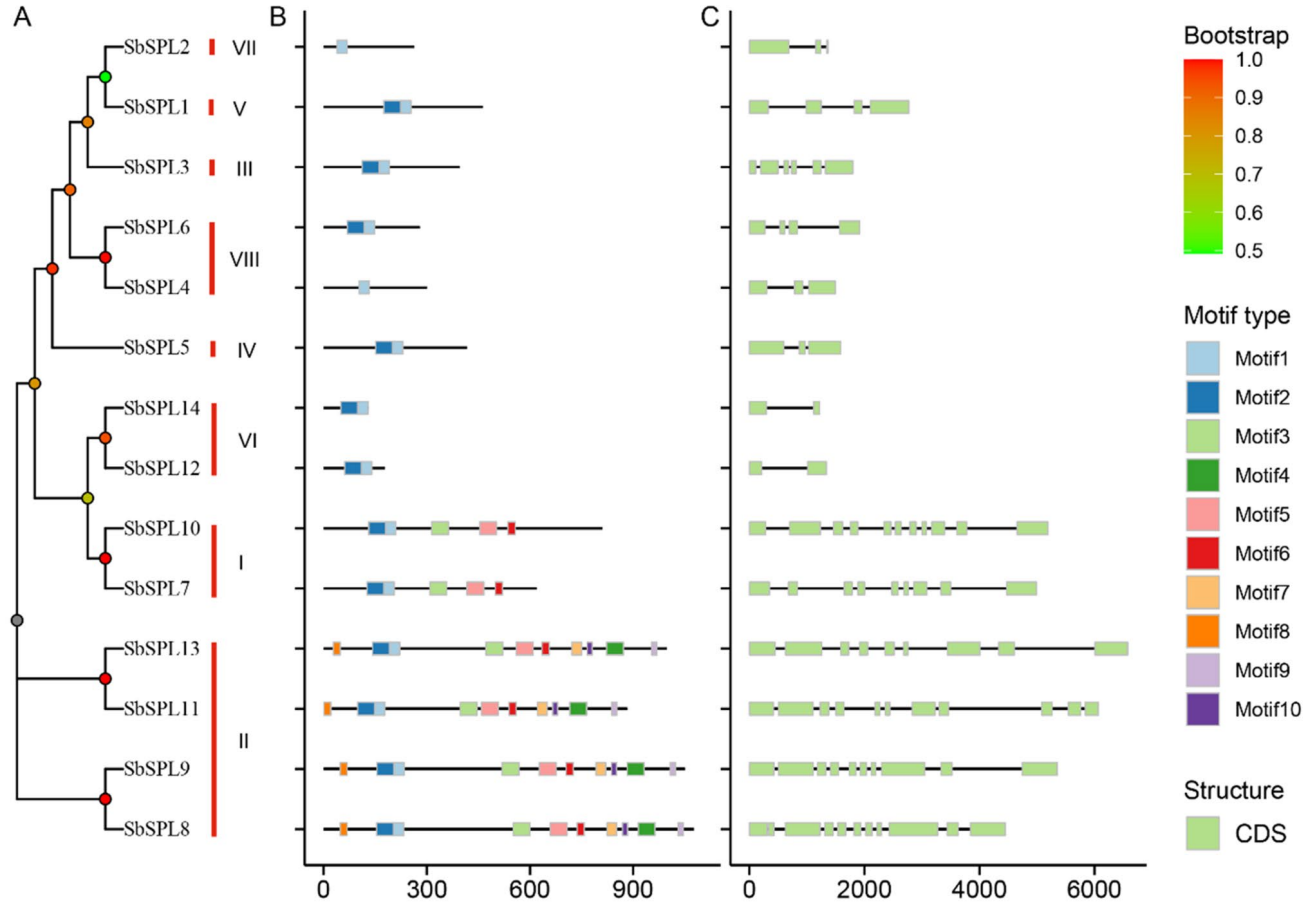
Phylogenetic relationships, conserved motif composition and gene structure. **A** Phylogenetic tree. **B** Motif pattern of SbSPLs. The 10 colored boxes represent 10 different motifs, and their positions represent the positions on the protein. **C** Gene structure. CDS and introns are represented by green rectangles and black single lines, respectively

Motif and structure analysis further revealed the conservation of *SbSPLs*. We identified 10 conserved motifs (motif 1—motif 10) (Fig. 2B and Figure S3). Except for SbSPL2/4, which only had motif 1, the other members all contained motifs 1 and 2. Meanwhile, all members of Group I (SbSPL7/10) contained motifs 1, 2, 5 and 6; all members of group II (SbSPL8/9/11/13) contained all motifs. Similarly, members of Groups I and II contained the most introns (8–10) (Fig. 2C and Table S3). Groups III, IV, VI, VII and VIII members contain fewer introns (1–3). This suggested that genes with closer phylogenetic relationships have more similar structures.

### Analysis of cis-acting elements

**Fig. 3:**
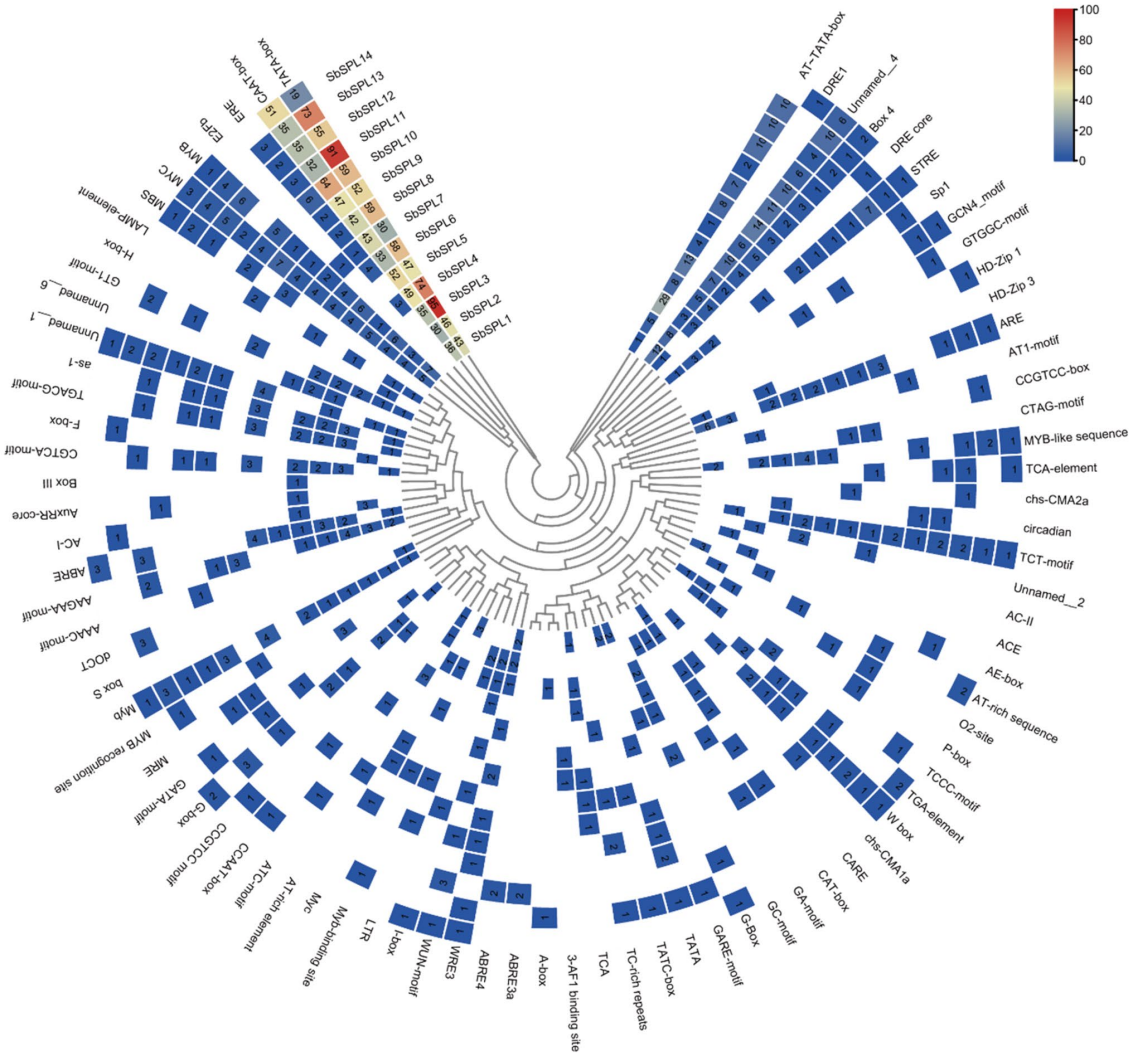
Prediction of cis-acting elements in *the SbSPL* promoter. The gradient color in the cell represents the number

Cis-acting element analysis was used to predict the potential biological functions of *SbSPLs*. The TATA box was the most numerous core promoter (801), followed by the CAAT box (584), and they were distributed in all SbSPL promoter regions (Fig. 3, Figure S4 and Table S4). Nine hormone-responsive cis-acting elements were identified. Most *SbSPLs* were related to the ABA pathway, and 8 *SbSPL* promoters contained 18 ABREs. In addition, 2 types of JA-responsive cis-acting elements (CGTCA motif and TGACG motif) were distributed in 8 *SbSPL* promoters, 14 of each type. The SA-responsive cis-acting element (TCA-element) was the least abundant, with only 4. Some stress-responsive cis-acting elements were also found in promoters. The promoters of *SbSPL5/8/9/10/13* all had TC-rich repeats of defense and stress response elements. We also found 12 drought-induced elements (MBS), including 3 in *SbSPL8/9*, 2 in *SbSPL10/13*, and 1 in *SbSPL12/14*. Only *SbSPL8/9/10* contained 1 low-temperature response element (LTR).

### Replication, collinearity and Ka/Ks analysis

**Fig. 4:**
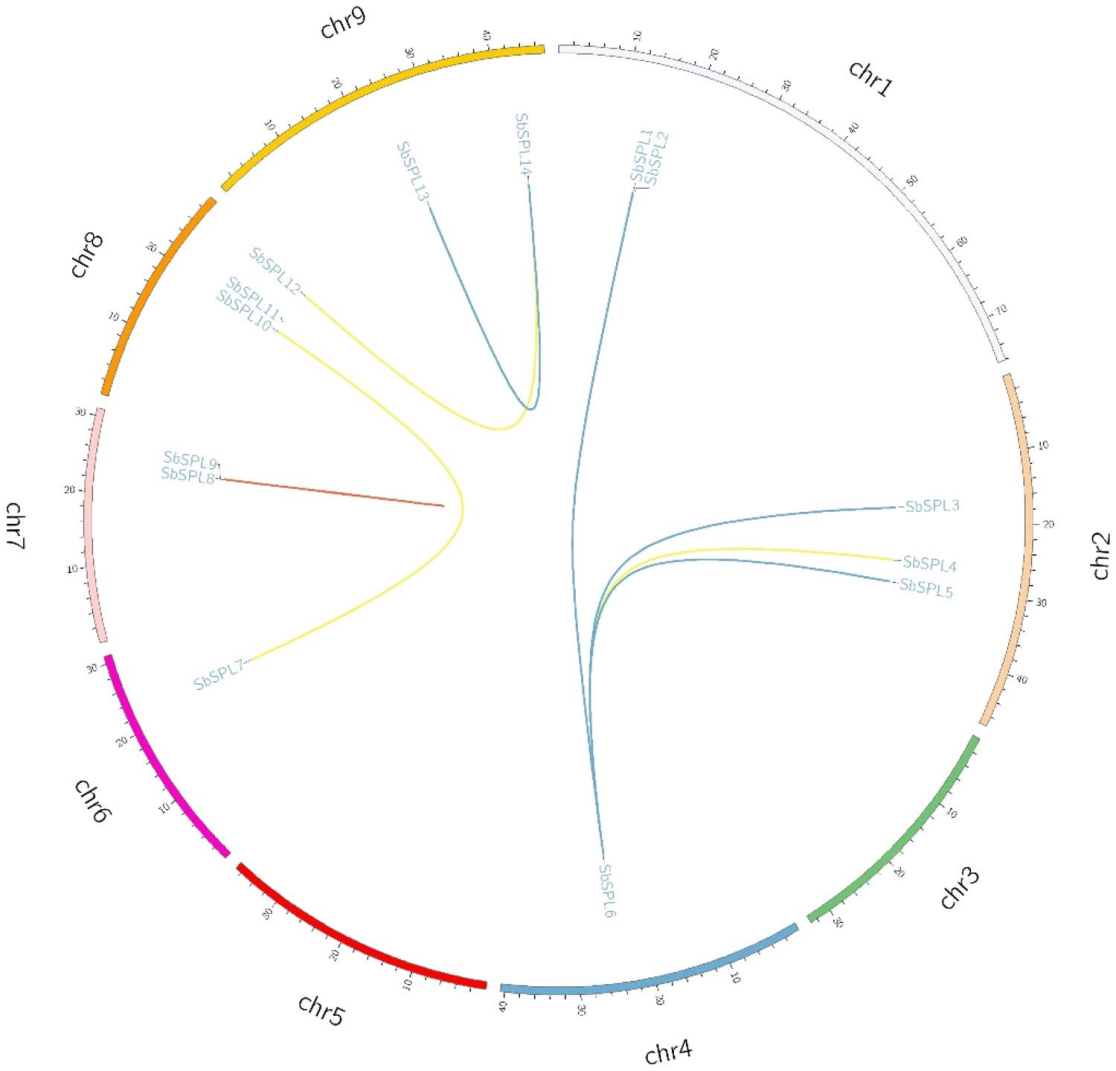
Collinearity analysis of SbSPLs. The short lines of different colors inside the circle represent different gene duplication events, in which the short red lines represent proximal repeats; the short orange lines represent fragment repeats; the short green lines represent transposition repeats; and the short purple lines represent tandem repeats (none)

We analyzed the mechanisms of *SbSPL* expansion and evolution. Except for *SbSPL2/11*, the other *SbSPLs* participated in gene duplication events to form 8 gene pairs, including proximal duplication (1), segmental duplication (3) and transposable duplication (4) (Fig. 4). Segmental duplications and transposable duplications were the main causes of *SbSPL* expansion. The Ka/Ks values of all duplicate genes were less than 1 (0.3518–0.7839), indicating that SbSPLs experienced negative selection (Table S5). The divergence time of the duplicated *SbSPLs* ranged from 52.24 to 104.77 Mya.

**Fig. 5:**
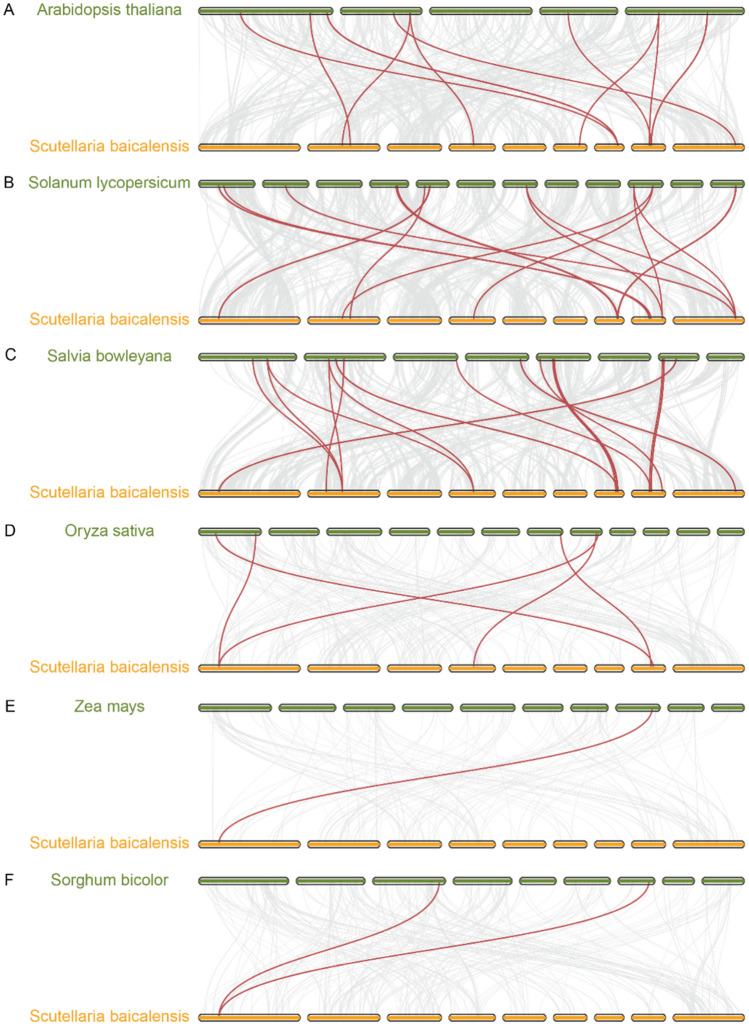
Synteny analysis of *SPLs* between *S. baicalensis* and *A. thaliana* (**A**), *S. lycopersicum* (**B**), *S. bowleyana* (**C**), *S. bicolor* (**D**), *O. sativa* (**E**), and *Z. mays* (**F**). Syntenic gene pairs are highlighted with red lines. Chromosomes are arranged in ascending order from left to right, with numbering omitted

The syntenic gene pairs between species were analyzed. *S. baicalensis* had 13, 11, and 6 gene pairs with *S. miltiorrhiza*, *S. lycopersicum*, and *A. thaliana,* respectively (Fig. 5A–C). *S. baicalensis* had fewer syntenic gene pairs with the monocots *Z. mays*, *S. bicolor*, and *O. sativa*, with 1, 2, and 4 pairs, respectively (Fig. 5D–F). Orthologous genes of *SbSPL4/6/10/14* were found in all dicots but not in monocots, indicating that these genes were formed after species divergence.

### Tissue-specific expression analysis based on RNA-seq

**Fig. 6:**
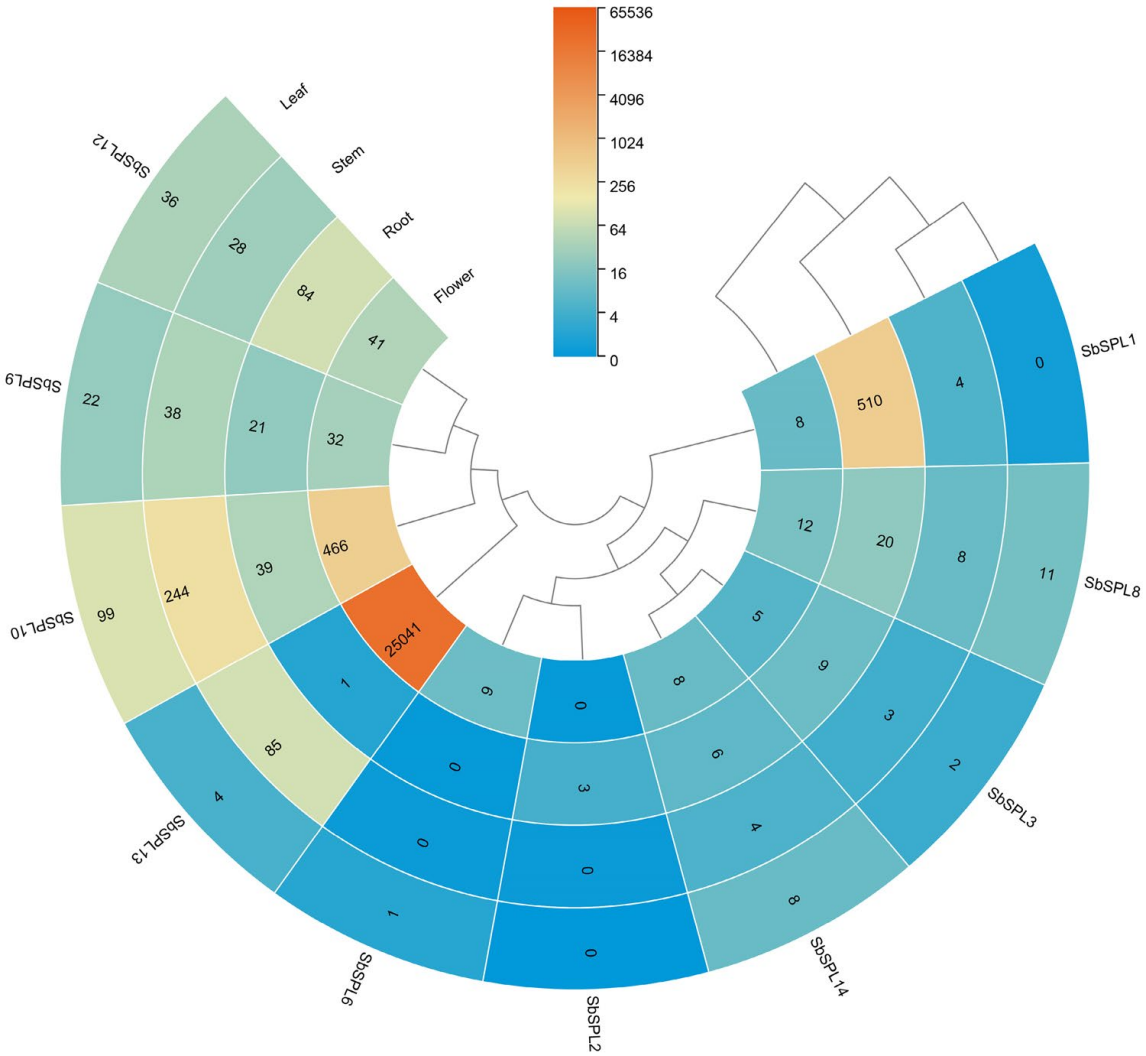
Expression levels of *SbSPLs* in 4 tissues. In cells, orange indicates high expression, and blue indicates low expression

We analyzed the expression pattern of *SbSPLs* in different tissues (root, stem, leaf, and flower) according to the published RNA-seq dataset (Fig. 6 and Table S6). Expression data for *SbSPL4/5/7/11* was not obtained, suggesting that they may be pseudogenes or have specific spatiotemporal expression patterns not explored in this database. Among the 10 genes whose expression could be detected, *SbSPL10* was most highly expressed in leaves and stems, while *SbSPL1* and *SbSPL13* were most highly expressed in roots and flowers, respectively. In addition, we also found that *SbSPL9/10/12* were highly expressed in the 4 tissues.

### Expression analysis of SbSPLs under abiotic stress and hormone treatment based on qPCR

**Fig. 7:**
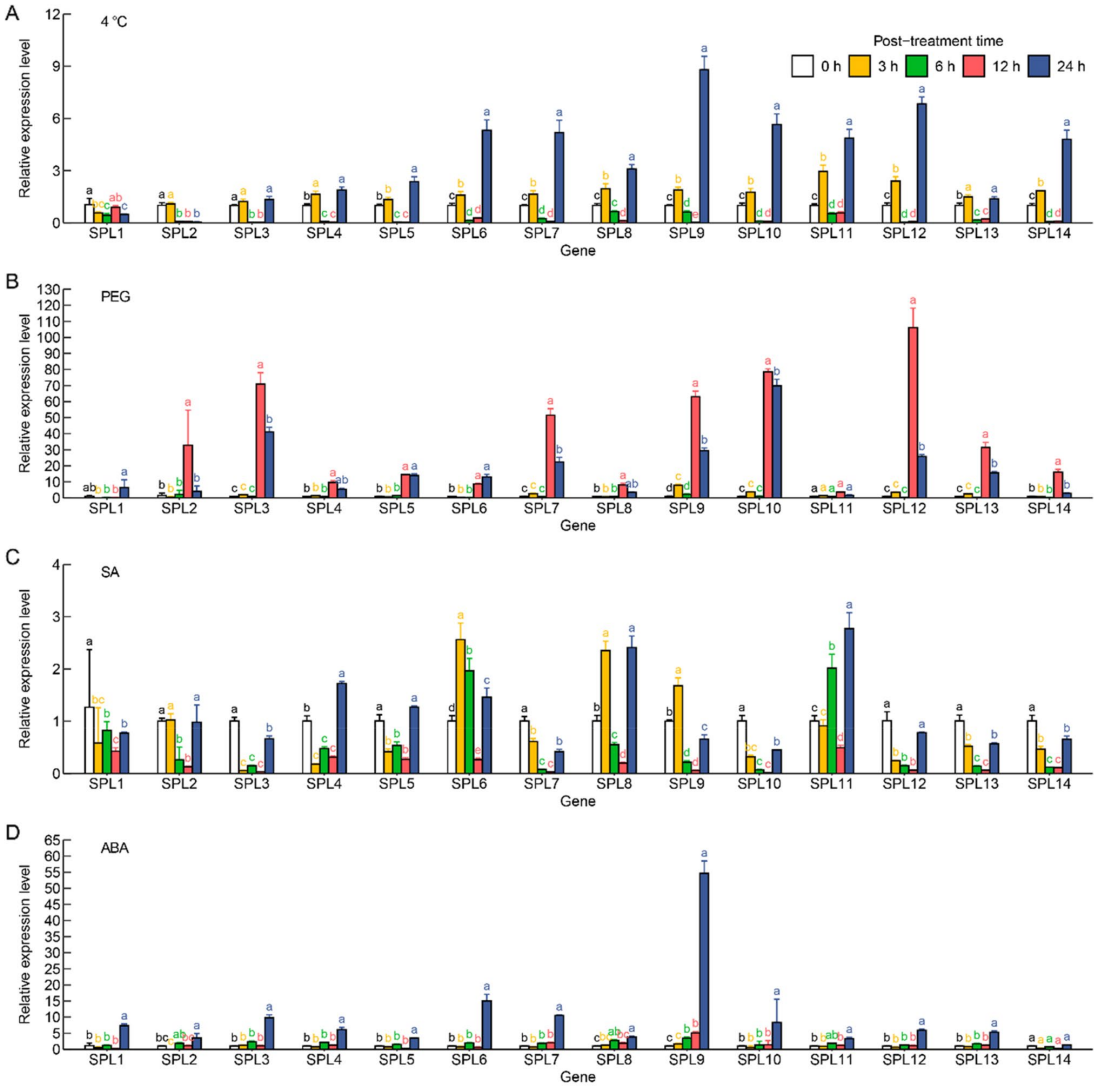
Expression pattern of *SbSPLs* under abiotic stress and hormone treatment. Analysis of *SbSPL* expression levels under 4 °C (**A**), PEG (**B**), SA (**C**) and ABA (**D**) treatments. Different letters represent significantly different expression levels in the same treatment and gene at different times

To explore the potential functions of *SbSPLs*, the expression levels of 14 *SbSPLs* were analyzed at 5 time points (0, 3, 6, 12, and 24 HAT) under 4 °C, PEG, SA and ABA treatment. *SbSPLs* responded to 4 °C treatment earlier. The expression of 10 *SbSPLs* (*SbSPL4/6/7/8/9/10/11/12/13/14*) was upregulated at 3 HAT, downregulated at 6 and 12 HAT, and peaked at 24 h (Fig. 7A). In response to PEG, the expression of 11 *SbSPLs* (*SbSPL2/3/4/5/7/8/9/10/12/13/14*) began to significantly increase and peaked at 12 HAT (Fig. 7B). *SbSPL7/9/10/12* were all found to respond extremely strongly to 4 °C and PEG. They increased by 4.18, 7.79, 4.62, and 5.79 times for 24 HAT at 4 °C and by 50.21, 61.48, 76.99, and 104.57 times for 12 HAT under PEG, respectively (Fig. 7A and B). This indicates the potential role of *SbSPL7/9/10/12* in responding to a broad spectrum of abiotic stresses. Comparison of the fold differences also showed that the role of *SbSPL7/9/10/12* under drought stress cannot be ignored. In addition, we also found that most *SbSPLs* responded to SA (14) and ABA (13) (Fig. 7C and D). Similar to 4 °C, 13 SbSPLs (*SbSPL1*–*SbSPL13*) were significantly upregulated and peaked at 24 HAT under ABA treatment, among which the expression levels of *SbSPL3/6/7/9* were significantly increased by 8.77, 14, 9.5 and 53.44 times (Fig. 7D). In the SA treatment, the expression of *SbSPL6/9* peaked at 3 HAT, increasing by 1.55 and 0.68 times, respectively; the expression of *SbSPL4/8/11* peaked at 24 HAT, increasing by 0.72, 1.4 and 1.77 times, respectively (Fig. 7C). The expression levels of *SbSPL1/3/4/5/7/10/12/13/14* were significantly downregulated at 3 HAT (Fig. 7C). Compared with SA, the response of *SbSPLs* to ABA was more regular and severe, which indicated the effectiveness of ABA in stimulating the function of *SbSPLs*.

Cluster analysis of time series was used to further search for similarity patterns in the expression of *SbSPLs* (Figure S5). In all treatments, when the number of clusters was 2, the membership value was higher than 0.5. Under abiotic stress (4 °C and PEG), *SbSPL2/3/13*, *SbSPL5/6/10*, and *SbSPL4/7/8/9/11/12/14* showed consistent expression patterns (Figure S5 A and B). Under hormone-treated conditions (SA and ABA), the expression patterns of *SbSPL2/8/11*, *SbSPL3/4/7/10/12/13*, *SbSPL5/14*, and *SbSPL6/9* were similar (Figure S5C and D). *SbSPL3/13* and *SbSPL4/12* were always similar in all treatments (Figure S5). This showed the functional consistency of *SbSPLs*.

### Prediction of miR156/157 s regulating SbSPLs

**Fig. 8:**
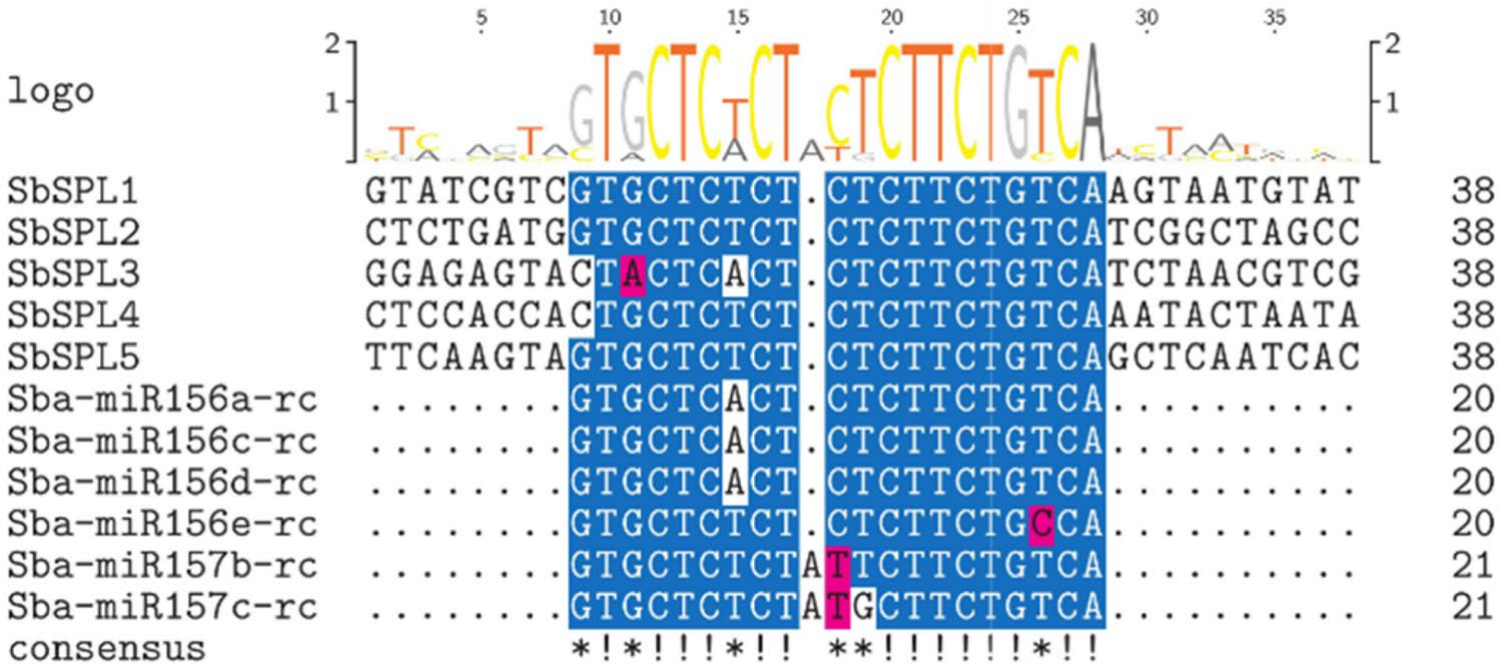
Multiple sequence alignment of the reverse complementary sequences of Sba-miR156/157 and *SbSPLs*. Amino acid residues that are highly conserved in different sequences are marked with a blue background and an exclamation mark below; amino acid residues that are similar in different sequences are marked with a red background and an asterisk below. LOGO is above the aligned sequence

We found 5 potential *miR156s* and 3 potential *miR157s*. They were distributed on chr 1 (*Sba-miR157a/b*), chr 2 (*Sba-miR156a/b*), chr 4 (*Sba-miR156c/d*), chr 5 (*Sba-miR157c*) and chr 9 (*Sba-miR156e*) and were named according to location (Figure S6). Sequence alignment analysis showed that the mature miR156/157 sequences of *S. baicalensis* were relatively conserved (Figure S7). The 8th base of the 3' end of miR156/157 s was U/A, and the 11th base contained U. Sba-miR157a/b/c inserted an A at the 11th base of the 5' end. In addition, the stems of the Sba-miR156/157 secondary structures all contained mature sequences (Figure S8). To analyze the posttranscriptional regulation pattern of *SbSPLs*, we predicted the *SbSPLs* targeted by Sba-miR156/157. Except for Sba-miR156b and Sba-miR157a, the other miR156/157 s may complementarily bind to the *SbSPLs* (Fig. 8 and Figure S9). SbSPL1–SbSPL5 had potential binding sites for Sba-miR156a/c/d/e, and miR156/157 complementary sites were relatively conserved among these SbSPLs (Fig. 8). Sequence differences were mainly limited to the 1/3/7 nucleotides of the complementary sequence.

### Prediction of *SbSPLs* on the regulation of phenylpropanoid and JA pathways

**Fig. 9:**
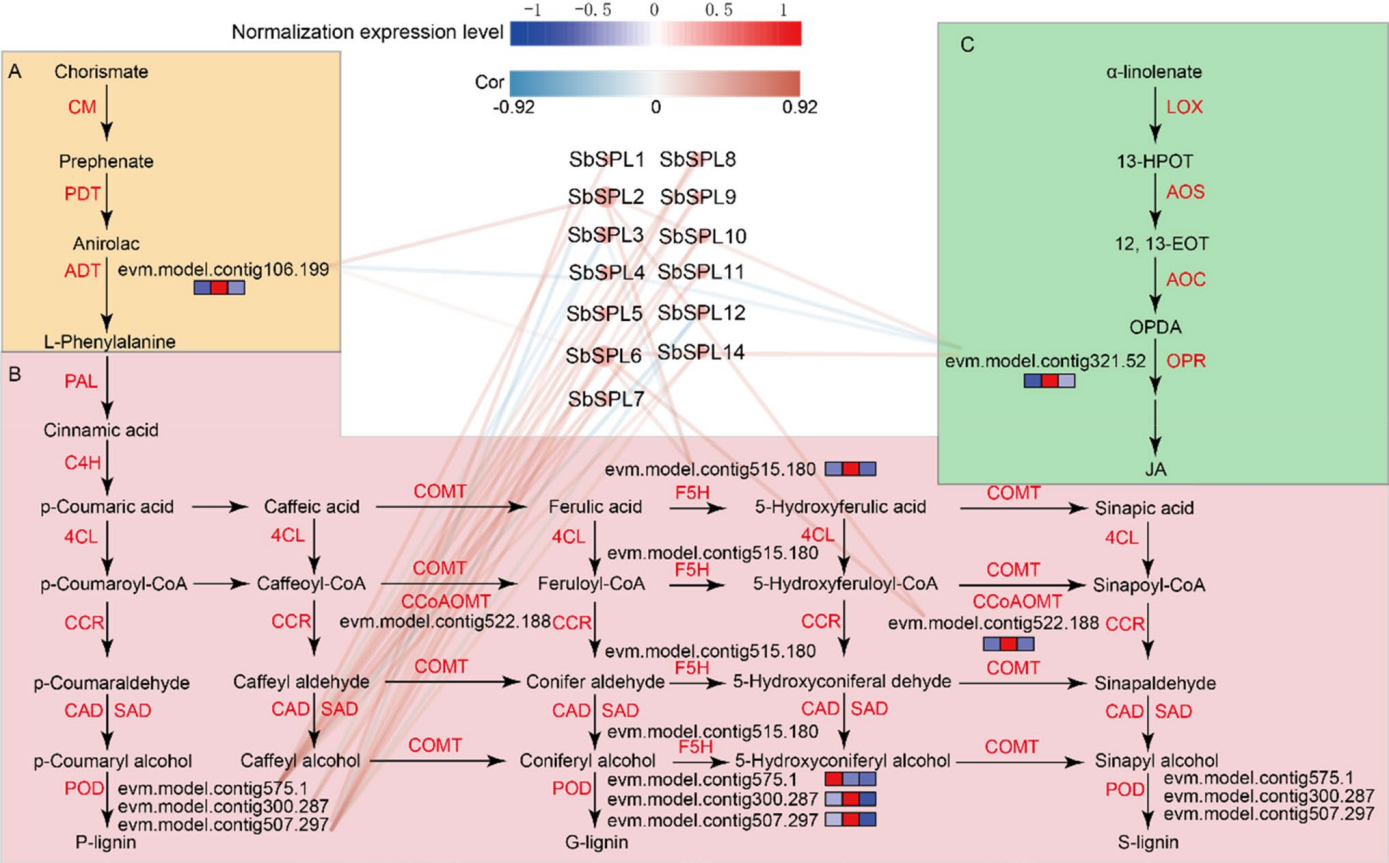
Regulatory network analysis. A Regulation of L-Phe biosynthesis by *SbSPLs*. B Regulation of lignin biosynthesis by *SbSPLs*. C Regulation of JA biosynthesis by *SbSPLs*. The red and blue lines represent positive and negative correlations, respectively. In the heatmap next to the gene name, red and blue represent higher and lower expression levels, respectively

The 30,100 CDSs of *S. baicalensis* were submitted to the KEGG website, and *A. thaliana* was used as the reference species to determine metabolic pathways of *S. baicalensis* homologous genes. The MEME plugin fimo was used to predict target genes of *SbSPLs*, and potential downstream genes with similar expression patterns to *SbSPLs* were screened by correlation analysis (r ≥ 0.5&p value ≤ 0.05; r ≤ -0.5&p value ≤ 0.05). SbSPL-2/4/6 could bind to the upstream promoter of *evm.model.contig106.199* (aeroate dehydratase, *ADT*), indicating that it may be involved in the synthesis of L-Phe (Fig. 9A and Table S7). The prediction results showed that the promoters of the JA synthesis pathway gene *evm.model.contig321.52* (12-oxo-phytodienoic acid reductase, *OPR*), the phenylpropane metabolism pathway-related genes *evm.model.contig515.180* (ferulate 5-hydroxylase, *F5H*) and *evm.model.contig522.188* (caffeoyl-CoA oxymethyltransferase, *CCoAOMT*) were the binding targets of SbSPL2/6 (Fig. 9C and Table S7). In addition, *POD* homologous genes (*evm.model.contig575.1*, *evm.model.contig300.287* and *evm.model.contig507.297*) had regulatory relationships with the rest of the genes except *SbSPL13* (Fig. 9B and Table S7).

## Discussion

### Characteristics and evolutionary mechanisms of *SbSPLs*

SPL TFs comprise a relatively small-scale family. Fourteen SbSPLs were identified from the *S. baicalensis* genome, and the number was similar to those of the dicotyledons *A. thaliana* (17) (Xu et al. 2016), *Z. jujuba* (18) (Shao et al. 2017), and *F. vesca* (14) (Xiong et al. 2018) and monocots *O. sativa* (19) (Xie et al. 2006) and *S. italica* (18) (Lai et al. 2022). The SPL sequences of *S. baicalensis* and the model species *A. thaliana* were highly conserved, and each group contained at least 1 *AtSPL* (Fig. 1). The sequence length (131–1076 aa) and MW (14.97 kDa -118.85 kDa) of SbSPL proteins varied greatly. This phenomenon was also observed in *Vaccinium corymbosum* VcSPLs (126–1072 aa; MW: 14.04–117.84 kDa) (Feng et al. 2023b), *Prunus avium* PavSPLs (162–1069 aa; MW: 18–118 kDa) (Sun et al. 2023) and *Nicotiana tabacum* NtSPLs (119–1001 aa; MW: 13.7–111.3 kDa) (He et al. 2023). SPL TFs diverged earlier than green algae (Guo et al. 2008) and initially formed two distinct lineages (clade I and clade II) in land plants. Clade I members had conserved structures, with more exons and longer protein sequences (Zhong et al. 2019). Groups I and II were consistent with clade 1, and the other groups could be placed in clade 2 (Fig. 2 and Table S2). Differentiation in genetic architecture partially explains the failure of SbSPL2/3/5 to localize to the nucleus.

The integrity and conservation of the SBP domain determine gene function (Xie et al. 2021). CCCH was mutated to CCCC in Zn-1 of SbSPL7/10 (Figure S2). The same changes occurred in *A. thaliana AtSPL7* and *S. italica SiSPL8* (Lai D et al. 2022) and *CgoSPL6*, *DchSPL16*, and *GelSPL8* of three orchids (Zhao et al. 2023), indicating that Zn-1 could exist in 2 types (CCCC and CCCH). The Zn-1 of SbSPL2 and the Zn-2 sequence of SbSPL4 were missing (Figure S2). Zn-1 of FmSPL9 and Zn-2 of FmSPL31/32 were also absent in *Fraxinus mandshurica* (He et al. 2022a). Domain changes or losses are not always negative and drive the expansion of gene families (Brand et al. 2013; Wei et al. 2012).

Segmental duplication, tandem duplication and transposable duplication are considered to be the three principles of the evolutionary model (Moore RC and Purugganan 2003). Tandem duplication and segmental duplication are considered to be the main causes of gene family expansion (Liu et al. 2011). The expansion of *SbSPLs* mainly relied on transposable duplication (4) and segmental duplication (3) (Fig. 4 and Table S5). Duplications of Group I members (*SbSPL7* and *SbSPL10*) and II (*SbSPL8* and *SbSPL9*) had lower ka/ks (0.3350 and 0.3518) than other duplications (Table S5). Similar to previous results, the evolutionary speed of genes in Clade I seems to be slower (Zhu et al. 2020). The *SbSPL* repeat genes were subjected to purifying selection pressure (ka/ks < 1) (Table S5), ensuring the conservation of the protein. Transposable duplication genes had higher ka/ks (0.6387–0.7839) (Table S5). They faced higher selection pressure and were more evolutionarily active and were more likely to produce new functions or become pseudogenes (Wu et al. 2019). There was a strong positive correlation between species affinities and the synteny of SPLs. *S. baicalensis* had more orthologous *SPLs* with dicots than with monocots and had the most orthologous *SPLs* with the same family plant, *S. bowleyana* (13) (Fig. 5).

### Potential functions of the *SbSPLs*

*SPLs* can regulate the development and growth of plant branches, flowers and other organs. Eight *T. aestivum TaSPLs* were found to be highly expressed in stems and inflorescences (Zhu et al. 2020). *A. thaliana AtSPL2/9/11/13/15* promoted floral meristem recognition and floral induction (Xu et al. 2016). *A. thaliana AtSPL1* and *AtSPL12* were highly expressed in inflorescences, and their overexpression enhanced the heat tolerance of inflorescences (Chao et al. 2017) and belonged to group II with *SbSPL13* (Fig. 1). *SbSPL13* was extremely highly expressed in flowers, followed by *SbSPL10*, which was highly expressed in both flowers and stems (Fig. 6). The function of *SbSPL13/10* in flower and stem development has research value.

Analysis of promoter cis-elements indicated that *SbSPLs* could be involved in abiotic stress and hormone responses (Fig. 3 and Table S4). SPL functions have been studied. Overexpression of *OsSPL7* can improve the cold tolerance of *O. sativa*, while the knockout line showed strong low temperature sensitivity and growth inhibition (Hoang et al. 2019). AtSPL9 combined with the promoter of *CBF2* to positively regulate cold resistance in *A. thaliana*, and miR156 balanced resistance responses and vegetative growth (Zhao et al. 2022). *SbSPL4/6* belonged to group VIII with *AtSPL9* (Fig. 1) and had a significant response to low temperature (Fig. 7A), indicating potential resistance. In addition, *SbSPL4/6/7/8/9/10/11/12/13/14* showed a wave-like (increase–decrease-increase) expression pattern over time at low temperature (Fig. 7A), indicating that they could be regulated by factors such as miR156/157 to maintain the stability of growth and resistance responses. More notably, *SbSPL7/9/10/12* responded strongly not only to low temperature but also to drought stress (Fig. 7B). Their identification provides candidate resistance genes to a broad spectrum of abiotic stresses. Several *SPLs* with drought resistance functions have been identified in multiple plants. *MsSPL13* in *Malus sieversii* (Feng et al. 2023a), *MsSPL8* in *Medicago truncatula* (Gou J et al. 2018), *MeSPL9* in *Manihot esculenta* (Li et al. 2022b), and *MsSPL9* in *M. sativa* (Hanly et al. 2020) negatively regulate drought resistance, while *MsSPL13* in *M. sativa* (Arshad et al. 2017) and *MiSPL13* in *Mangifera indica* (Zhu et al. 2022) enhance drought resistance. *SbSPL1* is an orthologous gene of *M. sieversii MsSPL13* (Feng et al. 2023a) and *M. sativa MsSPL13* (Wang et al. 2019). However, the expression level of *SbSPL1* did not change significantly under drought, indicating the species specificity of *SPL* functions. *SbSPL2*, *SbSPL3* and *SbSPL4/6* are orthologous genes of *MiSPL13* (Zhu et al. 2022), *MsSPL8* (Gou et al. 2018) and *MeSPL9* (Li et al. 2022b), respectively. Their expression levels increased significantly at 12 HAT under drought, indicating potential function.

SA and ABA are important signaling molecules for plant physiological and morphological responses. They can regulate ROS levels and activate the expression of TFs and resistance genes during biotic and abiotic stress (Sah et al. 2016; Yang et al. 2023). *SbSPLs* differentially responded to SA; the expression levels of *SbSPL1/3/4/5/7/10/12/13/14* were significantly downregulated at 3 HAT, while the expression levels of *SbSPL6/9* and *SbSPL4/8/11* were upregulated and reached a peak at 3 HAT and 24 HAT, respectively (Fig. 7C). This suggested their differentiated functions. The ABA pathway is the core of plant drought resistance, and ABREs are conserved in the promoters of drought-responsive genes (Muhammad Aslam et al. 2022; Waadt et al. 2022). The promoters of 8 *SbSPLs* contained ABREs (18) (Fig. 3, Figure S4 and Table S4), and 13 SbSPLs were upregulated in response to ABA (Fig. 7D), indicating possible drought resistance. Most *SbSPLs* responded to drought earlier than to ABA (Fig. 7A and Fig. 7D, Figure S5). It is speculated that there is a complex regulatory mechanism between drought, ABA and *SbSPLs*. Furthermore, the strong response of *SbSPL9* to stress and ABA suggests its importance (Fig. 7). In summary, our study showed that SbSPLs respond to abiotic stress and hormones, which provides a reference for subsequent research on gene functions.

### The potential regulation of *SbSPLs* by miR156/157 and its potential impact on the accumulation of downstream substances

*SPLs* in plants often coordinate with miR156/157 to function together. Eleven of the 17 *AtSPLs* were miR156 targets in *A. thaliana* (Xu et al. 2016). Eleven *OsSPL* genes were predicted targets of OsmiR156 in *O. sativa* (Xie et al. 2006). Similar to previous studies, *SbSPL1*—*SbSPL5* were potential target genes of Sba-miR156/157 (Fig. 8 and Figure S9).

SPL TFs serve as activators or inhibitors of gene expression and are involved in regulating the synthesis of plant metabolites (Kajla et al. 2023). *Morus alba MnSPL7* increased the expression levels of catechin synthesis genes (f3’h, DFR and LAR) by promoting the transcription of *MnTT2L2* (Li et al. 2022a). *Artemisia annua AaSPL2* and *DBR2* act synergistically at the “GTAC” cis-element in the *DBR2* promoter to mediate transcriptional activation of *DBR2* in response to JA, thereby increasing artemisinin content (Lv et al. 2019). In this study, *SbSPL2/4/6* was closely related to the expression of *evm.model.contig106.199* (*ADT*), a key gene for L-Phe synthesis (Fig. 9A and Table S7). L-Phe is the starting material in the synthesis pathway of phenylpropanoids and flavonoids, which is bound to affect flavonoid accumulation (Dong and Lin 2021). In addition, lignin is important for plant environmental adaptability. Several recent studies have reported lignin to be involved in plant responses to stress. *MeRAV5* activates cassava lignin accumulation and improves drought resistance (Yan et al. 2021), and knocking out the *F5H* gene to reduce S-lignin/G-lignin improved *S. baicalensis* resistance to *Brassica napus* and increased stem strength (Cao et al. 2022). Our results predicted that the *F5H* (*evm.model.contig515.180*) and *CCoAOMT* (*evm.model.contig522.188*) genes were positively regulated by *SbSPL2/6*. The opposite state appeared in the regulation of *F5H* (*evm.model.contig515.180*) by *SbSPL3* (Fig. 9B and Table S7). We speculate that *SbSPLs* regulate key genes of lignin synthesis and affect the ratio of G-ligin and S-ligin, thereby affecting the stress resistance of *S. baicalensis*. Notably, *PODs* (*evm.model.contig575.1*, *evm.model.contig300.287* and *evm.model.contig507.297*) appeared to be more complexly regulated by *SbSPLs* (Fig. 9B and Table S7). As a key enzyme system in the oxidative stress response, POD has unique functions in controlling various aspects of plant growth, development, cell metabolism and defense signal transduction (Lee et al. 2018). Another important pathway in the defense response is the synthesis and signal transduction of JA. Key gene expression of *SPL7*-regulated JA synthesis in *A. thaliana* has been demonstrated (Yan et al. 2017). Likewise, the miR156-SPL9 module positively regulated *A. thaliana* resistance to *Botrytis cinerea* through the JA pathway (Sun et al. 2022). In our study, the binding of SbSPL2/4/6/11 to the *OPR* (*evm.model.contig321.52*) promoter (Fig. 9C, Table S7) and similar expression patterns might provide another case.

## Conclusion

In this study, 14 *SbSPLs* with SBP-box domains were identified and divided into 8 groups based on phylogenetic analysis. The genetic structures of *SbSPLs* in the same group were highly similar. Five *SbSPLs* contained complementary sequences of miR156/157 s and could be used as potential targets. In addition, *SbSPLs* contained abiotic stress- and hormone-responsive cis-acting elements. Gene expression pattern analysis based on transcriptional profiling and qPCR demonstrated that *SbSPLs* potentially regulated the development and growth of organs, and they (especially *SbSPL9*) responded to low temperature, drought, SA, and ABA. These results increase the understanding of the evolution and biological importance of *SbSPLs* and provide a reference for revealing the functional characteristics of *SbSPLs* and the mechanism for genetic improvement of plants under stress.

### Supplementary Information

Below is the link to the electronic supplementary material.

Supplementary file1 (DOCX 107 KB)

Supplementary file2 (JPG 246 KB)

Supplementary file3 (JPG 6287 KB)

Supplementary file4 (JPG 3054 KB)

Supplementary file5 (JPG 97 KB)

Supplementary file6 (JPG 1484 KB)

Supplementary file7 (JPG 509 KB)

Supplementary file8 (JPG 838 KB)

Supplementary file9 (JPG 2950 KB)

Supplementary file10 (JPG 225 KB)

## Data Availability

All data generated or analyzed in this study are included in this published article and its Supplementary Material. The datasets generated and analyzed during the current study are available from the corresponding author on reasonable request. The analysis websites used in this study are as follows: The DNA and protein sequence information of *S. baicalensis* was downloaded from the National Genomics Data Center (https://ngdc.cncb.ac.cn/, accession number: PRJCA009554). Raw RNA-Seq data for roots, stems, leaves, and flowers were downloaded from the European Nucleotide Archive (https://www.ebi.ac.uk/ena/browser/home, accession number: PRJNA263255). All clean sequence read data of *S. baicalensis* roots, stems, and leaves were deposited in the NCBI SRA database (https://www.ncbi.nlm.nih.gov/sra, accession number: PRJNA515574). Pfam database (http://pfam.sanger.ac.uk/), SMART (http://smart.embl.de/), ExPASy Bioinformatics Resource Portal (https://web.expasy.org/compute_pi/), ProtComp 9.0 (http://linux1.softberry.com/berry.phtml?topic=protcomppl&group=programs&subgroup=proloc), PLACE database (https://bioinformatics.psb.ugent.be/webtools/plantcare/html/), MFold (http://www.mfold.org/), Kyoto Encyclopedia of Genes and Genomes database (https://www.genome.jp/kegg/).
