# Supplementary material for: Genome-wide identification, stress- and hormone-responsive expression characteristics, and regulatory pattern analysis of Scutellaria baicalensis SbSPLs
Source: Plant Mol Biol. 2024 Feb 16;114(2):20. doi: 10.1007/s11103-023-01410-z (PMC10873456; doi:10.1007/s11103-023-01410-z)
Supplement: Supplementary file 1 — Supplementary file1 (DOCX 107 KB) [file 11103_2023_1410_MOESM1_ESM.docx]

| Table S1 Primers of sequences | | |
| --- | --- | --- |
| Gene | Forward primer (5`- 3`) | Reverse primer (5`- 3`) |
| *SbActin* | TTGATCTTGCTGGTCGTGATCTCA | TGTTTCTAGCTCTTGCTCGTAGTCG |
| *SbSPL1* | GTTCCATCACCTGTCCGA | CATTTGTTGCCTTCCTCC |
| *SbSPL2* | ACTCACTCACCCGAAACT | AACCCATCCGAATACATA |
| *SbSPL3* | TGAGAGAAAGCAGAATGT | GGTTTGAGTTGTGGATAG |
| *SbSPL4* | CCACCTCAAGGCCCGTTATT | CCGGGGAATTGACCAATGGA |
| *SbSPL5* | TATCAGCAAAAAGACAGC | AACAATAACCACAGGAGT |
| *SbSPL6* | AAGCCGCCGCCTGGATCT | CCAGGAAGGGCAGACAAA |
| *SbSPL7* | CCCCACCCCTTCATCCCT | GCGTCCGCTTCTTACCCG |
| *SbSPL8* | TCTTCTTTGTTTGGGGTC | GTTCAAGGGGTGGATTTC |
| *SbSPL9* | GCTCTACCTTTTCATTCA | TTACCTCTGCTACCACTC |
| *SbSPL10* | CGGGAAACGAGCAAAGTC | GCTGAGGCACGAACCACC |
| *SbSPL11* | GAGGAAACTCAATAAGGG | TGGAATGAACAAAAGACC |
| *SbSPL12* | TCACGAAGGAGAAGATGA | TTGGCATGGACCTCACAC |
| *SbSPL13* | ATCTCACTCTGAACCTCG | ATGATAATCTTTCGCCTT |
| *SbSPL14* | AAGCAAAGAAGGAAAAACGA | CTACCTGACAAGAACGAAGT |

| **Table S2 The predicted features of the 14 SbSPL TFs** | | | | | | | | | | | | | | | | |  |
| --- | --- | --- | --- | --- | --- | --- | --- | --- | --- | --- | --- | --- | --- | --- | --- | --- | --- |
| **Serial NO.** | **Gene Name** | **Accession Number** | | **ORF Length (bp)** | **Protein** | | | | **Chr.** | | **Group** | **Instability Index** | **Aliphatic Index** | **Grand Average of Hydropathicity** | **Subcellular Localization** | |  |
|  |  | **Ensemble transcript** | |  | **Number of Amino Acid (aa)** | **Molecular Weight (kDa)** | | **Theoretical pI** |  |  |  |  |  |  | **Integral Prediction of protein location** | **score** |  |
| 1 | SbSPL1 | evm.model.contig168.565 | | 1389 | 463 | 50.7 | | 8.59 | 1 | | V | 55.5 | 65.33 | -0.577 | Nuclear | 7.3 |  |
| 2 | SbSPL2 | evm.model.contig168.577 | | 792 | 264 | 28.95 | | 7.14 | 1 | | VII | 47.5 | 64.58 | -0.614 | Extracellular | 2.5 |  |
| 3 | SbSPL3 | evm.model.contig539.9 | | 1188 | 396 | 44.61 | | 7.94 | 2 | | III | 59.71 | 63.26 | -0.679 | Extracellular | 3 |  |
| 4 | SbSPL4 | evm.model.contig273.92 | | 903 | 301 | 32.4 | | 8.69 | 2 | | VIII | 55.98 | 51.93 | -0.729 | Nuclear | 8.8 |  |
| 5 | SbSPL5 | evm.model.contig370.303 | | 1251 | 417 | 46.35 | | 6.61 | 2 | | IV | 57 | 69.21 | -0.554 | Extracellular | 2.6 |  |
| 6 | SbSPL6 | evm.model.contig269.130 | | 840 | 280 | 30.39 | | 8.93 | 4 | | VIII | 60.49 | 48.07 | -0.814 | Nuclear | 8.3 |  |
| 7 | SbSPL7 | evm.model.contig464.49 | | 1857 | 619 | 69.13 | | 6.4 | 6 | | I | 63.3 | 79.69 | -0.475 | Nuclear | 7.3 |  |
| 8 | SbSPL8 | evm.model.contig420.12 | | 3228 | 1076 | 118.85 | | 8.12 | 7 | | II | 52.8 | 74.02 | -0.485 | Nuclear | 7.5 |  |
| 9 | SbSPL9 | evm.model.contig420.14 | | 3153 | 1051 | 116.45 | | 8.27 | 7 | | II | 50.9 | 78.91 | -0.427 | Nuclear | 7.4 |  |
| 10 | SbSPL10 | evm.model.contig155.11 | | 2433 | 811 | 90.29 | | 6.21 | 8 | | I | 54.55 | 77.68 | -0.391 | Nuclear | 7.3 |  |
| 11 | SbSPL11 | evm.model.contig155.257 | | 2646 | 882 | 98.23 | | 7.68 | 8 | | II | 55.98 | 79.92 | -0.456 | Nuclear | 7.4 |  |
| 12 | SbSPL12 | evm.model.contig106.94 | | 534 | 178 | 20.04 | | 9.33 | 8 | | VI | 59.01 | 48.26 | -1.072 | Nuclear | 8.9 |  |
| 13 | SbSPL13 | evm.model.contig126.70 | | 2994 | 998 | 111.14 | | 5.74 | 9 | | II | 49.38 | 83.12 | -0.366 | Nuclear | 7.6 |  |
| 14 | SbSPL14 | evm.model.contig455.712 | | 393 | 131 | 14.97 | | 7.67 | 9 | | VI | 86.98 | 43.28 | -1.198 | Nuclear | 9.1 |  |
| **Table S3:List of the 14 SbSPL genes identified in this study** | | | | | | | | | | | | | | | | | |
| **Gene Name** | | | **Gene ID** | | | | **Sequence Type** | | | **Sequences** | | | | | | | |
| SbSPL1 | | | evm.model.contig168.565 | | | | Protein | | | MDWNAKWDWESLAAFGSKATEGSKKLQLVDWTVIDDGEIDAGSFNLSTGSRNSGASGSDS GHVSSAKSSITASECSTKEQMQAPDFRLMTLEDFSGNFSKKMELKGAKVSGTFPPPEASL GSVEPLIGLKLGKRTYFENIGAGGNVKSSSISAMPNPSTTSMKKTKPSGQNLPIPRCLVE GCNIDLSAAKEYHRKHRVCDIHSKCPKVIIGGLERRFCQQCSRFHHLSEFDEKKRSCRRR LSDHNARRRKPQQETLQFNSTRLSAPLYGGRQQMSFLLNNSPPLNSRNLANIPWDSTCNS KFTLMKGCPSKSNGDGVSSELHMPGIKLPHLINVQSDSAKGFLASKSSMNEVLDPGSKGS QLSFHLDAAAPEYRRALSLLSSNVWGSCGPESTPYYPPHENNTCVSQPWMMHGIPEGVPL SSSEFLLPPHQSTHPHAPMNSNLPEIQLCITPNETDLYPDILN | | | | | | | |
|  |  |  |  |  |  |  | CDS | | | ATGGATTGGAATGCCAAGTGGGATTGGGAAAGCCTTGCTGCTTTTGGTTCAAAAGCAACAGAAGGTTCTAAGAAGCTGCAATTAGTAGATTGGACTGTTATTGATGATGGAGAAATTGATGCTGGATCCTTCAATCTCTCCACTGGCAGTCGGAATAGTGGAGCCTCTGGCTCTGATAGCGGCCACGTTTCTTCAGCAAAGAGCTCCATAACAGCTTCTGAGTGCTCCACCAAGGAACAGATGCAGGCCCCAGACTTCAGGCTCATGACACTTGAGGATTTTTCTGGAAATTTCAGCAAGAAAATGGAACTAAAAGGAGCGAAGGTTTCTGGAACTTTCCCACCTCCGGAAGCTTCTCTTGGCTCTGTCGAACCACTGATTGGTTTGAAGCTCGGAAAACGAACATATTTTGAGAACATTGGTGCTGGAGGCAATGTTAAGAGTTCATCTATTTCTGCCATGCCTAATCCATCTACCACATCAATGAAGAAAACTAAACCATCAGGTCAAAATCTCCCTATACCACGCTGTCTAGTTGAGGGCTGCAATATTGATCTTTCAGCAGCTAAAGAATATCATCGAAAGCATAGAGTATGCGACATCCATTCCAAATGCCCCAAGGTTATCATAGGTGGCCTTGAACGCAGGTTTTGCCAACAGTGTAGCAGGTTCCATCACCTGTCCGAGTTTGATGAGAAGAAGCGCAGTTGTAGAAGACGACTTTCTGATCATAATGCACGACGTCGAAAGCCACAGCAGGAAACACTCCAGTTTAATTCTACAAGGCTCTCAGCACCATTATATGGAGGAAGGCAACAAATGAGCTTTCTGTTGAACAACAGTCCACCTCTTAACTCTAGAAATCTGGCTAATATTCCTTGGGATAGCACCTGCAACTCCAAGTTCACCCTCATGAAAGGTTGTCCTTCGAAGTCTAATGGAGATGGAGTTAGTAGCGAGCTGCATATGCCGGGAATCAAACTGCCACATCTCATCAATGTGCAAAGTGATTCAGCCAAAGGATTTTTGGCATCCAAGAGCTCCATGAATGAGGTTTTGGATCCAGGTTCAAAGGGATCCCAGTTAAGTTTCCACTTAGATGCAGCAGCACCTGAGTATCGTCGTGCTCTCTCTCTTCTGTCAAGTAATGTATGGGGTTCATGCGGACCAGAATCAACGCCATATTACCCGCCACATGAAAACAATACCTGTGTTAGCCAGCCATGGATGATGCATGGAATCCCTGAAGGTGTGCCCCTTTCTTCTTCAGAATTCTTGCTGCCTCCACATCAATCAACTCATCCTCACGCTCCAATGAACAGTAACCTTCCAGAAATACAGCTTTGCATAACACCTAATGAGACTGACTTATATCCCGACATATTGAACTGA | | | | | | | |
| SbSPL2 | | | evm.model.contig168.577 | | | | Protein | | | MEPSTFSGRHKVCEIHSKTPKVSIRGREQRFCQQCSRFHSLVEFDGEKKSCRKRLDGHNR RRRKTQPDSLTRNSGLDFSNQQGTTLLSFGTPQILPSAVVGISWAGVVKTEHDMGPLCNN QQQLNYIETPNSSCPSQSSYKGVNHLELIQGSDMYSDGLNQVVDSDGALSLLSSASPVTR EFSLNNVLRAEPLPRASSLVNNMQYGGLGHLYQLAQDSKPAVVTSSDSHGTDAGSTLQFG EMFQHGAEGSSSSGSHQTLTFMWE | | | | | | | |
|  |  |  |  |  |  |  | CDS | | | ATGGAACCATCAACGTTTTCGGGGCGGCATAAAGTCTGTGAGATTCACTCTAAAACGCCCAAGGTTTCAATTAGGGGCCGAGAGCAGCGATTTTGCCAGCAATGCAGCAGGTTTCATTCACTGGTAGAGTTTGATGGCGAAAAGAAAAGCTGCAGAAAACGACTCGATGGCCACAACAGGAGACGAAGAAAGACTCAGCCCGACTCACTCACCCGAAACTCAGGACTTGATTTCTCCAACCAACAAGGTACAACACTTCTATCATTTGGAACACCCCAAATCCTACCAAGTGCAGTTGTGGGAATTTCTTGGGCCGGGGTGGTCAAAACCGAGCATGACATGGGCCCGCTATGCAACAATCAGCAGCAGCTCAACTACATCGAGACACCAAACAGCTCGTGCCCGTCCCAGAGTAGCTACAAGGGAGTAAACCATCTCGAGTTAATACAAGGCAGTGATATGTATTCGGATGGGTTAAATCAAGTCGTTGACTCTGATGGTGCTCTCTCTCTTCTGTCATCGGCTAGCCCTGTAACAAGGGAGTTCAGTTTGAACAACGTGCTTCGGGCCGAGCCCCTTCCTCGGGCCTCGTCCTTGGTTAACAACATGCAGTATGGGGGATTAGGGCATCTTTATCAGTTGGCTCAGGATAGCAAGCCAGCTGTTGTTACTTCATCGGACTCACATGGCACTGATGCTGGTTCGACCCTGCAGTTCGGTGAGATGTTTCAGCACGGAGCCGAGGGGTCTTCGTCGAGTGGATCTCATCAAACTCTTACATTCATGTGGGAGTAG | | | | | | | |
| SbSPL3 | | | evm.model.contig539.9 | | | | Protein | | | MDNRSAHSSTGLEWENLAVYNAKHPGDQIHSQFGMNELGSADHQSSCFEGFAKVHHPYSK NSYDPIIGLRLETQREYGHHTSFLLPSTKTDLPVSSMPAKRSRASYQNMQNPCCQVEGCN LDLKSAKEYHRRHRICENHSKSPKVIVAGNERRFCQQCSRFHDLSEFDDKKRSCRRRLSD HNARRRRVQPDSMQLTSTGLISPALYDERKQNVLLSGLPISLTNQTWENVSNCISISNLH MESERFPTIHNSNRRGLDQCLQVASTSNSSIAPDLESTTHSLLSSNVVWSYNQGEPCTSM DGLVSCFRHSEPESNHMSLFQSGHVPMAHSGDLSNGGSSHHQEYQLFKAPYDSGGYYSSQ RPFCVANKSVNILLYACVFDGMQVVVFTLGRRGISE | | | | | | | |
|  |  |  |  |  |  |  | CDS | | | ATGGATAACAGGTCTGCTCACTCTTCCACAGGGTTGGAATGGGAGAATCTAGCAGTCTACAATGCGAAGCATCCTGGTGATCAAATCCACTCCCAATTCGGCATGAACGAGTTGGGATCTGCTGATCATCAGTCAAGCTGCTTCGAGGGTTTTGCTAAGGTGCATCATCCGTACAGCAAGAACTCGTACGACCCAATCATCGGCCTCAGGCTCGAAACACAGAGAGAATATGGGCATCATACCAGCTTTCTGCTTCCTAGTACTAAGACTGATCTTCCTGTTTCCTCCATGCCTGCTAAGAGATCCAGGGCCTCCTACCAGAATATGCAGAATCCTTGTTGCCAAGTTGAGGGCTGCAATCTTGACCTTAAATCTGCTAAAGAGTATCATCGCAGACATCGAATCTGTGAGAACCATTCCAAGAGCCCTAAGGTTATCGTTGCTGGGAATGAACGTCGTTTCTGCCAACAATGCAGCAGGTTTCATGACTTGTCGGAGTTTGATGATAAGAAGCGGAGCTGTAGAAGGCGTCTCTCCGATCACAATGCCAGACGGCGTAGGGTGCAGCCCGATTCTATGCAGCTCACCTCCACTGGATTAATATCTCCTGCTCTATATGATGAGAGAAAGCAGAATGTACTATTGAGTGGACTCCCCATATCTCTAACGAATCAAACATGGGAAAATGTTAGCAATTGTATCTCCATCTCCAATCTACACATGGAGTCGGAGAGATTTCCCACTATCCACAACTCAAACCGTCGAGGTCTTGATCAATGCTTACAAGTAGCAAGCACTAGTAATTCTTCGATTGCTCCGGATCTGGAGAGTACTACTCACTCTCTTCTGTCATCTAACGTCGTCTGGAGCTACAACCAGGGCGAGCCCTGCACCTCGATGGATGGCTTGGTAAGCTGCTTCAGGCATTCTGAACCGGAGTCGAACCACATGTCCTTGTTTCAATCCGGCCATGTTCCGATGGCTCACTCCGGGGATTTGAGCAATGGAGGCTCATCACACCACCAAGAATATCAGTTATTCAAGGCCCCTTATGATTCTGGTGGTTATTATTCCAGTCAAAGACCATTTTGTGTTGCCAATAAATCCGTCAACATTTTGCTATATGCGTGTGTATTCGATGGTATGCAAGTTGTTGTGTTCACGTTGGGTAGGAGAGGAATATCCGAGTGA | | | | | | | |
| SbSPL4 | | | evm.model.contig273.92 | | | | Protein | | | MEKGLSSSSSSSSSSSDSLNGLKFGKKIYFEGVASGFEQPEAAPPQSPAKKGRTAVAQPP RCQVEGCRVDLSGAKAYYSRHKVCGMHSKFPKVIVDGLDQRFHQLPEFDQGKRSCRRRLA GHNERRRKPPQGPLLSSRYASFSPSTFDAWPNASERGSTKQTTITSKYQVPWQSHSQAPQ GSMAQAGYPGPGLSSRDCFSGVSNSTTALSLLSNTNNFLATNGATIGEPSLNPSASIGQF PGSSWDLKGNQANSSLYEMPPDMGLAQISHPSAQPDDDEQCNELELSGGYDSSVQHMNWS L | | | | | | | |
|  |  |  |  |  |  |  | CDS | | | ATGGAAAAGGGTCTATCATCTTCCTCCTCCTCTTCTTCCTCTTCTTCAGACTCACTCAATGGCTTGAAGTTTGGCAAGAAAATCTACTTCGAAGGTGTGGCTTCTGGATTCGAGCAGCCGGAGGCGGCGCCACCGCAGTCCCCGGCCAAGAAGGGGAGGACTGCGGTGGCTCAGCCGCCTCGGTGCCAGGTGGAGGGATGCAGGGTAGATCTGAGTGGTGCTAAGGCTTACTATTCTAGGCACAAAGTTTGTGGTATGCATTCCAAGTTTCCTAAAGTCATTGTTGATGGCCTTGATCAAAGATTTCATCAACTGCCTGAATTCGACCAAGGAAAACGGAGCTGTCGCAGGCGTCTAGCAGGCCACAACGAGCGTCGGAGAAAGCCACCTCAAGGCCCGTTATTGTCTTCACGCTACGCAAGCTTTTCTCCATCCACATTTGACGCATGGCCAAATGCATCCGAACGAGGATCAACCAAACAAACGACTATAACCAGTAAGTACCAAGTTCCATGGCAGAGCCACTCACAAGCTCCACAAGGCTCAATGGCCCAGGCCGGCTATCCGGGCCCGGGATTATCATCAAGAGATTGTTTCAGTGGAGTCTCCAACTCCACCACTGCTCTCTCTCTTCTGTCAAATACTAATAACTTTCTGGCCACCAATGGCGCAACCATTGGTGAGCCATCGCTCAACCCTAGTGCTTCCATTGGTCAATTCCCCGGCTCCTCTTGGGATCTTAAAGGCAATCAAGCTAATAGCTCTCTCTATGAGATGCCTCCTGATATGGGATTAGCACAGATTTCTCACCCTTCTGCTCAACCAGACGATGACGAGCAATGCAACGAGCTCGAGCTGTCTGGGGGCTATGATTCTTCGGTCCAGCATATGAACTGGTCGCTCTGA | | | | | | | |
| SbSPL5 | | | evm.model.contig370.303 | | | | Protein | | | MESLSYACEGRASLFTDDTEFQVDSLVRNRNMMKKWDGEDAVESMGFMEPLFPNTVKNSS RSNQFLEISGDGTDDHSCKSVQSASTITLNSLMEFGKRFSGYVNKANDEKFATPGQSNEN PLSMDHSALSSPEPSISAKRQRITNLQSTIPTCQVYGCNKDLSSSKDYHKRHKVCDAHSK TPVVIVNGIQQRFCQQCSRFHILAEFDEGKRSCRKRLAGHNKRRRKPQFDTHLGSTYFTD ASKTSLIFSRILPSGVFGLQYNEPTNHSRILKLEEEPSQISVLDFHGVEKQFPSKNCLNP LLSLAGSDSSSALSLLSAQSQNLSSNSEGSISMAPSLFTLQNSATLLGETVGYDNPETAF SQESNNLMLKSCLSPEGTNTVDLLELSLHLQRVEQQKYFGQVKLENGVFCDSMIARK | | | | | | | |
|  |  |  |  |  |  |  | CDS | | | ATGGAGTCTTTAAGCTATGCCTGTGAGGGTAGAGCTTCGCTGTTTACTGATGATACAGAATTCCAAGTTGATAGCCTCGTGAGAAATAGAAATATGATGAAGAAATGGGATGGGGAAGATGCAGTTGAGAGTATGGGATTTATGGAGCCTCTTTTTCCTAATACGGTGAAAAACTCGTCTCGTAGTAATCAATTCTTGGAAATCTCTGGTGATGGCACAGATGATCATTCATGTAAATCGGTACAATCTGCTTCTACTATCACATTAAATTCATTAATGGAATTTGGCAAAAGATTTTCCGGTTATGTGAATAAAGCCAACGATGAGAAGTTTGCTACTCCGGGACAATCCAATGAGAATCCATTATCTATGGACCACTCAGCATTGTCTTCTCCAGAGCCCTCCATATCAGCAAAAAGACAGCGAATTACGAATTTGCAGTCAACAATTCCTACTTGCCAAGTTTATGGATGCAACAAAGACTTGAGTTCGTCAAAGGATTACCACAAAAGGCACAAAGTATGCGATGCTCATTCAAAAACTCCTGTGGTTATTGTTAATGGCATCCAGCAAAGGTTTTGTCAGCAATGCAGCAGGTTTCACATACTAGCTGAATTTGACGAAGGCAAGCGTAGTTGTCGTAAACGCCTCGCTGGCCACAATAAGCGTCGTCGAAAACCTCAATTCGACACTCACTTGGGATCCACATATTTCACAGATGCATCAAAAACATCCTTGATTTTCTCAAGAATTCTTCCTAGTGGTGTCTTCGGTCTGCAATATAACGAGCCGACAAACCATAGCAGAATCCTTAAGTTGGAAGAGGAGCCAAGTCAAATATCAGTCCTCGATTTCCATGGTGTTGAAAAACAGTTTCCTTCCAAGAACTGTCTCAACCCTCTCTTATCCCTTGCAGGCTCGGATTCAAGTAGTGCTCTCTCTCTTCTGTCAGCTCAATCACAAAACTTATCAAGCAACTCAGAAGGCAGCATTTCAATGGCTCCTAGCCTCTTTACCCTTCAGAACTCCGCAACGCTTTTGGGAGAAACTGTCGGTTATGACAATCCAGAAACAGCATTTTCACAAGAATCAAACAATCTGATGCTTAAGAGCTGTCTCTCTCCTGAAGGTACGAACACTGTCGATTTGCTTGAGTTGTCATTGCATCTACAAAGAGTAGAACAGCAGAAATACTTTGGCCAGGTCAAGCTCGAAAACGGGGTCTTTTGTGACTCCATGATTGCTCGAAAATGA | | | | | | | |
| SbSPL6 | | | evm.model.contig269.130 | | | | Protein | | | MEKGSSSSSSSSSSPGGPYSLNGLQFGQKIYFKAGTHSKPGGAPPPEEAPPPSPAKKGRT GLLQGGQPPRCQVEGCKLDLSDAKAYYSRHKVCGMHSKSPKVIVAGLEQRFCQQCSRFHQ LPEFDQGKRSCRRRLAGHNERRRKPPPGSLLSPCYGSICPTVFGNHSKTGGYLMDFSSYP TVNGRDPWPDTTAMVQLPSSNPSATIGQFVCPSWDFKDNQGNSTLHEMPPDLGLGQSSHP TNSHYTGELGLAQPNEGQFDQLDHHSRGYDSSVPHLHWSL | | | | | | | |
|  |  |  |  |  |  |  | CDS | | | ATGGAAAAGGGCTCATCCTCATCCTCATCCTCCTCCTCCTCACCTGGCGGGCCCTACTCACTCAATGGCTTGCAATTTGGCCAGAAAATCTACTTTAAGGCTGGAACCCACTCCAAGCCTGGCGGCGCTCCCCCCCCGGAGGAGGCGCCGCCCCCTTCTCCGGCCAAGAAAGGCAGGACAGGGCTCCTGCAGGGTGGCCAGCCGCCTAGGTGCCAGGTGGAGGGCTGCAAACTAGATCTGAGTGATGCTAAGGCTTACTATTCTAGGCACAAAGTTTGTGGTATGCATTCCAAGTCTCCCAAGGTCATTGTTGCTGGCCTTGAGCAAAGATTTTGCCAGCAGTGCAGCAGATTTCATCAATTACCTGAATTCGACCAAGGAAAACGAAGTTGCCGCAGGCGCCTTGCTGGCCACAACGAGCGTAGGAGGAAGCCGCCGCCTGGATCTCTGTTGTCTCCTTGCTATGGAAGTATTTGTCCCACCGTGTTTGGTAACCATAGCAAAACCGGAGGCTATCTGATGGACTTCAGCTCTTACCCAACTGTCAACGGAAGAGATCCATGGCCGGATACCACAGCCATGGTTCAACTACCATCGAGCAATCCTAGTGCAACCATTGGTCAATTTGTCTGCCCTTCCTGGGATTTCAAGGACAACCAAGGTAATAGCACACTGCATGAGATGCCTCCCGATCTTGGCCTTGGACAATCGTCTCACCCTACCAACAGTCACTACACTGGAGAGCTCGGTTTAGCTCAACCAAACGAGGGACAATTCGACCAGCTTGATCATCATTCAAGGGGTTATGATTCTTCAGTCCCGCATCTGCATTGGTCCCTTTAA | | | | | | | |
| SbSPL7 | | | evm.model.contig464.49 | | | | Protein | | | MQQNPSPSIPNPVNDVTSPPIPSDVPTPSSLFDWSEFLDFNLDESLSISFPQPEPEPDPN QHPESSGSENPGRLRKRDPRLVCSNFLAGRIPCACPELDEKLEEEELGLPGKKRTRLVRT PGGAPAKCQVAGCEVDISELKGYHRRHRVCLQCATASAVVIDGESKRYCQQCGKFHILSD FDHDKRSCRRKLERHNNRRRRKPSDSKEGMEKESQQINLVDDLSGDDDTGKGGISVSSQI EEREIQLESDAKVSTLCSSPGSQNLQSNSFKSFTTSGEAHIQGDKQNQKLKKSPSYGDNK SSFSSACPAGRISFKLYDWNPAEFPRRLRLQIFQWLASMPVELEGYIRPGCTILTAFIAL PKPLWHKLLEEPALGIKDLVVSPGSLLSGRCTMHVYLNDMIFRVTKDATSVLKVKVKDQA PKLHYIYPNCFEAGRPMEFVACGSYLLQPNFRFLISFAGRYLEYNISVSSSCREKGGVNS ADHQLLKIYVPQVDRSLSGPAFIEVENQSGLSNFIPILVGDIKTCAEMEVLQQKSSQEQE SSPPHAACEVFASKQVEMSELILDVAWLLKKPLSTQQLTSSHVQRFNYLLEYLIEKNSSI ILEKLFSSLRSAIDNDLAS | | | | | | | |
|  |  |  |  |  |  |  | CDS | | | ATGCAGCAAAACCCATCTCCCTCAATCCCCAATCCAGTCAACGACGTCACAAGTCCCCCAATTCCCTCCGATGTCCCCACCCCTTCATCCCTCTTCGATTGGTCTGAGTTTCTCGACTTCAACCTCGATGAAAGCCTCAGCATATCCTTCCCCCAGCCCGAGCCCGAGCCTGACCCGAACCAGCACCCCGAGAGTTCGGGCTCGGAGAACCCTGGTCGGCTGAGGAAAAGAGACCCCAGATTGGTGTGCTCGAATTTCTTGGCGGGTCGGATCCCGTGTGCCTGCCCTGAATTGGATGAGAAGCTGGAGGAGGAGGAGCTGGGCCTCCCGGGTAAGAAGCGGACGCGGTTGGTGAGGACTCCCGGAGGGGCTCCGGCGAAGTGCCAGGTGGCGGGATGCGAGGTTGACATTAGTGAGCTTAAAGGGTATCATCGGCGCCACCGAGTTTGTTTGCAGTGTGCAACTGCGAGTGCTGTTGTTATTGATGGGGAGAGCAAGAGATACTGTCAGCAGTGTGGAAAGTTCCATATCTTGTCAGACTTTGATCACGATAAACGTAGTTGTCGGCGGAAATTAGAACGCCACAATAACAGACGAAGAAGAAAACCAAGTGATTCTAAAGAAGGCATGGAAAAGGAATCGCAACAGATTAACTTGGTTGATGATCTTAGTGGTGATGATGATACTGGAAAAGGTGGAATATCTGTGAGCAGTCAGATTGAAGAAAGGGAGATTCAGTTGGAGTCAGATGCAAAGGTCTCTACCCTATGCTCATCTCCTGGATCTCAGAATCTTCAAAGCAATAGTTTCAAATCTTTTACCACCTCTGGTGAAGCTCACATCCAAGGGGATAAACAGAATCAAAAACTCAAAAAGTCCCCATCCTATGGTGACAATAAGAGTAGCTTTTCCTCTGCGTGTCCTGCCGGTCGGATCTCTTTTAAGCTATACGATTGGAATCCAGCGGAGTTTCCTCGACGACTTCGACTTCAAATATTCCAATGGTTGGCTAGCATGCCCGTCGAGTTGGAGGGGTATATTCGTCCTGGTTGTACAATTTTGACAGCTTTTATTGCACTGCCAAAGCCACTGTGGCATAAGTTATTGGAGGAACCAGCTCTAGGCATTAAAGATCTTGTTGTCTCGCCAGGGAGCCTGTTGTCTGGAAGATGCACAATGCATGTCTATCTAAATGACATGATTTTTCGTGTCACAAAAGATGCAACTTCAGTACTGAAAGTCAAAGTAAAAGATCAAGCTCCGAAGCTTCATTACATATACCCAAATTGCTTTGAGGCTGGCAGGCCAATGGAATTTGTGGCCTGTGGAAGCTATCTACTTCAACCAAACTTTCGGTTTCTTATTTCATTTGCTGGACGATACCTAGAATACAATATTAGTGTTTCATCTTCGTGCCGTGAGAAAGGAGGTGTCAACAGTGCTGATCATCAATTGTTGAAGATATATGTCCCTCAAGTTGATAGATCTCTTTCTGGTCCTGCTTTTATTGAGGTTGAGAACCAGTCTGGCTTATCCAACTTCATCCCGATTCTTGTTGGAGACATTAAAACTTGTGCAGAAATGGAGGTCTTGCAACAAAAATCATCTCAAGAACAAGAATCATCTCCTCCACATGCTGCATGTGAGGTTTTTGCTTCAAAACAAGTGGAGATGTCAGAGCTTATTTTAGATGTAGCATGGTTGCTGAAGAAACCTTTATCGACACAGCAGTTGACATCTTCCCATGTACAGCGATTCAACTACTTATTAGAGTATTTGATAGAGAAAAATTCTTCCATCATTCTGGAGAAACTATTCTCCTCATTGAGATCTGCAATAGATAATGATTTGGCCAGCTGA | | | | | | | |
| SbSPL8 | | | evm.model.contig420.12 | | | | Protein | | | MEEVGTQVASPIVIHQTLAQRFGNTHPMAKKRSLPFHSSSFVNQNPSDNWNPKSWEWDSS RFVAKPLQCDGTRVRSGSQIPPDLPRSKEIQSGAPNHTKPDHTGKVDENLRLNLGGGERA VTNSCSTNSVEEPQPLSRPNKRVRSGSPGAANHPMCQVDDCKEDLSTAKDYHRRHKVCEV HSKAGKALVGKQMQRFCQQCSRFHPLSEFDDGKRSCRRRLAGHNRRRRKTQPEDTNLRPS VPGGAHNNINCDVDVINLLAALARPQGRTEDMNGKFSSIPEKDQLVQILSKINSLPLPAI LAAKLPHLKTTSGSISDHAHSENQNQMSGNASSRSTMDLLAGLSATPGVPSDTLEIQSQP STEGSDSEKSKSPFVDHSACLDLQTGSIMEFPSVGGERSSTSCYHSPMEEVDFHVQETPP SLHLQLFSSSPEDSSSRKLPWSGNYLSSNSSNPSQEVSPASSPPLVHDLFPMQASRKTTR DDHLSNSENELAYVKSTISNGCSTSLRLFGGSIQATENGSIQSSPYQAGYTSSSGSDHSP SSLNSDSQDRTGRILFKLFDKDPSHLPGSLRTQIFNWLSNSPTEMESYIRPGCIVLSLYL SMPFFSWDRLEENLLNYVNSLVKDIDVGFWGNGRFLICTDRQMVFHKNGKIRLCKTWGAW GIPELISVSPVAVVAGRETSLLLRGKSLTAPGTKVHCTHADGYSMKEVRASSCQDTTNGE IRLDSFKISATASNMLGRCFIEVENSSRGTSFPVIIADKAICHELRLLEPRINDTADVGD DIPADHRQNVGRSWSREDVLHFLDELGWLFQRKSNSSLFGVPEYRLNRFQFLLIFSIEHD FCMLVKTLLDILLELNLGRKGLVRESMAMLSEIHPLNRAVRRRCRRMVDLLVHYSVVDSD DASKRYIFTPNLPGPGGVTPLHLAACTTSSDDIVDALTNDPQKVGLQSWNSVLDCNGLSP YAYASMRNTTRTMPLENGEVSISIGNEINALHIEGESDKTTTVDSKKPCPRCAVAAYRCN KSYPGSQGLLQRPYIHSMLVIAAVCCLCMRFPKRTSVCGLRFSIRMGEPSAMAQYN | | | | | | | |
|  |  |  |  |  |  |  | CDS | | | ATGGAAGAAGTAGGCACCCAAGTAGCTTCTCCTATTGTCATCCACCAAACACTGGCCCAAAGATTTGGCAATACGCATCCAATGGCCAAAAAACGAAGTCTACCTTTTCACTCTTCGAGTTTTGTTAATCAGAATCCATCTGATAACTGGAATCCTAAATCCTGGGAATGGGATAGTTCGAGGTTTGTTGCCAAACCATTGCAATGTGATGGAACCCGAGTGAGAAGCGGCTCACAGATTCCACCAGACTTGCCAAGGAGCAAGGAAATTCAAAGTGGAGCTCCAAATCATACAAAGCCTGATCATACAGGAAAAGTTGATGAAAATCTTCGACTGAATCTGGGAGGTGGAGAGCGAGCTGTAACCAACAGTTGCAGCACGAATTCAGTAGAAGAGCCACAGCCTTTGTCGAGGCCCAATAAGAGAGTAAGATCTGGATCACCTGGAGCTGCTAACCATCCTATGTGTCAAGTAGATGATTGCAAGGAAGATCTCTCCACTGCCAAGGACTATCATCGGCGGCACAAGGTGTGTGAGGTTCACAGTAAAGCTGGTAAAGCCTTAGTGGGAAAACAGATGCAGAGGTTCTGCCAACAGTGCAGCAGGTTCCACCCCCTGTCTGAGTTTGATGATGGAAAGAGAAGCTGTAGGCGGCGACTTGCTGGACATAACAGGAGAAGGAGGAAAACTCAGCCAGAAGATACTAATCTACGACCTTCAGTTCCTGGTGGCGCTCACAACAATATTAATTGTGACGTGGATGTCATCAATTTACTGGCAGCACTAGCTCGTCCTCAAGGGAGAACTGAGGACATGAATGGCAAATTCTCATCAATACCTGAAAAAGATCAGCTGGTTCAAATTCTTAGTAAAATAAATTCATTGCCATTGCCAGCAATCTTAGCAGCCAAGCTGCCTCATTTGAAAACTACAAGTGGAAGTATTTCTGATCATGCACATTCTGAAAATCAAAACCAGATGAGTGGCAATGCTTCATCCCGATCGACTATGGACTTGCTTGCTGGTCTTTCAGCAACTCCAGGAGTACCTTCTGATACATTAGAGATACAATCTCAACCAAGCACAGAGGGGAGTGACTCCGAGAAAAGTAAGTCGCCTTTTGTTGACCATTCTGCATGTCTCGATTTGCAGACTGGATCTATCATGGAGTTCCCGTCTGTTGGAGGTGAGAGAAGCAGTACAAGTTGTTACCATTCTCCCATGGAAGAAGTGGATTTTCATGTCCAAGAGACACCCCCAAGTCTACATCTACAGCTGTTTAGTTCCTCACCTGAAGATAGTAGTTCAAGAAAATTGCCATGGAGTGGAAACTACCTTTCATCTAACAGTAGTAATCCTTCACAAGAGGTGTCACCTGCTTCTTCACCACCACTTGTACATGATCTGTTTCCAATGCAGGCTTCAAGAAAAACCACAAGGGATGATCATTTGTCCAATAGTGAAAATGAACTTGCATATGTTAAATCGACCATCAGCAATGGGTGCAGTACATCTCTTCGTCTTTTTGGGGGCTCAATTCAAGCTACTGAAAATGGTTCAATTCAAAGTTCTCCATACCAAGCAGGTTATACCTCTTCCTCCGGATCTGATCATTCACCATCTAGCCTGAATTCAGATTCCCAGGATCGTACTGGACGAATACTTTTCAAATTGTTTGACAAGGATCCAAGCCATTTGCCTGGTTCTTTACGTACTCAGATCTTCAATTGGCTTTCTAACAGTCCAACGGAAATGGAAAGCTACATTAGGCCTGGTTGCATAGTTCTGTCCCTGTATTTATCAATGCCATTTTTCTCCTGGGATCGACTAGAAGAAAATCTCCTTAATTATGTGAATTCTTTAGTTAAAGATATTGATGTTGGCTTCTGGGGAAATGGAAGATTTTTGATTTGTACGGATAGACAAATGGTTTTTCATAAAAATGGGAAGATTCGTCTCTGCAAAACATGGGGAGCCTGGGGTATACCAGAATTGATCTCGGTTTCTCCTGTTGCTGTTGTTGCTGGGCGAGAGACCTCTCTTTTATTGAGGGGTAAAAGCTTGACAGCTCCAGGGACCAAGGTACACTGCACGCATGCAGATGGGTACAGTATGAAAGAAGTTCGAGCATCTTCTTGTCAAGACACCACAAATGGTGAAATAAGGTTGGATAGCTTTAAGATCAGTGCAACAGCATCTAACATGCTTGGTCGATGTTTTATTGAGGTTGAAAATAGTTCTAGAGGCACTAGTTTCCCAGTAATTATAGCTGATAAGGCCATCTGCCATGAATTGAGACTCCTAGAGCCTCGGATCAACGACACAGCTGATGTCGGCGATGATATTCCAGCCGATCACCGTCAGAATGTGGGGAGGTCCTGGTCCAGGGAAGATGTCCTGCATTTCTTGGATGAACTTGGATGGTTGTTTCAAAGAAAGAGTAACTCTTCTTTGTTTGGGGTCCCAGAATATAGACTTAACCGGTTCCAATTTCTTCTTATATTTTCTATCGAGCACGACTTTTGTATGCTGGTCAAAACCCTCCTGGACATTCTGCTAGAACTAAACTTGGGTAGAAAAGGATTAGTAAGGGAGTCTATGGCAATGCTATCAGAAATCCACCCCTTGAACAGGGCTGTCAGGAGGAGGTGTCGTAGGATGGTTGATTTGCTTGTTCATTACTCTGTTGTTGACTCTGATGATGCTTCTAAGAGGTACATATTCACACCTAATTTGCCTGGACCCGGTGGTGTCACACCTCTACATTTGGCCGCTTGTACAACATCTTCAGATGATATAGTTGATGCTCTAACAAATGACCCTCAGAAGGTTGGGTTGCAGAGTTGGAACTCAGTGCTTGATTGCAATGGACTGTCTCCATATGCATATGCCTCGATGAGGAACACCACTCGTACAATGCCCTTGGAAAATGGTGAAGTCTCCATATCAATTGGAAATGAGATCAATGCGCTCCACATAGAAGGGGAGAGTGACAAGACGACCACCGTCGACTCCAAAAAACCTTGTCCCAGATGTGCAGTTGCAGCTTATAGATGCAATAAAAGTTATCCAGGTTCTCAAGGGTTGCTTCAGCGCCCTTACATCCATTCCATGCTCGTTATTGCTGCCGTATGTTGTTTGTGTATGCGTTTTCCTAAGAGGACATCCGTATGTGGGCTGCGTTTCTCCATTCGCATGGGAGAACCCTCAGCTATGGCACAATATAATTAG | | | | | | | |
| SbSPL9 | | | evm.model.contig420.14 | | | | Protein | | | MEEAGAQVVAPVVMHQHLAARYCDSYPMAKKRALPFHSSSFLQQNTSDNWNPKSWNWDSA RFVAKPLQYDGVQVGTGEEVQLVLPGRDEAHNNASYARKSAHAGKDVENLVVKLGGGGGS GSRGNNGGVNVVEPQPVSRPNKRVRSGSPGGANYPMCQVDNCNEDLSTAKDYHRRHKVCE VHSKASNTLVGKQPQRFCQQCSRFHPLSEFDEGKRSCRRRLAGHNRRRRKTQPEDTTPQV VLPGIHDKNVSKNDIINLLAVLTRPQGNTVENFPPIPDKDQLIQILSKINSLPLPENLAA KLDESNLKHVSAENQNQMNKKASSTSTRDLLAVLSATPGAPSSDTWEIQSPPSSEGSDSE KSKSLCVDQGACLNMHRGPMIEYQGTSPSIPLQLFSLSPEDYRLTKLPSDRKFPSSGSSY PSEERSPQSSPPVEHNLFPMQTSRGKLKDDGLSNSEGEIAFVEAAMTNGCGTSLQLFGSS IGATENVSIHSSPFRIGYTSSSGSDYSPSSQNSDTQDRTGRIIFKLFDKDPSHLPGSLRT QIFNWLSNSPSEMESFIRPGCVVLSLYLSMPSLAWDQLEQNLLRYVNSLVRDIDVDFWGN GRFLVHMNKQMASHEKGKIRLSKSWRDWVTPELISVSPVAIVGGQETSLLLRGRNLKAPG TRIHCTHATGYNIREVNSSSCQKTEYDEVVLSNIKVNGAASGVLGRCFIEVESSFRGASF PVIIADSTICQELTLLEPEINVTTEVRNSIPTDHIQKTGWPKSREEVLHFFDELGWLFQR KYNSCLFEIPDYRLARFKFLFVFTVERDFCALVKTLLDILLELNLGRKGLEKESLEMLSE IHLLNRAVKRRSRKMVDLLLHYSIIDPADSTEKFIFVPNMDGPGGVTPLHMAASTSSSDD IVDALTSDPHEVGLHSWNSVLDANGLSPYAYAVMRNNCSYNTLVARKLANRRNNQVSVSI EDEIKVEVDKDRKTTFHINQGQKSCSRCATVAAVGYSKRFPGAKGLLQRPYIHSMLLVAA VCVCVCLFLRGHPYVGCVSPFAWENLGYGTI | | | | | | | |
|  |  |  |  |  |  |  | CDS | | | ATGGAGGAGGCAGGCGCCCAAGTAGTTGCTCCTGTGGTCATGCACCAGCATCTGGCCGCAAGATATTGTGATTCATATCCGATGGCTAAGAAACGTGCTCTACCTTTTCATTCATCGAGCTTTCTGCAGCAGAACACTTCTGATAACTGGAATCCCAAGTCCTGGAACTGGGACAGTGCGAGGTTTGTTGCAAAACCATTGCAATATGATGGTGTCCAAGTGGGAACGGGTGAAGAGGTTCAGCTAGTCTTGCCAGGGAGAGACGAAGCACATAATAATGCTTCATATGCTAGAAAGTCTGCTCATGCTGGGAAAGATGTTGAAAATCTTGTAGTGAAACTTGGAGGTGGAGGTGGGAGTGGTAGCAGAGGTAACAATGGCGGTGTTAATGTTGTAGAGCCACAACCTGTGTCTAGGCCCAACAAAAGAGTAAGATCTGGATCACCTGGTGGTGCTAACTATCCCATGTGTCAAGTGGATAATTGTAATGAAGATCTGTCGACTGCCAAGGACTATCACCGGAGGCACAAGGTGTGTGAGGTTCACAGTAAAGCCAGTAACACTTTAGTGGGGAAACAACCACAACGGTTCTGCCAGCAGTGCAGCAGGTTCCACCCTCTTTCAGAATTTGACGAAGGAAAGAGAAGTTGTAGGCGCAGACTTGCTGGGCATAACAGGAGGAGAAGGAAAACTCAGCCTGAAGATACTACTCCACAGGTGGTACTTCCTGGTATCCATGACAAAAATGTCAGTAAAAATGACATCATCAATCTATTGGCTGTGCTAACTCGTCCCCAAGGGAACACTGTGGAAAATTTCCCACCGATACCCGACAAAGATCAGCTCATACAAATTCTGAGTAAAATTAATTCATTGCCGTTGCCAGAAAACTTAGCAGCCAAGCTGGATGAGAGTAATCTAAAACATGTATCCGCTGAAAATCAGAACCAAATGAATAAGAAGGCTTCTTCCACATCAACTAGGGACCTGCTTGCTGTTCTTTCAGCAACTCCAGGGGCTCCCTCTTCGGACACATGGGAAATTCAATCTCCACCAAGCAGCGAAGGAAGTGACTCTGAGAAAAGTAAGTCACTTTGTGTCGACCAAGGTGCATGTCTTAATATGCATAGAGGACCTATGATAGAATATCAGGGGACCTCGCCGAGTATACCTTTGCAGCTGTTTAGTCTCTCACCTGAAGATTATAGGTTGACGAAATTGCCATCAGATAGAAAATTTCCTTCGTCTGGGAGCAGTTATCCATCAGAAGAGAGATCACCCCAATCTTCACCACCTGTTGAACATAATCTCTTCCCAATGCAGACTTCAAGAGGAAAACTGAAGGATGACGGTCTGTCAAATAGTGAAGGTGAAATTGCATTTGTAGAAGCAGCGATGACCAATGGGTGTGGCACATCACTTCAGCTTTTTGGCAGCTCAATTGGAGCTACTGAAAATGTTTCAATTCATAGTTCTCCTTTCCGAATTGGATATACATCTTCTTCTGGGTCTGACTATTCACCATCCAGTCAGAATTCTGATACTCAGGATCGTACCGGTCGTATAATTTTCAAGTTGTTTGACAAGGATCCCAGCCATTTGCCTGGATCACTACGGACTCAGATATTCAATTGGCTTTCTAACAGTCCATCAGAAATGGAAAGTTTCATCAGGCCTGGTTGTGTAGTTCTATCTCTGTATTTGTCAATGCCATCTCTTGCCTGGGATCAACTTGAGCAAAACCTTCTTAGATATGTGAATTCGTTAGTCAGAGATATTGATGTTGACTTTTGGGGTAATGGGAGATTTTTGGTTCATATGAACAAACAAATGGCTTCCCATGAAAAGGGAAAGATTCGTCTCAGCAAATCTTGGAGAGATTGGGTTACACCAGAACTGATCTCTGTCTCACCTGTGGCAATTGTTGGTGGACAAGAGACCTCTCTTCTGTTGAGGGGAAGAAATTTGAAAGCTCCAGGCACCAGGATACATTGCACACATGCAACCGGATATAACATAAGGGAAGTAAATTCATCATCGTGCCAAAAGACTGAATATGATGAGGTTGTCCTGTCTAACATTAAGGTCAATGGGGCAGCATCTGGTGTACTTGGGCGTTGTTTCATTGAGGTTGAAAGTAGTTTTAGAGGGGCTAGCTTCCCAGTAATTATAGCAGATAGCACCATCTGCCAAGAATTGACACTCCTCGAGCCTGAGATAAATGTAACTACTGAAGTACGCAATAGTATTCCAACTGATCATATTCAGAAAACAGGGTGGCCAAAGTCGAGGGAAGAAGTCCTGCATTTTTTTGATGAGCTTGGATGGTTGTTTCAAAGAAAGTACAACTCTTGCTTGTTTGAGATCCCAGACTATAGGCTTGCTCGGTTCAAATTTCTTTTCGTGTTCACTGTTGAGCGTGACTTCTGTGCATTAGTCAAAACCCTCCTGGACATTTTGTTAGAACTAAATTTGGGTAGAAAAGGATTAGAGAAGGAGTCTTTGGAGATGCTATCAGAAATTCACCTCTTGAACAGGGCTGTCAAAAGGAGGTCTCGGAAAATGGTTGATTTGCTACTTCATTACTCCATCATCGATCCTGCTGACTCCACTGAGAAATTTATTTTTGTTCCCAACATGGATGGACCTGGTGGTGTCACACCTTTACATATGGCTGCTAGCACATCGTCTTCAGATGATATTGTTGATGCTCTGACAAGCGATCCACATGAGGTTGGGTTGCACAGTTGGAATTCAGTTCTTGATGCAAATGGGCTTTCTCCGTATGCATATGCCGTGATGAGGAATAATTGTTCATACAACACACTTGTTGCCCGGAAGCTTGCCAATAGGAGAAACAATCAAGTCTCTGTATCAATTGAAGATGAGATTAAGGTGGAGGTAGATAAGGACAGAAAGACTACATTCCACATCAATCAAGGGCAAAAATCTTGTTCGAGATGTGCAACGGTTGCAGCAGTTGGATACAGCAAAAGGTTTCCAGGTGCGAAAGGATTACTTCAGCGTCCATACATCCACTCAATGCTTCTTGTTGCTGCTGTTTGTGTTTGTGTCTGTTTGTTCTTAAGAGGACACCCATACGTCGGTTGTGTTTCCCCATTTGCGTGGGAGAACTTGGGCTACGGCACAATTTAA | | | | | | | |
| SbSPL10 | | | evm.model.contig155.11 | | | | Protein | | | MQHNSPTPAYLPRDPEMAAAAEHVAPADPGSSSLFDWSDFLDFDIDEALVTSFLHSELGP GPGNEQSQPSENLGRVRKRDPRLLCPNFQAGRIPCACPELDKALEEEEAEEPALPGKKRA RVVRASAGGPLRCQIPGCEADISELKGYHKRHRVCLQCAHASSVVLDGEEKRYCQQCGKF HVLSNFDEGKRSCRMKLGRHNDRRRKKSNKSKGGTGREAEQVIFADDVKDDDDTEKDDTS PSSQSEEREVLLESDGHVSTIGSAPGSRNLQGESIASFAGPGEIQNNVQKQSLDSKNSAS YHENKNKFSSVCPAGRISFKLYDWNPAEFPRRLRLQIFEWLASMPIELEGYIRPGCTILT AFIAMPKPMWLKLLHEVILTMDKNVWQHSSNSDDTFHFSPDLTVQVIGEKPASHIKDLVA SPGNMLFGRGTMLVYLNDMIFRVTEDSVVQVRVKDRSPKLHYIYPTCFEAGRPMEFLACG SNLLQPKFRFLVSFAGRYLAYNIYVSSLCCKKGNSNSFDHQLVKISVPQTDMNIFGPAFI EVENQSGLSNFIPILFGDKETCAEMDILQQKFSTPSSQEQQRSPPRPTCEALALRQIQFS GFLLDVAWSLKKPETDHPLTSSHLQRFNYLLDFLIEKESFVILGRVIGSLKAAFDNNLVL GSSDSDMKLLQEKMEIAQSMLDQGLLGKGSAMMLTQDGHVHSRTPHNDSQFDSSATEMDV HETDKDIPGYKDIARVPLINRDVVMNVNVHATSRQSRGIFLTKTSLTSRSLVMAIASVCV CFGVCAALIHPQRVSQIATTIRRCLFDGSSS | | | | | | | |
|  |  |  |  |  |  |  | CDS | | | ATGCAGCACAATTCTCCGACTCCAGCGTACCTACCTAGAGACCCCGAGATGGCCGCCGCCGCCGAACATGTAGCTCCGGCCGACCCCGGATCATCTTCCCTCTTCGACTGGTCCGATTTCCTCGATTTCGACATTGACGAAGCTCTCGTCACCTCCTTTCTCCACTCCGAATTGGGGCCCGGACCGGGAAACGAGCAAAGTCAACCGTCGGAGAATTTGGGCCGAGTGAGGAAGAGGGACCCGAGATTATTGTGCCCTAATTTCCAGGCGGGTCGGATCCCGTGCGCGTGCCCTGAATTGGACAAGGCATTGGAGGAAGAGGAGGCGGAGGAGCCGGCTCTTCCCGGAAAGAAGCGGGCCCGGGTGGTTCGTGCCTCAGCTGGGGGCCCGTTACGGTGCCAGATACCGGGTTGTGAGGCGGATATCTCTGAGCTTAAAGGGTATCACAAACGGCACCGAGTGTGCTTGCAATGTGCCCATGCGAGCTCTGTGGTGCTCGATGGGGAAGAGAAGAGGTACTGCCAGCAATGTGGAAAGTTTCATGTCTTGTCGAATTTTGATGAGGGTAAACGTAGTTGTCGGATGAAATTAGGTAGACACAATGACAGACGTCGAAAAAAATCTAATAAGTCCAAAGGAGGCACTGGAAGGGAAGCTGAACAGGTTATCTTTGCTGATGATGTCAAAGATGATGACGACACAGAAAAAGATGACACAAGTCCGAGTAGTCAGAGTGAAGAAAGGGAAGTATTATTGGAGTCAGATGGACATGTCTCGACTATAGGTTCAGCTCCAGGTTCCCGAAACTTGCAGGGTGAAAGTATAGCATCATTTGCCGGGCCTGGTGAAATTCAGAATAATGTGCAGAAACAGAGTCTAGATTCTAAAAACTCTGCATCTTACCATGAGAACAAGAATAAATTTTCATCTGTGTGCCCTGCTGGTCGAATCTCCTTCAAGCTCTATGACTGGAATCCTGCTGAGTTTCCTCGACGGCTTAGACTGCAAATATTTGAATGGTTAGCTAGCATGCCTATTGAGTTGGAGGGGTATATCCGTCCTGGTTGTACGATATTGACAGCTTTCATTGCAATGCCAAAGCCGATGTGGCTTAAGTTATTGCATGAAGTTATTTTAACTATGGACAAAAATGTCTGGCAACATTCATCAAATAGTGATGATACATTTCACTTTTCACCTGATCTTACTGTGCAAGTTATTGGAGAGAAACCAGCTTCACATATAAAAGACCTTGTTGCTTCGCCTGGAAACATGTTATTTGGAAGAGGCACAATGCTTGTTTACTTGAATGACATGATATTCCGTGTCACAGAAGATTCAGTAGTCCAAGTCAGAGTGAAGGATAGATCTCCAAAGCTTCACTATATCTATCCAACTTGCTTTGAGGCTGGCAGGCCCATGGAATTTTTAGCATGTGGAAGCAACCTACTCCAACCTAAGTTTCGGTTTCTTGTTTCGTTTGCTGGACGCTACCTGGCATACAACATTTATGTTTCATCTCTATGTTGTAAGAAAGGGAATAGCAACAGTTTTGACCATCAACTGGTGAAGATATCTGTTCCTCAGACTGACATGAATATTTTTGGCCCGGCATTTATTGAGGTTGAGAACCAGTCCGGCTTATCCAACTTCATTCCAATTCTTTTTGGTGACAAAGAAACCTGTGCGGAAATGGACATCCTACAACAAAAGTTCAGCACACCATCCTCCCAAGAACAACAACGGTCACCTCCAAGGCCTACATGTGAAGCTCTTGCTTTAAGACAAATACAATTCTCTGGGTTTCTTTTAGATGTAGCATGGTCGTTGAAGAAACCTGAAACTGACCATCCATTGACATCTTCCCACTTACAAAGATTCAACTACTTATTGGACTTTTTGATCGAAAAAGAGTCTTTTGTTATTCTGGGGAGAGTTATTGGCTCCTTGAAAGCTGCATTTGATAACAATTTGGTTCTTGGCTCATCTGATTCTGATATGAAGTTGTTACAAGAAAAAATGGAGATTGCACAAAGCATGCTTGATCAAGGATTATTGGGAAAAGGCTCTGCAATGATGCTTACACAGGATGGACATGTTCACAGTCGAACCCCCCACAATGATAGTCAATTTGATTCTTCAGCTACTGAAATGGATGTTCATGAAACAGACAAGGATATCCCAGGATACAAAGATATTGCGAGGGTTCCTCTTATCAACAGAGATGTGGTTATGAACGTTAATGTCCATGCTACATCACGACAATCTCGTGGTATATTTTTAACGAAAACGTCTTTAACTTCACGTTCACTTGTCATGGCTATTGCGAGTGTGTGTGTATGTTTTGGAGTATGTGCTGCCCTTATCCACCCACAACGGGTCAGCCAGATCGCCACCACCATTCGGAGGTGTTTGTTTGATGGCTCCAGCTCGTAG | | | | | | | |
| SbSPL11 | | | evm.model.contig155.257 | | | | Protein | | | MEWDLNDWRWDGDLFVAAPLNPVPTDCRSRQLFPVGSNTSVNNGASNSSSSGSDDVRLQN ERENRDLEKRRRVAEGELDELEGKSGKKTKVSGAPSSRPVCQVEDCKADLSTAKDYHRRH KVCDVHSKATNCLVGNVMQRFCQQCSRFHVLQEFDEGKRSCRRRLAGHNRRRRKTHPDNV ANTCSQNDERGSDYLLISLLRILSNIHANSSDQPKDQDLLTHLLRNLANHAGTTSERNNA ALLPVSQHLQDVGTSLGTDRKDLATSAGQGATIRAPDLIQKRTQTDKAPGVFVQNASTSQ SPLVFPKNTSNSVKENASDTTIGQKKLNNIDLNYEYDGSQDCFEDLPDAFAQKNLGNVSP AGPLLLYKDSERSSPPQNSGNSGSTSSESPSRSQSRTDRIVFKLFGKDPSDFPLVLRKQI LDWLSSSPTDIESYIRPGCIILTIYLRQVILDTPLPVIKHQSCKISSIKPIAVTVSEGVQ FVVKGFNMSRSTSRLLCTLEGKYLVQQNCADMRGGADSFNEHDDIQSFSFCCVIPNIVGR GFIEVEDNGLSSSFFPFIVAEKDVCSEICTLESIIEAADADTYALERRDQALDFIHEMGW LLHRTRMKIRLCETIGNLESSFPFERFRWLMEFSVDHDWCAVVKRLLTALFDGTVHLGQE SSTIKALVDICLIHRAVGRRCRPMLELLLNYHPSAGGLDQTREEKKPLFRPDAVEPGGLT PLHIAASLDSCDDILDALTDDPGSVGIEAWKSARDKSGLTPHEYACLRGHYSYIHLVQRK LNKGQGQGQGQVVVDIPCESSKKQKYAALESEKKMGTGCRQCDQKLGYLRQRASVRIYRP AMVSMVAIAAVCVCTALLFKSSPQVFCSFHPFRWELLKYGSQ | | | | | | | |
|  |  |  |  |  |  |  | CDS | | | ATGGAATGGGATCTGAATGATTGGAGATGGGATGGGGATCTTTTTGTGGCTGCTCCTTTGAATCCTGTTCCTACAGATTGTAGAAGTAGGCAGCTTTTTCCGGTTGGATCAAATACTTCTGTCAACAATGGGGCTTCCAATAGTTCCTCATCAGGTTCAGATGATGTGAGGCTTCAAAATGAGAGGGAGAACAGGGATTTGGAGAAACGGAGAAGGGTAGCTGAGGGTGAGCTGGATGAATTAGAAGGGAAAAGTGGGAAAAAGACCAAAGTCTCTGGTGCACCCTCAAGCCGTCCTGTCTGCCAAGTAGAGGATTGCAAAGCCGATTTGAGCACTGCGAAAGATTACCACAGGCGGCATAAAGTGTGTGATGTCCATTCTAAAGCTACCAATTGTTTGGTGGGAAATGTAATGCAAAGGTTTTGTCAGCAGTGCAGCAGGTTCCATGTTCTTCAAGAGTTTGATGAAGGGAAGCGGAGTTGTCGCAGGCGTCTAGCAGGCCACAACAGACGGCGAAGGAAAACACATCCTGATAATGTAGCAAATACATGCTCCCAGAATGATGAGCGGGGTAGTGATTACTTACTGATCAGTCTTCTAAGAATACTCTCCAATATACATGCAAATAGCTCTGATCAACCAAAGGATCAGGATTTGCTGACTCATCTTTTAAGAAACCTTGCAAATCATGCTGGTACAACTAGTGAGAGAAACAATGCTGCTTTATTACCTGTATCACAGCACTTGCAGGATGTTGGGACATCTTTGGGGACTGATCGAAAGGACCTTGCTACATCTGCTGGGCAAGGTGCAACCATACGTGCACCAGATTTGATACAGAAGAGAACACAAACAGATAAGGCTCCAGGTGTATTTGTACAAAATGCATCCACTTCACAGTCTCCTTTAGTGTTTCCAAAGAACACAAGCAACTCAGTCAAAGAAAACGCTTCTGATACTACTATAGGACAGAAAAAACTGAATAATATTGACTTAAATTATGAGTATGATGGTTCTCAAGACTGTTTTGAGGATCTTCCTGATGCTTTTGCCCAGAAAAATCTGGGCAACGTATCTCCTGCTGGCCCTTTATTGCTATATAAGGATTCTGAACGGTCCAGCCCTCCTCAGAATAGTGGGAATTCTGGTTCCACATCGAGCGAATCACCATCTAGATCCCAGAGTCGCACAGATCGGATTGTCTTTAAGCTTTTCGGAAAAGATCCAAGTGATTTCCCTCTTGTCCTGCGAAAACAGATTCTTGATTGGCTTTCCAGTAGTCCTACTGACATAGAAAGCTACATTCGGCCTGGTTGCATTATACTGACTATATATTTACGCCAGGTCATCCTAGACACACCCTTGCCTGTGATAAAGCATCAAAGTTGCAAAATATCAAGTATAAAGCCAATTGCTGTTACTGTATCAGAGGGTGTTCAATTTGTAGTCAAAGGATTCAATATGTCTCGTTCCACTTCTAGGTTACTATGCACACTAGAGGGAAAGTATCTGGTTCAGCAAAATTGTGCTGATATGAGGGGAGGAGCTGACTCATTTAACGAGCATGATGATATTCAGTCCTTCAGCTTCTGTTGTGTTATACCAAACATTGTTGGAAGGGGATTTATCGAGGTTGAAGACAATGGACTCAGCAGCAGCTTCTTTCCATTCATTGTGGCAGAGAAGGATGTCTGCTCTGAAATTTGTACTCTAGAAAGCATAATCGAGGCTGCTGATGCTGATACTTATGCACTTGAACGTAGAGATCAAGCTTTGGACTTTATACATGAAATGGGCTGGCTGCTCCATAGAACTCGCATGAAGATTAGGTTATGTGAAACCATAGGCAATCTGGAATCATCATTTCCTTTCGAACGCTTCAGGTGGCTAATGGAGTTCTCTGTTGATCATGACTGGTGTGCTGTAGTCAAAAGGCTTTTAACTGCACTGTTTGATGGCACTGTGCATTTGGGACAAGAAAGTTCTACCATAAAAGCACTGGTTGATATTTGCCTCATTCACCGAGCTGTTGGGAGAAGATGCAGGCCCATGTTGGAGCTTCTGCTAAACTACCATCCAAGTGCAGGTGGTCTGGACCAAACACGAGAGGAAAAGAAGCCGCTGTTCAGGCCTGATGCTGTTGAACCAGGTGGATTGACTCCTCTTCATATAGCAGCCAGCCTAGATAGCTGTGATGACATACTGGATGCATTGACTGACGATCCTGGATCTGTCGGAATTGAAGCGTGGAAAAGTGCTAGAGACAAGAGTGGATTAACACCGCACGAATATGCATGTTTGCGTGGCCATTACTCGTACATCCACCTAGTTCAGAGGAAACTCAATAAGGGTCAGGGTCAGGGTCAGGGTCAGGTGGTTGTCGATATTCCTTGTGAAAGTAGCAAGAAGCAGAAATATGCAGCCCTGGAGAGCGAGAAGAAGATGGGAACAGGGTGCAGGCAGTGCGACCAAAAATTGGGATACTTAAGGCAGAGAGCGTCGGTGAGAATATACAGACCGGCCATGGTTTCCATGGTTGCAATAGCAGCTGTATGTGTCTGTACTGCATTGCTCTTCAAAAGCTCACCACAGGTCTTTTGTTCATTCCATCCATTCAGGTGGGAGCTACTGAAGTATGGATCTCAGTAG | | | | | | | |
| SbSPL12 | | | evm.model.contig106.94 | | | | Protein | | | MAAANMDRSVDHKLTKEKMKKDADSDDEELAGGGPPEDDRRKRLASTMKKASGGGGDSSM KSCQAERCSADLTDAKTYHRRHKVCEVHAKAQVVVVAGIRQRFCQQCSRFHELSEFDETK RSCRRRLAGHNERRRKNPSGNPSNGPMMKDMVCGQVDDRGRIQLTVQENANYKHFHVL | | | | | | | |
|  |  |  |  |  |  |  | CDS | | | ATGGCTGCTGCCAACATGGATAGATCAGTCGATCACAAGCTCACGAAGGAGAAGATGAAGAAAGACGCAGACTCCGACGACGAGGAGCTGGCGGGCGGCGGCCCGCCGGAGGATGACCGGAGGAAGAGGCTGGCCAGCACCATGAAGAAAGCCTCCGGCGGCGGCGGTGACAGCTCAATGAAGAGCTGCCAGGCTGAGAGGTGCTCCGCCGATCTGACCGACGCAAAGACGTACCACCGGCGGCACAAGGTGTGTGAGGTCCATGCCAAGGCTCAGGTGGTGGTGGTCGCCGGAATCCGGCAGAGGTTTTGCCAGCAATGCAGCAGGTTTCATGAGCTTTCGGAATTTGATGAAACAAAAAGAAGCTGTCGGAGGCGTTTAGCTGGGCACAACGAGCGGAGGAGGAAGAACCCTTCCGGCAACCCAAGCAACGGTCCGATGATGAAAGATATGGTGTGTGGTCAAGTTGATGATAGAGGACGGATTCAACTCACTGTTCAAGAAAATGCAAACTACAAACACTTCCACGTTCTATGA | | | | | | | |
| SbSPL13 | | | evm.model.contig126.70 | | | | Protein | | | MEAGIGGEAQLYYGVASTDLRAVGKRNLEWDPSDWRWDGDLFIATPLNRTSSNYQGKQLF PLQTGVSETRASSNTSSSNSDELNQGIVRDTRDSEKKRRANAVENSNLVDENLTLNLGGR GYAVEATTGKKTKLGGATSNRAVCQVEDCGADLSKAKDYHRRHKVCEIHSKASKALIGNQ MQRFCQQCSRFHALQEFDEGKRSCRRRLAGHNKRRRKTQMDNVSNNNPVNDNQTSGYLLM SILKILSSMHCKEAMLYCFFFYLIDSIDVMQFVELIIIMIRSNHRDDQDLLSHLLQGLSG QGSLHWERNLSAHLQESQDLLNNLPSLGNSEVASMLISNGSLGQRPRQEYCTNPGDEMPR KNGDLHTSSQRPGVVLHTQADSQIYVQGGETSGGRNKLNNFDLNDVYVDSDDGMDDIERS TIPQGLGTVSIGCPSWVQQESHQSSPPQTSGNSDSASAQSPSSSSGEAQSHTDRIVFKLF GKEPSDFPIVLRAQIFDWLSHSPSDIESYIRPGCVILTIYLRLPESTWEEVWFCLILWKH NYRIVTQSQVVVNASLPLGTDNCSTILSVKPIAVASSGEAQFVIKGFNLSRPSSRLLCAL EGNYLDADNESTEHVDDEGHVQCLKISCLIPAVMGRGFIEVEDHGLSSSYSPFIVAEEDV CTEIRMLEKEIELIELDNLQQGTGRFGGRNTAMEFIHEMGWLLHKFQLKSQLVHEDPNLD CFPFERFKYLVEFSMDRDWCSVVNKLLDIFFSGIVTGGEQPFLKFAVSEMGLLHRAVRRN SRSLVGMLLRYVPEKVADELSLEYKSLVETDQGYLFRPDIAGPGGLTPLHVAAGRDGSED ILDALTDDPGKVGIEAWKSALDSTGFTPDNYARLRGHYSYIHLVQRKINKNISSGHVVVD ISENLSDSSTNQKTSAEPASTFEIGRSVIKHIPRPCGVCAQNLAYRPGNQTLLYRPAMLS MLAIAAVCVCVALLFKSSPQVLFVFRPFRWEMLEYGSS | | | | | | | |
|  |  |  |  |  |  |  | CDS | | | ATGGAGGCTGGAATTGGAGGCGAAGCTCAGCTGTATTACGGCGTGGCTTCTACAGATTTGAGAGCAGTGGGGAAACGAAACTTGGAGTGGGATCCCAGTGATTGGAGATGGGATGGGGACCTCTTCATAGCTACCCCTCTCAATCGCACTTCCTCGAATTATCAAGGCAAACAGTTGTTTCCTCTCCAGACTGGGGTTTCTGAAACTCGAGCTTCCTCCAATACTTCTTCGTCTAATTCCGATGAGTTGAATCAGGGGATTGTTAGAGATACTAGGGATTCGGAGAAGAAAAGGAGGGCCAATGCAGTGGAGAACAGCAATTTGGTTGATGAGAATCTCACTCTGAACCTCGGTGGGCGAGGATATGCAGTTGAAGCAACTACTGGGAAGAAGACTAAATTGGGAGGAGCTACATCGAATCGAGCTGTTTGCCAGGTTGAGGATTGTGGAGCTGATCTTAGTAAGGCGAAAGATTATCATAGACGACACAAAGTCTGTGAGATTCATTCCAAAGCTAGCAAAGCGCTCATAGGAAATCAAATGCAGCGTTTTTGCCAGCAGTGTAGCAGGTTTCATGCACTTCAAGAATTTGATGAAGGAAAGAGAAGTTGCCGCCGGCGCTTGGCTGGCCATAATAAAAGGAGGAGGAAAACACAAATGGACAATGTTAGCAATAACAATCCTGTCAATGACAACCAGACAAGTGGTTACCTCTTGATGAGCATACTTAAGATACTCTCCAGTATGCATTGTAAGGAAGCAATGCTCTACTGTTTCTTTTTCTATCTAATAGATAGTATTGATGTTATGCAATTTGTAGAACTTATTATTATTATGATTAGGTCAAACCATAGAGATGATCAGGATCTCCTATCTCATCTTCTTCAAGGCCTTAGTGGTCAAGGATCCTTACACTGGGAAAGAAACTTATCTGCCCATCTTCAGGAGTCACAAGATTTGCTCAATAATTTGCCGTCATTGGGAAACTCAGAGGTAGCATCTATGTTGATCTCAAATGGCTCTCTAGGACAGAGACCCAGACAAGAATATTGCACGAATCCTGGTGATGAGATGCCAAGAAAAAATGGGGATTTACATACCTCTTCTCAACGACCAGGTGTCGTGCTACACACCCAAGCAGATTCTCAAATCTATGTTCAAGGCGGGGAGACTTCTGGTGGAAGGAACAAACTGAATAATTTTGATTTGAATGATGTTTATGTTGATTCTGATGATGGAATGGATGACATAGAAAGATCAACAATTCCACAGGGCCTAGGGACTGTTTCTATTGGATGTCCTTCATGGGTGCAACAAGAATCTCATCAGTCAAGTCCTCCACAGACCAGCGGAAATTCGGATTCTGCATCTGCGCAGTCACCATCCAGTTCCAGTGGAGAGGCTCAGAGCCACACAGATCGCATTGTTTTCAAACTATTTGGAAAAGAACCTAGTGATTTCCCCATTGTTTTACGAGCACAGATTTTTGATTGGCTATCTCATAGTCCTTCTGACATAGAGAGCTACATTAGGCCAGGTTGTGTCATTTTGACTATATATCTTCGTCTACCTGAGTCAACTTGGGAGGAGGTATGGTTCTGTCTAATACTATGGAAACATAATTACAGAATTGTGACTCAGAGTCAGGTAGTTGTTAATGCGTCTTTGCCTTTAGGGACTGATAACTGTAGCACAATTCTGAGCGTCAAACCTATTGCTGTGGCATCATCTGGGGAAGCTCAGTTCGTTATCAAAGGATTTAATCTATCACGACCTTCCTCAAGGTTACTCTGTGCACTGGAAGGAAATTATCTGGATGCTGATAACGAGTCAACAGAACATGTTGATGACGAGGGGCATGTTCAATGTCTTAAGATTTCCTGCTTGATCCCTGCCGTTATGGGAAGGGGTTTTATTGAGGTTGAAGATCATGGTCTCAGTAGTAGCTATTCTCCCTTCATAGTTGCTGAGGAAGATGTTTGCACTGAGATTCGTATGCTAGAGAAAGAAATAGAATTGATAGAATTAGATAATTTACAGCAAGGAACAGGGAGATTTGGTGGAAGAAACACAGCCATGGAGTTTATACACGAAATGGGATGGCTTCTCCATAAATTTCAGTTGAAGTCCCAATTAGTGCATGAAGATCCTAACTTGGATTGTTTTCCTTTCGAACGTTTCAAATATCTTGTAGAGTTTTCAATGGATCGTGATTGGTGTTCGGTTGTAAATAAGCTCCTGGATATCTTTTTCTCTGGAATAGTGACTGGTGGAGAACAGCCTTTCTTGAAATTTGCTGTGTCTGAGATGGGTCTGCTCCACCGAGCAGTGCGGAGAAATTCAAGGTCTCTTGTTGGGATGCTATTGAGATATGTCCCTGAAAAAGTAGCAGATGAACTCAGTTTGGAATACAAATCTCTGGTTGAAACTGACCAGGGCTATTTATTTAGACCTGACATAGCCGGACCTGGAGGTTTGACCCCTCTTCATGTAGCAGCTGGTAGGGATGGATCTGAGGACATTTTGGATGCTTTGACTGATGATCCTGGAAAGGTGGGAATTGAAGCATGGAAGAGCGCCCTTGACAGCACAGGATTCACGCCTGACAATTATGCTCGATTGAGAGGACACTACTCTTATATTCACCTTGTTCAGAGAAAAATCAACAAGAATATATCCTCTGGACATGTAGTTGTTGACATATCTGAAAACCTATCAGACAGCAGCACTAACCAGAAGACGAGTGCAGAACCCGCATCTACCTTTGAGATTGGAAGGTCTGTGATAAAACACATTCCTCGACCATGTGGTGTATGTGCTCAGAATCTGGCGTATAGGCCTGGCAATCAAACTCTTCTCTACAGGCCTGCGATGCTCTCGATGTTGGCCATAGCAGCTGTATGTGTTTGTGTGGCACTTCTTTTCAAGAGCTCACCGCAAGTTCTGTTTGTGTTCCGTCCATTTAGGTGGGAGATGTTGGAGTATGGTTCCAGCTAA | | | | | | | |
| SbSPL14 | | | evm.model.contig455.712 | | | | Protein | | | MEASKEGKTIMEVPELDDEGEEDEVGNDINKKEASTPAKRRGSAGGSALRSCQVEDCTAD MAYAKQYHRRHKVCEFHAKASEVLHSGLRQRFCQQCSRFHEVSEFDDAKRSCRRRLAGHN ERRRKSSYDSH | | | | | | | |
|  |  |  |  |  |  |  | CDS | | | ATGGAAGCAAGCAAAGAAGGAAAAACGATCATGGAGGTACCTGAATTGGATGATGAAGGTGAAGAAGATGAAGTTGGCAATGATATCAACAAAAAGGAAGCATCGACTCCCGCCAAACGAAGAGGAAGTGCAGGAGGATCAGCACTTCGTTCTTGTCAGGTAGAAGACTGCACTGCTGATATGGCTTACGCCAAGCAATATCATCGTCGCCATAAAGTTTGTGAATTTCATGCCAAGGCCTCAGAGGTTCTTCATTCTGGGTTAAGGCAACGCTTCTGTCAGCAGTGCAGCAGGTTTCATGAAGTGTCAGAGTTCGATGATGCTAAAAGAAGTTGTCGCAGGCGTTTGGCAGGACACAACGAGCGACGCCGCAAGAGCTCGTATGATTCTCACTAA | | | | | | | |

| **Table S4-cis-element of 14 SbSPL genes** | | | | | |  |
| --- | --- | --- | --- | --- | --- | --- |
| **Gene Name** | **Starting position** | **Matrix score** | **Sequence** | **Cis-element name** | **Function** | **Functional classification** |
| SbSPL1 | 1442 | 7 | GAAAGAA | AAGAA-motif |  |  |
| SbSPL1 | 1402 | 9 | gGTAAAGAAA | AAGAA-motif |  |  |
| SbSPL1 | 1047 | 5 | ACGTG | [ABRE](http://bioinformatics.psb.ugent.be/webtools/plantcare/cgi-bin/show_site_info.htpl?QWhere=ID_of_Site%20like%20%27ACGTG%27&StartAt=0&NbRecs=10) | cis-acting element involved in the abscisic acid responsiveness | Hormone responsiveness |
| SbSPL1 | 228 | 8 | AGAAACAA | [AE-box](http://bioinformatics.psb.ugent.be/webtools/plantcare/cgi-bin/show_site_info.htpl?QWhere=ID_of_Site%20like%20%27AGAAACAA%27&StartAt=0&NbRecs=10) | part of a module for light response | Light responsiveness |
| SbSPL1 | 884 | 8 | AGAAACAA | [AE-box](http://bioinformatics.psb.ugent.be/webtools/plantcare/cgi-bin/show_site_info.htpl?QWhere=ID_of_Site%20like%20%27AGAAACAA%27&StartAt=0&NbRecs=10) | part of a module for light response | Light responsiveness |
| SbSPL1 | 196 | 8 | AGAAACAA | [AE-box](http://bioinformatics.psb.ugent.be/webtools/plantcare/cgi-bin/show_site_info.htpl?QWhere=ID_of_Site%20like%20%27AGAAACAA%27&StartAt=0&NbRecs=10) | part of a module for light response | Light responsiveness |
| SbSPL1 | 623 | 6 | AAACCA | [ARE](http://bioinformatics.psb.ugent.be/webtools/plantcare/cgi-bin/show_site_info.htpl?QWhere=ID_of_Site%20like%20%27AAACCA%27&StartAt=0&NbRecs=10) | cis-acting regulatory element essential for the anaerobic induction | Abioticstress |
| SbSPL1 | 613 | 6 | AAACCA | [ARE](http://bioinformatics.psb.ugent.be/webtools/plantcare/cgi-bin/show_site_info.htpl?QWhere=ID_of_Site%20like%20%27AAACCA%27&StartAt=0&NbRecs=10) | cis-acting regulatory element essential for the anaerobic induction | Abioticstress |
| SbSPL1 | 829 | 6 | AAACCA | [ARE](http://bioinformatics.psb.ugent.be/webtools/plantcare/cgi-bin/show_site_info.htpl?QWhere=ID_of_Site%20like%20%27AAACCA%27&StartAt=0&NbRecs=10) | cis-acting regulatory element essential for the anaerobic induction | Abioticstress |
| SbSPL1 | 508 | 6 | AAACCA | [ARE](http://bioinformatics.psb.ugent.be/webtools/plantcare/cgi-bin/show_site_info.htpl?QWhere=ID_of_Site%20like%20%27AAACCA%27&StartAt=0&NbRecs=10) | cis-acting regulatory element essential for the anaerobic induction | Abioticstress |
| SbSPL1 | 604 | 6 | AAACCA | [ARE](http://bioinformatics.psb.ugent.be/webtools/plantcare/cgi-bin/show_site_info.htpl?QWhere=ID_of_Site%20like%20%27AAACCA%27&StartAt=0&NbRecs=10) | cis-acting regulatory element essential for the anaerobic induction | Abioticstress |
| SbSPL1 | 90 | 6 | AAACCA | [ARE](http://bioinformatics.psb.ugent.be/webtools/plantcare/cgi-bin/show_site_info.htpl?QWhere=ID_of_Site%20like%20%27AAACCA%27&StartAt=0&NbRecs=10) | cis-acting regulatory element essential for the anaerobic induction | Abioticstress |
| SbSPL1 | 1907 | 10 | ATAGAAATCAA | [AT-rich element](http://bioinformatics.psb.ugent.be/webtools/plantcare/cgi-bin/show_site_info.htpl?QWhere=ID_of_Site%20like%20%27ATAGAAATCAA%27&StartAt=0&NbRecs=10) | binding site of AT-rich DNA binding protein (ATBP-1) | Promoter-related |
| SbSPL1 | 59 | 6 | TATATA | [AT~TATA-box](http://bioinformatics.psb.ugent.be/webtools/plantcare/cgi-bin/show_site_info.htpl?QWhere=ID_of_Site%20like%20%27TATATA%27&StartAt=0&NbRecs=10) |  |  |
| SbSPL1 | 1806 | 6 | ATTAAT | [Box 4](http://bioinformatics.psb.ugent.be/webtools/plantcare/cgi-bin/show_site_info.htpl?QWhere=ID_of_Site%20like%20%27ATTAAT%27&StartAt=0&NbRecs=10) | part of a conserved DNA module involved in light responsiveness | Light responsiveness |
| SbSPL1 | 1959 | 4 | CAAT | [CAAT-box](http://bioinformatics.psb.ugent.be/webtools/plantcare/cgi-bin/show_site_info.htpl?QWhere=ID_of_Site%20like%20%27CAAT%27&StartAt=0&NbRecs=10) |  |  |
| SbSPL1 | 1914 | 4 | CAAT | [CAAT-box](http://bioinformatics.psb.ugent.be/webtools/plantcare/cgi-bin/show_site_info.htpl?QWhere=ID_of_Site%20like%20%27CAAT%27&StartAt=0&NbRecs=10) |  |  |
| SbSPL1 | 1582 | 4 | CAAT | [CAAT-box](http://bioinformatics.psb.ugent.be/webtools/plantcare/cgi-bin/show_site_info.htpl?QWhere=ID_of_Site%20like%20%27CAAT%27&StartAt=0&NbRecs=10) |  |  |
| SbSPL1 | 1898 | 4 | CAAT | [CAAT-box](http://bioinformatics.psb.ugent.be/webtools/plantcare/cgi-bin/show_site_info.htpl?QWhere=ID_of_Site%20like%20%27CAAT%27&StartAt=0&NbRecs=10) |  |  |
| SbSPL1 | 643 | 4 | CAAT | [CAAT-box](http://bioinformatics.psb.ugent.be/webtools/plantcare/cgi-bin/show_site_info.htpl?QWhere=ID_of_Site%20like%20%27CAAT%27&StartAt=0&NbRecs=10) |  |  |
| SbSPL1 | 366 | 4 | CAAT | [CAAT-box](http://bioinformatics.psb.ugent.be/webtools/plantcare/cgi-bin/show_site_info.htpl?QWhere=ID_of_Site%20like%20%27CAAT%27&StartAt=0&NbRecs=10) |  |  |
| SbSPL1 | 347 | 4 | CAAT | [CAAT-box](http://bioinformatics.psb.ugent.be/webtools/plantcare/cgi-bin/show_site_info.htpl?QWhere=ID_of_Site%20like%20%27CAAT%27&StartAt=0&NbRecs=10) |  |  |
| SbSPL1 | 932 | 5 | CAAAT | [CAAT-box](http://bioinformatics.psb.ugent.be/webtools/plantcare/cgi-bin/show_site_info.htpl?QWhere=ID_of_Site%20like%20%27CAAAT%27&StartAt=0&NbRecs=10) | common cis-acting element in promoter and enhancer regions |  |
| SbSPL1 | 546 | 5 | CCAAT | [CAAT-box](http://bioinformatics.psb.ugent.be/webtools/plantcare/cgi-bin/show_site_info.htpl?QWhere=ID_of_Site%20like%20%27CCAAT%27&StartAt=0&NbRecs=10) | common cis-acting element in promoter and enhancer regions |  |
| SbSPL1 | 1144 | 4 | CAAT | [CAAT-box](http://bioinformatics.psb.ugent.be/webtools/plantcare/cgi-bin/show_site_info.htpl?QWhere=ID_of_Site%20like%20%27CAAT%27&StartAt=0&NbRecs=10) |  |  |
| SbSPL1 | 248 | 5 | CAAAT | [CAAT-box](http://bioinformatics.psb.ugent.be/webtools/plantcare/cgi-bin/show_site_info.htpl?QWhere=ID_of_Site%20like%20%27CAAAT%27&StartAt=0&NbRecs=10) | common cis-acting element in promoter and enhancer regions |  |
| SbSPL1 | 1437 | 5 | CAAAT | [CAAT-box](http://bioinformatics.psb.ugent.be/webtools/plantcare/cgi-bin/show_site_info.htpl?QWhere=ID_of_Site%20like%20%27CAAAT%27&StartAt=0&NbRecs=10) | common cis-acting element in promoter and enhancer regions |  |
| SbSPL1 | 547 | 4 | CAAT | [CAAT-box](http://bioinformatics.psb.ugent.be/webtools/plantcare/cgi-bin/show_site_info.htpl?QWhere=ID_of_Site%20like%20%27CAAT%27&StartAt=0&NbRecs=10) |  |  |
| SbSPL1 | 1706 | 5 | CAAAT | [CAAT-box](http://bioinformatics.psb.ugent.be/webtools/plantcare/cgi-bin/show_site_info.htpl?QWhere=ID_of_Site%20like%20%27CAAAT%27&StartAt=0&NbRecs=10) | common cis-acting element in promoter and enhancer regions |  |
| SbSPL1 | 1819 | 4 | CAAT | [CAAT-box](http://bioinformatics.psb.ugent.be/webtools/plantcare/cgi-bin/show_site_info.htpl?QWhere=ID_of_Site%20like%20%27CAAT%27&StartAt=0&NbRecs=10) |  |  |
| SbSPL1 | 1804 | 4 | CAAT | [CAAT-box](http://bioinformatics.psb.ugent.be/webtools/plantcare/cgi-bin/show_site_info.htpl?QWhere=ID_of_Site%20like%20%27CAAT%27&StartAt=0&NbRecs=10) |  |  |
| SbSPL1 | 33 | 5 | CAAAT | [CAAT-box](http://bioinformatics.psb.ugent.be/webtools/plantcare/cgi-bin/show_site_info.htpl?QWhere=ID_of_Site%20like%20%27CAAAT%27&StartAt=0&NbRecs=10) | common cis-acting element in promoter and enhancer regions |  |
| SbSPL1 | 743 | 4 | CAAT | [CAAT-box](http://bioinformatics.psb.ugent.be/webtools/plantcare/cgi-bin/show_site_info.htpl?QWhere=ID_of_Site%20like%20%27CAAT%27&StartAt=0&NbRecs=10) |  |  |
| SbSPL1 | 1377 | 5 | CAAAT | [CAAT-box](http://bioinformatics.psb.ugent.be/webtools/plantcare/cgi-bin/show_site_info.htpl?QWhere=ID_of_Site%20like%20%27CAAAT%27&StartAt=0&NbRecs=10) | common cis-acting element in promoter and enhancer regions |  |
| SbSPL1 | 153 | 4 | CAAT | [CAAT-box](http://bioinformatics.psb.ugent.be/webtools/plantcare/cgi-bin/show_site_info.htpl?QWhere=ID_of_Site%20like%20%27CAAT%27&StartAt=0&NbRecs=10) |  |  |
| SbSPL1 | 1540 | 4 | CAAT | [CAAT-box](http://bioinformatics.psb.ugent.be/webtools/plantcare/cgi-bin/show_site_info.htpl?QWhere=ID_of_Site%20like%20%27CAAT%27&StartAt=0&NbRecs=10) |  |  |
| SbSPL1 | 538 | 5 | CAAAT | [CAAT-box](http://bioinformatics.psb.ugent.be/webtools/plantcare/cgi-bin/show_site_info.htpl?QWhere=ID_of_Site%20like%20%27CAAAT%27&StartAt=0&NbRecs=10) | common cis-acting element in promoter and enhancer regions |  |
| SbSPL1 | 1921 | 5 | CAAAT | [CAAT-box](http://bioinformatics.psb.ugent.be/webtools/plantcare/cgi-bin/show_site_info.htpl?QWhere=ID_of_Site%20like%20%27CAAAT%27&StartAt=0&NbRecs=10) | common cis-acting element in promoter and enhancer regions |  |
| SbSPL1 | 301 | 5 | CAAAT | [CAAT-box](http://bioinformatics.psb.ugent.be/webtools/plantcare/cgi-bin/show_site_info.htpl?QWhere=ID_of_Site%20like%20%27CAAAT%27&StartAt=0&NbRecs=10) | common cis-acting element in promoter and enhancer regions |  |
| SbSPL1 | 1143 | 5 | CCAAT | [CAAT-box](http://bioinformatics.psb.ugent.be/webtools/plantcare/cgi-bin/show_site_info.htpl?QWhere=ID_of_Site%20like%20%27CCAAT%27&StartAt=0&NbRecs=10) | common cis-acting element in promoter and enhancer regions |  |
| SbSPL1 | 642 | 5 | CCAAT | [CAAT-box](http://bioinformatics.psb.ugent.be/webtools/plantcare/cgi-bin/show_site_info.htpl?QWhere=ID_of_Site%20like%20%27CCAAT%27&StartAt=0&NbRecs=10) | common cis-acting element in promoter and enhancer regions |  |
| SbSPL1 | 1042 | 4 | CAAT | [CAAT-box](http://bioinformatics.psb.ugent.be/webtools/plantcare/cgi-bin/show_site_info.htpl?QWhere=ID_of_Site%20like%20%27CAAT%27&StartAt=0&NbRecs=10) |  |  |
| SbSPL1 | 370 | 5 | CAAAT | [CAAT-box](http://bioinformatics.psb.ugent.be/webtools/plantcare/cgi-bin/show_site_info.htpl?QWhere=ID_of_Site%20like%20%27CAAAT%27&StartAt=0&NbRecs=10) | common cis-acting element in promoter and enhancer regions |  |
| SbSPL1 | 1789 | 4 | CAAT | [CAAT-box](http://bioinformatics.psb.ugent.be/webtools/plantcare/cgi-bin/show_site_info.htpl?QWhere=ID_of_Site%20like%20%27CAAT%27&StartAt=0&NbRecs=10) |  |  |
| SbSPL1 | 619 | 4 | CAAT | [CAAT-box](http://bioinformatics.psb.ugent.be/webtools/plantcare/cgi-bin/show_site_info.htpl?QWhere=ID_of_Site%20like%20%27CAAT%27&StartAt=0&NbRecs=10) |  |  |
| SbSPL1 | 1633 | 4 | CAAT | [CAAT-box](http://bioinformatics.psb.ugent.be/webtools/plantcare/cgi-bin/show_site_info.htpl?QWhere=ID_of_Site%20like%20%27CAAT%27&StartAt=0&NbRecs=10) |  |  |
| SbSPL1 | 19 | 5 | CAAAT | [CAAT-box](http://bioinformatics.psb.ugent.be/webtools/plantcare/cgi-bin/show_site_info.htpl?QWhere=ID_of_Site%20like%20%27CAAAT%27&StartAt=0&NbRecs=10) | common cis-acting element in promoter and enhancer regions |  |
| SbSPL1 | 1015 | 5 | CAAAT | [CAAT-box](http://bioinformatics.psb.ugent.be/webtools/plantcare/cgi-bin/show_site_info.htpl?QWhere=ID_of_Site%20like%20%27CAAAT%27&StartAt=0&NbRecs=10) | common cis-acting element in promoter and enhancer regions |  |
| SbSPL1 | 756 | 5 | CAAAT | [CAAT-box](http://bioinformatics.psb.ugent.be/webtools/plantcare/cgi-bin/show_site_info.htpl?QWhere=ID_of_Site%20like%20%27CAAAT%27&StartAt=0&NbRecs=10) | common cis-acting element in promoter and enhancer regions |  |
| SbSPL1 | 483 | 5 | CAAAT | [CAAT-box](http://bioinformatics.psb.ugent.be/webtools/plantcare/cgi-bin/show_site_info.htpl?QWhere=ID_of_Site%20like%20%27CAAAT%27&StartAt=0&NbRecs=10) | common cis-acting element in promoter and enhancer regions |  |
| SbSPL1 | 233 | 4 | CAAT | [CAAT-box](http://bioinformatics.psb.ugent.be/webtools/plantcare/cgi-bin/show_site_info.htpl?QWhere=ID_of_Site%20like%20%27CAAT%27&StartAt=0&NbRecs=10) |  |  |
| SbSPL1 | 9 | 6 | GCCACT | [CAT-box](http://bioinformatics.psb.ugent.be/webtools/plantcare/cgi-bin/show_site_info.htpl?QWhere=ID_of_Site%20like%20%27GCCACT%27&StartAt=0&NbRecs=10) | cis-acting regulatory element related to meristem expression | Development |
| SbSPL1 | 1045 | 5 | CGTCA | [CGTCA-motif](http://bioinformatics.psb.ugent.be/webtools/plantcare/cgi-bin/show_site_info.htpl?QWhere=ID_of_Site%20like%20%27CGTCA%27&StartAt=0&NbRecs=10) | cis-acting regulatory element involved in the MeJA-responsiveness | Hormone responsiveness |
| SbSPL1 | 1046 | 6 | CACGTC | [G-box](http://bioinformatics.psb.ugent.be/webtools/plantcare/cgi-bin/show_site_info.htpl?QWhere=ID_of_Site%20like%20%27CACGTC%27&StartAt=0&NbRecs=10) | cis-acting regulatory element involved in light responsiveness | Light responsiveness |
| SbSPL1 | 315 | 7 | TCTGTTG | [GARE-motif](http://bioinformatics.psb.ugent.be/webtools/plantcare/cgi-bin/show_site_info.htpl?QWhere=ID_of_Site%20like%20%27TCTGTTG%27&StartAt=0&NbRecs=10) | gibberellin-responsive element | Hormone responsiveness |
| SbSPL1 | 409 | 7 | TCTGTTG | [GARE-motif](http://bioinformatics.psb.ugent.be/webtools/plantcare/cgi-bin/show_site_info.htpl?QWhere=ID_of_Site%20like%20%27TCTGTTG%27&StartAt=0&NbRecs=10) | gibberellin-responsive element | Hormone responsiveness |
| SbSPL1 | 186 | 6 | GGTTAA | [GT1-motif](http://bioinformatics.psb.ugent.be/webtools/plantcare/cgi-bin/show_site_info.htpl?QWhere=ID_of_Site%20like%20%27GGTTAA%27&StartAt=0&NbRecs=10) | light responsive element | Light responsiveness |
| SbSPL1 | 1357 | 10.5 | GTAAT(G/C)ATTAC | [HD-Zip 3](http://bioinformatics.psb.ugent.be/webtools/plantcare/cgi-bin/show_site_info.htpl?QWhere=ID_of_Site%20like%20%27GTAAT%28G%2FC%29ATTAC%27&StartAt=0&NbRecs=10) | protein binding site |  |
| SbSPL1 | 655 | 8 | CTTTATCA | [LAMP-element](http://bioinformatics.psb.ugent.be/webtools/plantcare/cgi-bin/show_site_info.htpl?QWhere=ID_of_Site%20like%20%27CTTTATCA%27&StartAt=0&NbRecs=10) | part of a light responsive element | Light responsiveness |
| SbSPL1 | 817 | 6 | CAACCA | [MYB](http://bioinformatics.psb.ugent.be/webtools/plantcare/cgi-bin/show_site_info.htpl?QWhere=ID_of_Site%20like%20%27CAACCA%27&StartAt=0&NbRecs=10) |  |  |
| SbSPL1 | 1904 | 6 | CAACCA | [MYB](http://bioinformatics.psb.ugent.be/webtools/plantcare/cgi-bin/show_site_info.htpl?QWhere=ID_of_Site%20like%20%27CAACCA%27&StartAt=0&NbRecs=10) |  |  |
| SbSPL1 | 700 | 6 | CAACAG | [MYB](http://bioinformatics.psb.ugent.be/webtools/plantcare/cgi-bin/show_site_info.htpl?QWhere=ID_of_Site%20like%20%27CAACAG%27&StartAt=0&NbRecs=10) |  |  |
| SbSPL1 | 315 | 6 | CAACAG | [MYB](http://bioinformatics.psb.ugent.be/webtools/plantcare/cgi-bin/show_site_info.htpl?QWhere=ID_of_Site%20like%20%27CAACAG%27&StartAt=0&NbRecs=10) |  |  |
| SbSPL1 | 409 | 6 | CAACAG | [MYB](http://bioinformatics.psb.ugent.be/webtools/plantcare/cgi-bin/show_site_info.htpl?QWhere=ID_of_Site%20like%20%27CAACAG%27&StartAt=0&NbRecs=10) |  |  |
| SbSPL1 | 1342 | 6 | TAACCA | [MYB](http://bioinformatics.psb.ugent.be/webtools/plantcare/cgi-bin/show_site_info.htpl?QWhere=ID_of_Site%20like%20%27TAACCA%27&StartAt=0&NbRecs=10) |  |  |
| SbSPL1 | 586 | 6 | TAACCA | [MYB](http://bioinformatics.psb.ugent.be/webtools/plantcare/cgi-bin/show_site_info.htpl?QWhere=ID_of_Site%20like%20%27TAACCA%27&StartAt=0&NbRecs=10) |  |  |
| SbSPL1 | 586 | 6 | TAACCA | [MYB-like sequence](http://bioinformatics.psb.ugent.be/webtools/plantcare/cgi-bin/show_site_info.htpl?QWhere=ID_of_Site%20like%20%27TAACCA%27&StartAt=0&NbRecs=10) |  |  |
| SbSPL1 | 1342 | 6 | TAACCA | [MYB-like sequence](http://bioinformatics.psb.ugent.be/webtools/plantcare/cgi-bin/show_site_info.htpl?QWhere=ID_of_Site%20like%20%27TAACCA%27&StartAt=0&NbRecs=10) |  |  |
| SbSPL1 | 1437 | 6 | CATTTG | [MYC](http://bioinformatics.psb.ugent.be/webtools/plantcare/cgi-bin/show_site_info.htpl?QWhere=ID_of_Site%20like%20%27CATTTG%27&StartAt=0&NbRecs=10) |  |  |
| SbSPL1 | 1745 | 6 | CATGTG | [MYC](http://bioinformatics.psb.ugent.be/webtools/plantcare/cgi-bin/show_site_info.htpl?QWhere=ID_of_Site%20like%20%27CATGTG%27&StartAt=0&NbRecs=10) |  |  |
| SbSPL1 | 301 | 6 | CATTTG | [MYC](http://bioinformatics.psb.ugent.be/webtools/plantcare/cgi-bin/show_site_info.htpl?QWhere=ID_of_Site%20like%20%27CATTTG%27&StartAt=0&NbRecs=10) |  |  |
| SbSPL1 | 19 | 6 | CATTTG | [MYC](http://bioinformatics.psb.ugent.be/webtools/plantcare/cgi-bin/show_site_info.htpl?QWhere=ID_of_Site%20like%20%27CATTTG%27&StartAt=0&NbRecs=10) |  |  |
| SbSPL1 | 538 | 6 | CATTTG | [MYC](http://bioinformatics.psb.ugent.be/webtools/plantcare/cgi-bin/show_site_info.htpl?QWhere=ID_of_Site%20like%20%27CATTTG%27&StartAt=0&NbRecs=10) |  |  |
| SbSPL1 | 927 | 6 | TAACTG | [Myb](http://bioinformatics.psb.ugent.be/webtools/plantcare/cgi-bin/show_site_info.htpl?QWhere=ID_of_Site%20like%20%27TAACTG%27&StartAt=0&NbRecs=10) |  |  |
| SbSPL1 | 700 | 6 | CAACAG | [Myb-binding site](http://bioinformatics.psb.ugent.be/webtools/plantcare/cgi-bin/show_site_info.htpl?QWhere=ID_of_Site%20like%20%27CAACAG%27&StartAt=0&NbRecs=10) |  |  |
| SbSPL1 | 409 | 6 | CAACAG | [Myb-binding site](http://bioinformatics.psb.ugent.be/webtools/plantcare/cgi-bin/show_site_info.htpl?QWhere=ID_of_Site%20like%20%27CAACAG%27&StartAt=0&NbRecs=10) |  |  |
| SbSPL1 | 315 | 6 | CAACAG | [Myb-binding site](http://bioinformatics.psb.ugent.be/webtools/plantcare/cgi-bin/show_site_info.htpl?QWhere=ID_of_Site%20like%20%27CAACAG%27&StartAt=0&NbRecs=10) |  |  |
| SbSPL1 | 1522 | 7 | CCTTTTG | [P-box](http://bioinformatics.psb.ugent.be/webtools/plantcare/cgi-bin/show_site_info.htpl?QWhere=ID_of_Site%20like%20%27CCTTTTG%27&StartAt=0&NbRecs=10) | gibberellin-responsive element | Hormone responsiveness |
| SbSPL1 | 1501 | 5 | AGGGG | [STRE](http://bioinformatics.psb.ugent.be/webtools/plantcare/cgi-bin/show_site_info.htpl?QWhere=ID_of_Site%20like%20%27AGGGG%27&StartAt=0&NbRecs=10) |  |  |
| SbSPL1 | 283 | 8 | TATAAAAT | [TATA](http://bioinformatics.psb.ugent.be/webtools/plantcare/cgi-bin/show_site_info.htpl?QWhere=ID_of_Site%20like%20%27TATAAAAT%27&StartAt=0&NbRecs=10) |  |  |
| SbSPL1 | 1599 | 8 | TATAAAAT | [TATA](http://bioinformatics.psb.ugent.be/webtools/plantcare/cgi-bin/show_site_info.htpl?QWhere=ID_of_Site%20like%20%27TATAAAAT%27&StartAt=0&NbRecs=10) |  |  |
| SbSPL1 | 1970 | 4 | TATA | [TATA-box](http://bioinformatics.psb.ugent.be/webtools/plantcare/cgi-bin/show_site_info.htpl?QWhere=ID_of_Site%20like%20%27TATA%27&StartAt=0&NbRecs=10) | core promoter element around -30 of transcription start |  |
| SbSPL1 | 1942 | 4 | TATA | [TATA-box](http://bioinformatics.psb.ugent.be/webtools/plantcare/cgi-bin/show_site_info.htpl?QWhere=ID_of_Site%20like%20%27TATA%27&StartAt=0&NbRecs=10) | core promoter element around -30 of transcription start |  |
| SbSPL1 | 1727 | 4 | TATA | [TATA-box](http://bioinformatics.psb.ugent.be/webtools/plantcare/cgi-bin/show_site_info.htpl?QWhere=ID_of_Site%20like%20%27TATA%27&StartAt=0&NbRecs=10) | core promoter element around -30 of transcription start |  |
| SbSPL1 | 1600 | 7 | TATAAAA | [TATA-box](http://bioinformatics.psb.ugent.be/webtools/plantcare/cgi-bin/show_site_info.htpl?QWhere=ID_of_Site%20like%20%27TATAAAA%27&StartAt=0&NbRecs=10) | core promoter element around -30 of transcription start |  |
| SbSPL1 | 589 | 5 | TATAA | [TATA-box](http://bioinformatics.psb.ugent.be/webtools/plantcare/cgi-bin/show_site_info.htpl?QWhere=ID_of_Site%20like%20%27TATAA%27&StartAt=0&NbRecs=10) | core promoter element around -30 of transcription start |  |
| SbSPL1 | 251 | 6 | ATATAA | [TATA-box](http://bioinformatics.psb.ugent.be/webtools/plantcare/cgi-bin/show_site_info.htpl?QWhere=ID_of_Site%20like%20%27ATATAA%27&StartAt=0&NbRecs=10) | core promoter element around -30 of transcription start |  |
| SbSPL1 | 116 | 6 | ATTATA | [TATA-box](http://bioinformatics.psb.ugent.be/webtools/plantcare/cgi-bin/show_site_info.htpl?QWhere=ID_of_Site%20like%20%27ATTATA%27&StartAt=0&NbRecs=10) | core promoter element around -30 of transcription start |  |
| SbSPL1 | 102 | 7 | TACAAAA | [TATA-box](http://bioinformatics.psb.ugent.be/webtools/plantcare/cgi-bin/show_site_info.htpl?QWhere=ID_of_Site%20like%20%27TACAAAA%27&StartAt=0&NbRecs=10) | core promoter element around -30 of transcription start |  |
| SbSPL1 | 61 | 4 | TATA | [TATA-box](http://bioinformatics.psb.ugent.be/webtools/plantcare/cgi-bin/show_site_info.htpl?QWhere=ID_of_Site%20like%20%27TATA%27&StartAt=0&NbRecs=10) | core promoter element around -30 of transcription start |  |
| SbSPL1 | 59 | 6 | TATATA | [TATA-box](http://bioinformatics.psb.ugent.be/webtools/plantcare/cgi-bin/show_site_info.htpl?QWhere=ID_of_Site%20like%20%27TATATA%27&StartAt=0&NbRecs=10) | core promoter element around -30 of transcription start |  |
| SbSPL1 | 57 | 6 | ATTATA | [TATA-box](http://bioinformatics.psb.ugent.be/webtools/plantcare/cgi-bin/show_site_info.htpl?QWhere=ID_of_Site%20like%20%27ATTATA%27&StartAt=0&NbRecs=10) | core promoter element around -30 of transcription start |  |
| SbSPL1 | 1948 | 5 | TATAA | [TATA-box](http://bioinformatics.psb.ugent.be/webtools/plantcare/cgi-bin/show_site_info.htpl?QWhere=ID_of_Site%20like%20%27TATAA%27&StartAt=0&NbRecs=10) | core promoter element around -30 of transcription start |  |
| SbSPL1 | 1074 | 9 | ccTATAAAaa | [TATA-box](http://bioinformatics.psb.ugent.be/webtools/plantcare/cgi-bin/show_site_info.htpl?QWhere=ID_of_Site%20like%20%27ccTATAAAaa%27&StartAt=0&NbRecs=10) | core promoter element around -30 of transcription start |  |
| SbSPL1 | 1683 | 4 | TATA | [TATA-box](http://bioinformatics.psb.ugent.be/webtools/plantcare/cgi-bin/show_site_info.htpl?QWhere=ID_of_Site%20like%20%27TATA%27&StartAt=0&NbRecs=10) | core promoter element around -30 of transcription start |  |
| SbSPL1 | 1725 | 6 | ATTATA | [TATA-box](http://bioinformatics.psb.ugent.be/webtools/plantcare/cgi-bin/show_site_info.htpl?QWhere=ID_of_Site%20like%20%27ATTATA%27&StartAt=0&NbRecs=10) | core promoter element around -30 of transcription start |  |
| SbSPL1 | 1691 | 4 | TATA | [TATA-box](http://bioinformatics.psb.ugent.be/webtools/plantcare/cgi-bin/show_site_info.htpl?QWhere=ID_of_Site%20like%20%27TATA%27&StartAt=0&NbRecs=10) | core promoter element around -30 of transcription start |  |
| SbSPL1 | 58 | 7 | TATATAA | [TATA-box](http://bioinformatics.psb.ugent.be/webtools/plantcare/cgi-bin/show_site_info.htpl?QWhere=ID_of_Site%20like%20%27TATATAA%27&StartAt=0&NbRecs=10) | core promoter element around -30 of transcription start |  |
| SbSPL1 | 1603 | 4 | TATA | [TATA-box](http://bioinformatics.psb.ugent.be/webtools/plantcare/cgi-bin/show_site_info.htpl?QWhere=ID_of_Site%20like%20%27TATA%27&StartAt=0&NbRecs=10) | core promoter element around -30 of transcription start |  |
| SbSPL1 | 1949 | 4 | TATA | [TATA-box](http://bioinformatics.psb.ugent.be/webtools/plantcare/cgi-bin/show_site_info.htpl?QWhere=ID_of_Site%20like%20%27TATA%27&StartAt=0&NbRecs=10) | core promoter element around -30 of transcription start |  |
| SbSPL1 | 1688 | 7 | TATAAAA | [TATA-box](http://bioinformatics.psb.ugent.be/webtools/plantcare/cgi-bin/show_site_info.htpl?QWhere=ID_of_Site%20like%20%27TATAAAA%27&StartAt=0&NbRecs=10) | core promoter element around -30 of transcription start |  |
| SbSPL1 | 252 | 4 | TATA | [TATA-box](http://bioinformatics.psb.ugent.be/webtools/plantcare/cgi-bin/show_site_info.htpl?QWhere=ID_of_Site%20like%20%27TATA%27&StartAt=0&NbRecs=10) | core promoter element around -30 of transcription start |  |
| SbSPL1 | 1602 | 5 | TATAA | [TATA-box](http://bioinformatics.psb.ugent.be/webtools/plantcare/cgi-bin/show_site_info.htpl?QWhere=ID_of_Site%20like%20%27TATAA%27&StartAt=0&NbRecs=10) | core promoter element around -30 of transcription start |  |
| SbSPL1 | 1929 | 6 | ATTATA | [TATA-box](http://bioinformatics.psb.ugent.be/webtools/plantcare/cgi-bin/show_site_info.htpl?QWhere=ID_of_Site%20like%20%27ATTATA%27&StartAt=0&NbRecs=10) | core promoter element around -30 of transcription start |  |
| SbSPL1 | 117 | 5 | TATAA | [TATA-box](http://bioinformatics.psb.ugent.be/webtools/plantcare/cgi-bin/show_site_info.htpl?QWhere=ID_of_Site%20like%20%27TATAA%27&StartAt=0&NbRecs=10) | core promoter element around -30 of transcription start |  |
| SbSPL1 | 1925 | 4 | TATA | [TATA-box](http://bioinformatics.psb.ugent.be/webtools/plantcare/cgi-bin/show_site_info.htpl?QWhere=ID_of_Site%20like%20%27TATA%27&StartAt=0&NbRecs=10) | core promoter element around -30 of transcription start |  |
| SbSPL1 | 1924 | 6 | ATATAA | [TATA-box](http://bioinformatics.psb.ugent.be/webtools/plantcare/cgi-bin/show_site_info.htpl?QWhere=ID_of_Site%20like%20%27ATATAA%27&StartAt=0&NbRecs=10) | core promoter element around -30 of transcription start |  |
| SbSPL1 | 711 | 6 | ATTATA | [TATA-box](http://bioinformatics.psb.ugent.be/webtools/plantcare/cgi-bin/show_site_info.htpl?QWhere=ID_of_Site%20like%20%27ATTATA%27&StartAt=0&NbRecs=10) | core promoter element around -30 of transcription start |  |
| SbSPL1 | 118 | 4 | TATA | [TATA-box](http://bioinformatics.psb.ugent.be/webtools/plantcare/cgi-bin/show_site_info.htpl?QWhere=ID_of_Site%20like%20%27TATA%27&StartAt=0&NbRecs=10) | core promoter element around -30 of transcription start |  |
| SbSPL1 | 713 | 4 | TATA | [TATA-box](http://bioinformatics.psb.ugent.be/webtools/plantcare/cgi-bin/show_site_info.htpl?QWhere=ID_of_Site%20like%20%27TATA%27&StartAt=0&NbRecs=10) | core promoter element around -30 of transcription start |  |
| SbSPL1 | 1969 | 6 | ATATAA | [TATA-box](http://bioinformatics.psb.ugent.be/webtools/plantcare/cgi-bin/show_site_info.htpl?QWhere=ID_of_Site%20like%20%27ATATAA%27&StartAt=0&NbRecs=10) | core promoter element around -30 of transcription start |  |
| SbSPL1 | 1601 | 6 | TATAAA | [TATA-box](http://bioinformatics.psb.ugent.be/webtools/plantcare/cgi-bin/show_site_info.htpl?QWhere=ID_of_Site%20like%20%27TATAAA%27&StartAt=0&NbRecs=10) | core promoter element around -30 of transcription start |  |
| SbSPL1 | 1690 | 5 | TATAA | [TATA-box](http://bioinformatics.psb.ugent.be/webtools/plantcare/cgi-bin/show_site_info.htpl?QWhere=ID_of_Site%20like%20%27TATAA%27&StartAt=0&NbRecs=10) | core promoter element around -30 of transcription start |  |
| SbSPL1 | 283 | 4 | TATA | [TATA-box](http://bioinformatics.psb.ugent.be/webtools/plantcare/cgi-bin/show_site_info.htpl?QWhere=ID_of_Site%20like%20%27TATA%27&StartAt=0&NbRecs=10) | core promoter element around -30 of transcription start |  |
| SbSPL1 | 1931 | 4 | TATA | [TATA-box](http://bioinformatics.psb.ugent.be/webtools/plantcare/cgi-bin/show_site_info.htpl?QWhere=ID_of_Site%20like%20%27TATA%27&StartAt=0&NbRecs=10) | core promoter element around -30 of transcription start |  |
| SbSPL1 | 120 | 7 | TACAAAA | [TATA-box](http://bioinformatics.psb.ugent.be/webtools/plantcare/cgi-bin/show_site_info.htpl?QWhere=ID_of_Site%20like%20%27TACAAAA%27&StartAt=0&NbRecs=10) | core promoter element around -30 of transcription start |  |
| SbSPL1 | 590 | 4 | TATA | [TATA-box](http://bioinformatics.psb.ugent.be/webtools/plantcare/cgi-bin/show_site_info.htpl?QWhere=ID_of_Site%20like%20%27TATA%27&StartAt=0&NbRecs=10) | core promoter element around -30 of transcription start |  |
| SbSPL1 | 1689 | 6 | TATAAA | [TATA-box](http://bioinformatics.psb.ugent.be/webtools/plantcare/cgi-bin/show_site_info.htpl?QWhere=ID_of_Site%20like%20%27TATAAA%27&StartAt=0&NbRecs=10) | core promoter element around -30 of transcription start |  |
| SbSPL1 | 712 | 5 | TATAA | [TATA-box](http://bioinformatics.psb.ugent.be/webtools/plantcare/cgi-bin/show_site_info.htpl?QWhere=ID_of_Site%20like%20%27TATAA%27&StartAt=0&NbRecs=10) | core promoter element around -30 of transcription start |  |
| SbSPL1 | 1930 | 5 | TATAA | [TATA-box](http://bioinformatics.psb.ugent.be/webtools/plantcare/cgi-bin/show_site_info.htpl?QWhere=ID_of_Site%20like%20%27TATAA%27&StartAt=0&NbRecs=10) | core promoter element around -30 of transcription start |  |
| SbSPL1 | 1922 | 12 | TATATTTATATTT | [TATA-box](http://bioinformatics.psb.ugent.be/webtools/plantcare/cgi-bin/show_site_info.htpl?QWhere=ID_of_Site%20like%20%27TATATTTATATTT%27&StartAt=0&NbRecs=10) | core promoter element around -30 of transcription start |  |
| SbSPL1 | 1941 | 5 | TATAA | [TATA-box](http://bioinformatics.psb.ugent.be/webtools/plantcare/cgi-bin/show_site_info.htpl?QWhere=ID_of_Site%20like%20%27TATAA%27&StartAt=0&NbRecs=10) | core promoter element around -30 of transcription start |  |
| SbSPL1 | 1726 | 5 | TATAA | [TATA-box](http://bioinformatics.psb.ugent.be/webtools/plantcare/cgi-bin/show_site_info.htpl?QWhere=ID_of_Site%20like%20%27TATAA%27&StartAt=0&NbRecs=10) | core promoter element around -30 of transcription start |  |
| SbSPL1 | 1770 | 4 | TATA | [TATA-box](http://bioinformatics.psb.ugent.be/webtools/plantcare/cgi-bin/show_site_info.htpl?QWhere=ID_of_Site%20like%20%27TATA%27&StartAt=0&NbRecs=10) | core promoter element around -30 of transcription start |  |
| SbSPL1 | 488 | 9 | TCATCTTCAT | [TCA](http://bioinformatics.psb.ugent.be/webtools/plantcare/cgi-bin/show_site_info.htpl?QWhere=ID_of_Site%20like%20%27TCATCTTCAT%27&StartAt=0&NbRecs=10) |  |  |
| SbSPL1 | 1045 | 5 | TGACG | [TGACG-motif](http://bioinformatics.psb.ugent.be/webtools/plantcare/cgi-bin/show_site_info.htpl?QWhere=ID_of_Site%20like%20%27TGACG%27&StartAt=0&NbRecs=10) | cis-acting regulatory element involved in the MeJA-responsiveness | Hormone responsiveness |
| SbSPL1 | 1048 | 5 | CGTGG | [Unnamed__1](http://bioinformatics.psb.ugent.be/webtools/plantcare/cgi-bin/show_site_info.htpl?QWhere=ID_of_Site%20like%20%27CGTGG%27&StartAt=0&NbRecs=10) |  |  |
| SbSPL1 | 1742 | 4 | CTCC | [Unnamed__4](http://bioinformatics.psb.ugent.be/webtools/plantcare/cgi-bin/show_site_info.htpl?QWhere=ID_of_Site%20like%20%27CTCC%27&StartAt=0&NbRecs=10) |  |  |
| SbSPL1 | 1611 | 4 | CTCC | [Unnamed__4](http://bioinformatics.psb.ugent.be/webtools/plantcare/cgi-bin/show_site_info.htpl?QWhere=ID_of_Site%20like%20%27CTCC%27&StartAt=0&NbRecs=10) |  |  |
| SbSPL1 | 1650 | 4 | CTCC | [Unnamed__4](http://bioinformatics.psb.ugent.be/webtools/plantcare/cgi-bin/show_site_info.htpl?QWhere=ID_of_Site%20like%20%27CTCC%27&StartAt=0&NbRecs=10) |  |  |
| SbSPL1 | 1545 | 4 | CTCC | [Unnamed__4](http://bioinformatics.psb.ugent.be/webtools/plantcare/cgi-bin/show_site_info.htpl?QWhere=ID_of_Site%20like%20%27CTCC%27&StartAt=0&NbRecs=10) |  |  |
| SbSPL1 | 580 | 4 | CTCC | [Unnamed__4](http://bioinformatics.psb.ugent.be/webtools/plantcare/cgi-bin/show_site_info.htpl?QWhere=ID_of_Site%20like%20%27CTCC%27&StartAt=0&NbRecs=10) |  |  |
| SbSPL1 | 1527 | 4 | CTCC | [Unnamed__4](http://bioinformatics.psb.ugent.be/webtools/plantcare/cgi-bin/show_site_info.htpl?QWhere=ID_of_Site%20like%20%27CTCC%27&StartAt=0&NbRecs=10) |  |  |
| SbSPL1 | 911 | 4 | CTCC | [Unnamed__4](http://bioinformatics.psb.ugent.be/webtools/plantcare/cgi-bin/show_site_info.htpl?QWhere=ID_of_Site%20like%20%27CTCC%27&StartAt=0&NbRecs=10) |  |  |
| SbSPL1 | 544 | 4 | CTCC | [Unnamed__4](http://bioinformatics.psb.ugent.be/webtools/plantcare/cgi-bin/show_site_info.htpl?QWhere=ID_of_Site%20like%20%27CTCC%27&StartAt=0&NbRecs=10) |  |  |
| SbSPL1 | 442 | 4 | CTCC | [Unnamed__4](http://bioinformatics.psb.ugent.be/webtools/plantcare/cgi-bin/show_site_info.htpl?QWhere=ID_of_Site%20like%20%27CTCC%27&StartAt=0&NbRecs=10) |  |  |
| SbSPL1 | 333 | 4 | CTCC | [Unnamed__4](http://bioinformatics.psb.ugent.be/webtools/plantcare/cgi-bin/show_site_info.htpl?QWhere=ID_of_Site%20like%20%27CTCC%27&StartAt=0&NbRecs=10) |  |  |
| SbSPL1 | 864 | 4 | CTCC | [Unnamed__4](http://bioinformatics.psb.ugent.be/webtools/plantcare/cgi-bin/show_site_info.htpl?QWhere=ID_of_Site%20like%20%27CTCC%27&StartAt=0&NbRecs=10) |  |  |
| SbSPL1 | 849 | 4 | CTCC | [Unnamed__4](http://bioinformatics.psb.ugent.be/webtools/plantcare/cgi-bin/show_site_info.htpl?QWhere=ID_of_Site%20like%20%27CTCC%27&StartAt=0&NbRecs=10) |  |  |
| SbSPL1 | 907 | 6 | CCACCT | [WRE3](http://bioinformatics.psb.ugent.be/webtools/plantcare/cgi-bin/show_site_info.htpl?QWhere=ID_of_Site%20like%20%27CCACCT%27&StartAt=0&NbRecs=10) |  |  |
| SbSPL1 | 1613 | 6 | CCACCT | [WRE3](http://bioinformatics.psb.ugent.be/webtools/plantcare/cgi-bin/show_site_info.htpl?QWhere=ID_of_Site%20like%20%27CCACCT%27&StartAt=0&NbRecs=10) |  |  |
| SbSPL1 | 1045 | 5 | TGACG | [as-1](http://bioinformatics.psb.ugent.be/webtools/plantcare/cgi-bin/show_site_info.htpl?QWhere=ID_of_Site%20like%20%27TGACG%27&StartAt=0&NbRecs=10) |  |  |
| SbSPL1 | 844 | 7 | AGCCACC | [box S](http://bioinformatics.psb.ugent.be/webtools/plantcare/cgi-bin/show_site_info.htpl?QWhere=ID_of_Site%20like%20%27AGCCACC%27&StartAt=0&NbRecs=10) |  |  |
| SbSPL2 | 1827 | 7 | GAAAGAA | [AAGAA-motif](http://bioinformatics.psb.ugent.be/webtools/plantcare/cgi-bin/show_site_info.htpl?QWhere=ID_of_Site%20like%20%27GAAAGAA%27&StartAt=0&NbRecs=10) |  |  |
| SbSPL2 | 1821 | 9 | gGTAAAGAAA | [AAGAA-motif](http://bioinformatics.psb.ugent.be/webtools/plantcare/cgi-bin/show_site_info.htpl?QWhere=ID_of_Site%20like%20%27gGTAAAGAAA%27&StartAt=0&NbRecs=10) |  |  |
| SbSPL2 | 3 | 7 | GAAAGAA | [AAGAA-motif](http://bioinformatics.psb.ugent.be/webtools/plantcare/cgi-bin/show_site_info.htpl?QWhere=ID_of_Site%20like%20%27GAAAGAA%27&StartAt=0&NbRecs=10) |  |  |
| SbSPL2 | 667 | 8.5 | (T/C)C(T/C)(C/T)ACC(T/C)ACC | [AC-I](http://bioinformatics.psb.ugent.be/webtools/plantcare/cgi-bin/show_site_info.htpl?QWhere=ID_of_Site%20like%20%27%28T%2FC%29C%28T%2FC%29%28C%2FT%29ACC%28T%2FC%29ACC%27&StartAt=0&NbRecs=10) |  |  |
| SbSPL2 | 663 | 8.5 | (T/C)C(T/C)(C/T)ACC(T/C)ACC | [AC-I](http://bioinformatics.psb.ugent.be/webtools/plantcare/cgi-bin/show_site_info.htpl?QWhere=ID_of_Site%20like%20%27%28T%2FC%29C%28T%2FC%29%28C%2FT%29ACC%28T%2FC%29ACC%27&StartAt=0&NbRecs=10) |  |  |
| SbSPL2 | 538 | 8.5 | (T/C)C(T/C)(C/T)ACC(T/C)ACC | [AC-I](http://bioinformatics.psb.ugent.be/webtools/plantcare/cgi-bin/show_site_info.htpl?QWhere=ID_of_Site%20like%20%27%28T%2FC%29C%28T%2FC%29%28C%2FT%29ACC%28T%2FC%29ACC%27&StartAt=0&NbRecs=10) |  |  |
| SbSPL2 | 540 | 11 | CCACCAACCCCC | [AC-II](http://bioinformatics.psb.ugent.be/webtools/plantcare/cgi-bin/show_site_info.htpl?QWhere=ID_of_Site%20like%20%27CCACCAACCCCC%27&StartAt=0&NbRecs=10) |  |  |
| SbSPL2 | 842 | 6 | AAACCA | [ARE](http://bioinformatics.psb.ugent.be/webtools/plantcare/cgi-bin/show_site_info.htpl?QWhere=ID_of_Site%20like%20%27AAACCA%27&StartAt=0&NbRecs=10) | cis-acting regulatory element essential for the anaerobic induction | Abioticstress |
| SbSPL2 | 1206 | 6 | AAACCA | [ARE](http://bioinformatics.psb.ugent.be/webtools/plantcare/cgi-bin/show_site_info.htpl?QWhere=ID_of_Site%20like%20%27AAACCA%27&StartAt=0&NbRecs=10) | cis-acting regulatory element essential for the anaerobic induction | Abioticstress |
| SbSPL2 | 449 | 6 | AAACCA | [ARE](http://bioinformatics.psb.ugent.be/webtools/plantcare/cgi-bin/show_site_info.htpl?QWhere=ID_of_Site%20like%20%27AAACCA%27&StartAt=0&NbRecs=10) | cis-acting regulatory element essential for the anaerobic induction | Abioticstress |
| SbSPL2 | 1751 | 10 | ATAGAAATCAA | [AT-rich element](http://bioinformatics.psb.ugent.be/webtools/plantcare/cgi-bin/show_site_info.htpl?QWhere=ID_of_Site%20like%20%27ATAGAAATCAA%27&StartAt=0&NbRecs=10) | binding site of AT-rich DNA binding protein (ATBP-1) | Promoter-related |
| SbSPL2 | 1040 | 9 | TAAAATACT | [AT-rich sequence](http://bioinformatics.psb.ugent.be/webtools/plantcare/cgi-bin/show_site_info.htpl?QWhere=ID_of_Site%20like%20%27TAAAATACT%27&StartAt=0&NbRecs=10) | element for maximal elicitor-mediated activation (2copies) |  |
| SbSPL2 | 1372 | 6 | TATATA | [AT~TATA-box](http://bioinformatics.psb.ugent.be/webtools/plantcare/cgi-bin/show_site_info.htpl?QWhere=ID_of_Site%20like%20%27TATATA%27&StartAt=0&NbRecs=10) |  |  |
| SbSPL2 | 1286 | 6 | TATATA | [AT~TATA-box](http://bioinformatics.psb.ugent.be/webtools/plantcare/cgi-bin/show_site_info.htpl?QWhere=ID_of_Site%20like%20%27TATATA%27&StartAt=0&NbRecs=10) |  |  |
| SbSPL2 | 286 | 6 | TATATA | [AT~TATA-box](http://bioinformatics.psb.ugent.be/webtools/plantcare/cgi-bin/show_site_info.htpl?QWhere=ID_of_Site%20like%20%27TATATA%27&StartAt=0&NbRecs=10) |  |  |
| SbSPL2 | 432 | 6 | TATATA | [AT~TATA-box](http://bioinformatics.psb.ugent.be/webtools/plantcare/cgi-bin/show_site_info.htpl?QWhere=ID_of_Site%20like%20%27TATATA%27&StartAt=0&NbRecs=10) |  |  |
| SbSPL2 | 238 | 6 | TATATA | [AT~TATA-box](http://bioinformatics.psb.ugent.be/webtools/plantcare/cgi-bin/show_site_info.htpl?QWhere=ID_of_Site%20like%20%27TATATA%27&StartAt=0&NbRecs=10) |  |  |
| SbSPL2 | 1354 | 4 | CAAT | [CAAT-box](http://bioinformatics.psb.ugent.be/webtools/plantcare/cgi-bin/show_site_info.htpl?QWhere=ID_of_Site%20like%20%27CAAT%27&StartAt=0&NbRecs=10) |  |  |
| SbSPL2 | 1349 | 4 | CAAT | [CAAT-box](http://bioinformatics.psb.ugent.be/webtools/plantcare/cgi-bin/show_site_info.htpl?QWhere=ID_of_Site%20like%20%27CAAT%27&StartAt=0&NbRecs=10) |  |  |
| SbSPL2 | 1146 | 4 | CAAT | [CAAT-box](http://bioinformatics.psb.ugent.be/webtools/plantcare/cgi-bin/show_site_info.htpl?QWhere=ID_of_Site%20like%20%27CAAT%27&StartAt=0&NbRecs=10) |  |  |
| SbSPL2 | 1922 | 5 | CAAAT | [CAAT-box](http://bioinformatics.psb.ugent.be/webtools/plantcare/cgi-bin/show_site_info.htpl?QWhere=ID_of_Site%20like%20%27CAAAT%27&StartAt=0&NbRecs=10) | common cis-acting element in promoter and enhancer regions |  |
| SbSPL2 | 557 | 5 | CAAAT | [CAAT-box](http://bioinformatics.psb.ugent.be/webtools/plantcare/cgi-bin/show_site_info.htpl?QWhere=ID_of_Site%20like%20%27CAAAT%27&StartAt=0&NbRecs=10) | common cis-acting element in promoter and enhancer regions |  |
| SbSPL2 | 1320 | 4 | CAAT | [CAAT-box](http://bioinformatics.psb.ugent.be/webtools/plantcare/cgi-bin/show_site_info.htpl?QWhere=ID_of_Site%20like%20%27CAAT%27&StartAt=0&NbRecs=10) |  |  |
| SbSPL2 | 831 | 4 | CAAT | [CAAT-box](http://bioinformatics.psb.ugent.be/webtools/plantcare/cgi-bin/show_site_info.htpl?QWhere=ID_of_Site%20like%20%27CAAT%27&StartAt=0&NbRecs=10) |  |  |
| SbSPL2 | 273 | 5 | CCAAT | [CAAT-box](http://bioinformatics.psb.ugent.be/webtools/plantcare/cgi-bin/show_site_info.htpl?QWhere=ID_of_Site%20like%20%27CCAAT%27&StartAt=0&NbRecs=10) | common cis-acting element in promoter and enhancer regions |  |
| SbSPL2 | 830 | 5 | CCAAT | [CAAT-box](http://bioinformatics.psb.ugent.be/webtools/plantcare/cgi-bin/show_site_info.htpl?QWhere=ID_of_Site%20like%20%27CCAAT%27&StartAt=0&NbRecs=10) | common cis-acting element in promoter and enhancer regions |  |
| SbSPL2 | 65 | 4 | CAAT | [CAAT-box](http://bioinformatics.psb.ugent.be/webtools/plantcare/cgi-bin/show_site_info.htpl?QWhere=ID_of_Site%20like%20%27CAAT%27&StartAt=0&NbRecs=10) |  |  |
| SbSPL2 | 579 | 4 | CAAT | [CAAT-box](http://bioinformatics.psb.ugent.be/webtools/plantcare/cgi-bin/show_site_info.htpl?QWhere=ID_of_Site%20like%20%27CAAT%27&StartAt=0&NbRecs=10) |  |  |
| SbSPL2 | 321 | 4 | CAAT | [CAAT-box](http://bioinformatics.psb.ugent.be/webtools/plantcare/cgi-bin/show_site_info.htpl?QWhere=ID_of_Site%20like%20%27CAAT%27&StartAt=0&NbRecs=10) |  |  |
| SbSPL2 | 465 | 4 | CAAT | [CAAT-box](http://bioinformatics.psb.ugent.be/webtools/plantcare/cgi-bin/show_site_info.htpl?QWhere=ID_of_Site%20like%20%27CAAT%27&StartAt=0&NbRecs=10) |  |  |
| SbSPL2 | 935 | 4 | CAAT | [CAAT-box](http://bioinformatics.psb.ugent.be/webtools/plantcare/cgi-bin/show_site_info.htpl?QWhere=ID_of_Site%20like%20%27CAAT%27&StartAt=0&NbRecs=10) |  |  |
| SbSPL2 | 1084 | 4 | CAAT | [CAAT-box](http://bioinformatics.psb.ugent.be/webtools/plantcare/cgi-bin/show_site_info.htpl?QWhere=ID_of_Site%20like%20%27CAAT%27&StartAt=0&NbRecs=10) |  |  |
| SbSPL2 | 274 | 4 | CAAT | [CAAT-box](http://bioinformatics.psb.ugent.be/webtools/plantcare/cgi-bin/show_site_info.htpl?QWhere=ID_of_Site%20like%20%27CAAT%27&StartAt=0&NbRecs=10) |  |  |
| SbSPL2 | 1688 | 5 | CAAAT | [CAAT-box](http://bioinformatics.psb.ugent.be/webtools/plantcare/cgi-bin/show_site_info.htpl?QWhere=ID_of_Site%20like%20%27CAAAT%27&StartAt=0&NbRecs=10) | common cis-acting element in promoter and enhancer regions |  |
| SbSPL2 | 440 | 4 | CAAT | [CAAT-box](http://bioinformatics.psb.ugent.be/webtools/plantcare/cgi-bin/show_site_info.htpl?QWhere=ID_of_Site%20like%20%27CAAT%27&StartAt=0&NbRecs=10) |  |  |
| SbSPL2 | 743 | 5 | CAAAT | [CAAT-box](http://bioinformatics.psb.ugent.be/webtools/plantcare/cgi-bin/show_site_info.htpl?QWhere=ID_of_Site%20like%20%27CAAAT%27&StartAt=0&NbRecs=10) | common cis-acting element in promoter and enhancer regions |  |
| SbSPL2 | 1158 | 4 | CAAT | [CAAT-box](http://bioinformatics.psb.ugent.be/webtools/plantcare/cgi-bin/show_site_info.htpl?QWhere=ID_of_Site%20like%20%27CAAT%27&StartAt=0&NbRecs=10) |  |  |
| SbSPL2 | 729 | 4 | CAAT | [CAAT-box](http://bioinformatics.psb.ugent.be/webtools/plantcare/cgi-bin/show_site_info.htpl?QWhere=ID_of_Site%20like%20%27CAAT%27&StartAt=0&NbRecs=10) |  |  |
| SbSPL2 | 1291 | 4 | CAAT | [CAAT-box](http://bioinformatics.psb.ugent.be/webtools/plantcare/cgi-bin/show_site_info.htpl?QWhere=ID_of_Site%20like%20%27CAAT%27&StartAt=0&NbRecs=10) |  |  |
| SbSPL2 | 957 | 5 | CAAAT | [CAAT-box](http://bioinformatics.psb.ugent.be/webtools/plantcare/cgi-bin/show_site_info.htpl?QWhere=ID_of_Site%20like%20%27CAAAT%27&StartAt=0&NbRecs=10) | common cis-acting element in promoter and enhancer regions |  |
| SbSPL2 | 10 | 4 | CAAT | [CAAT-box](http://bioinformatics.psb.ugent.be/webtools/plantcare/cgi-bin/show_site_info.htpl?QWhere=ID_of_Site%20like%20%27CAAT%27&StartAt=0&NbRecs=10) |  |  |
| SbSPL2 | 885 | 4 | CAAT | [CAAT-box](http://bioinformatics.psb.ugent.be/webtools/plantcare/cgi-bin/show_site_info.htpl?QWhere=ID_of_Site%20like%20%27CAAT%27&StartAt=0&NbRecs=10) |  |  |
| SbSPL2 | 1148 | 4 | CAAT | [CAAT-box](http://bioinformatics.psb.ugent.be/webtools/plantcare/cgi-bin/show_site_info.htpl?QWhere=ID_of_Site%20like%20%27CAAT%27&StartAt=0&NbRecs=10) |  |  |
| SbSPL2 | 453 | 5 | CAAAT | [CAAT-box](http://bioinformatics.psb.ugent.be/webtools/plantcare/cgi-bin/show_site_info.htpl?QWhere=ID_of_Site%20like%20%27CAAAT%27&StartAt=0&NbRecs=10) | common cis-acting element in promoter and enhancer regions |  |
| SbSPL2 | 145 | 4 | CAAT | [CAAT-box](http://bioinformatics.psb.ugent.be/webtools/plantcare/cgi-bin/show_site_info.htpl?QWhere=ID_of_Site%20like%20%27CAAT%27&StartAt=0&NbRecs=10) |  |  |
| SbSPL2 | 1074 | 5 | CAAAT | [CAAT-box](http://bioinformatics.psb.ugent.be/webtools/plantcare/cgi-bin/show_site_info.htpl?QWhere=ID_of_Site%20like%20%27CAAAT%27&StartAt=0&NbRecs=10) | common cis-acting element in promoter and enhancer regions |  |
| SbSPL2 | 144 | 5 | CCAAT | [CAAT-box](http://bioinformatics.psb.ugent.be/webtools/plantcare/cgi-bin/show_site_info.htpl?QWhere=ID_of_Site%20like%20%27CCAAT%27&StartAt=0&NbRecs=10) | common cis-acting element in promoter and enhancer regions |  |
| SbSPL2 | 56 | 6 | GCCACT | [CAT-box](http://bioinformatics.psb.ugent.be/webtools/plantcare/cgi-bin/show_site_info.htpl?QWhere=ID_of_Site%20like%20%27GCCACT%27&StartAt=0&NbRecs=10) | cis-acting regulatory element related to meristem expression |  |
| SbSPL2 | 113 | 10 | CTATTCTCATT | [F-box](http://bioinformatics.psb.ugent.be/webtools/plantcare/cgi-bin/show_site_info.htpl?QWhere=ID_of_Site%20like%20%27CTATTCTCATT%27&StartAt=0&NbRecs=10) |  |  |
| SbSPL2 | 210 | 8 | ATAGATAA | [GA-motif](http://bioinformatics.psb.ugent.be/webtools/plantcare/cgi-bin/show_site_info.htpl?QWhere=ID_of_Site%20like%20%27ATAGATAA%27&StartAt=0&NbRecs=10) | part of a light responsive element | Light responsiveness |
| SbSPL2 | 1261 | 9 | AAGGATAAGG | [GATA-motif](http://bioinformatics.psb.ugent.be/webtools/plantcare/cgi-bin/show_site_info.htpl?QWhere=ID_of_Site%20like%20%27AAGGATAAGG%27&StartAt=0&NbRecs=10) | part of a light responsive element | Light responsiveness |
| SbSPL2 | 967 | 6 | CCCCCG | [GC-motif](http://bioinformatics.psb.ugent.be/webtools/plantcare/cgi-bin/show_site_info.htpl?QWhere=ID_of_Site%20like%20%27CCCCCG%27&StartAt=0&NbRecs=10) | enhancer-like element involved in anoxic specific inducibility |  |
| SbSPL2 | 1571 | 9 | GTGTGTGAA | [GT1-motif](http://bioinformatics.psb.ugent.be/webtools/plantcare/cgi-bin/show_site_info.htpl?QWhere=ID_of_Site%20like%20%27GTGTGTGAA%27&StartAt=0&NbRecs=10) | light responsive element | Light responsiveness |
| SbSPL2 | 1100 | 9 | CCTACCNNNNNNNCTNNNNA | [H-box](http://bioinformatics.psb.ugent.be/webtools/plantcare/cgi-bin/show_site_info.htpl?QWhere=ID_of_Site%20like%20%27CCTACCNNNNNNNCTNNNNA%27&StartAt=0&NbRecs=10) |  |  |
| SbSPL2 | 1262 | 9 | ccttatcct | [I-box](http://bioinformatics.psb.ugent.be/webtools/plantcare/cgi-bin/show_site_info.htpl?QWhere=ID_of_Site%20like%20%27ccttatcct%27&StartAt=0&NbRecs=10) | part of a light responsive element | Light responsiveness |
| SbSPL2 | 1263 | 9 | gGATAAGGTG | [I-box](http://bioinformatics.psb.ugent.be/webtools/plantcare/cgi-bin/show_site_info.htpl?QWhere=ID_of_Site%20like%20%27gGATAAGGTG%27&StartAt=0&NbRecs=10) | part of a light responsive element | Light responsiveness |
| SbSPL2 | 1133 | 8 | CTTTATCA | [LAMP-element](http://bioinformatics.psb.ugent.be/webtools/plantcare/cgi-bin/show_site_info.htpl?QWhere=ID_of_Site%20like%20%27CTTTATCA%27&StartAt=0&NbRecs=10) | part of a light responsive element | Light responsiveness |
| SbSPL2 | 1529 | 6 | CAACCA | [MYB](http://bioinformatics.psb.ugent.be/webtools/plantcare/cgi-bin/show_site_info.htpl?QWhere=ID_of_Site%20like%20%27CAACCA%27&StartAt=0&NbRecs=10) |  |  |
| SbSPL2 | 1120 | 6 | CAACCA | [MYB](http://bioinformatics.psb.ugent.be/webtools/plantcare/cgi-bin/show_site_info.htpl?QWhere=ID_of_Site%20like%20%27CAACCA%27&StartAt=0&NbRecs=10) |  |  |
| SbSPL2 | 658 | 6 | CAACCA | [MYB](http://bioinformatics.psb.ugent.be/webtools/plantcare/cgi-bin/show_site_info.htpl?QWhere=ID_of_Site%20like%20%27CAACCA%27&StartAt=0&NbRecs=10) |  |  |
| SbSPL2 | 742 | 6 | CATTTG | [MYC](http://bioinformatics.psb.ugent.be/webtools/plantcare/cgi-bin/show_site_info.htpl?QWhere=ID_of_Site%20like%20%27CATTTG%27&StartAt=0&NbRecs=10) |  |  |
| SbSPL2 | 1146 | 6 | CAATTG | [MYC](http://bioinformatics.psb.ugent.be/webtools/plantcare/cgi-bin/show_site_info.htpl?QWhere=ID_of_Site%20like%20%27CAATTG%27&StartAt=0&NbRecs=10) |  |  |
| SbSPL2 | 956 | 6 | CATTTG | [MYC](http://bioinformatics.psb.ugent.be/webtools/plantcare/cgi-bin/show_site_info.htpl?QWhere=ID_of_Site%20like%20%27CATTTG%27&StartAt=0&NbRecs=10) |  |  |
| SbSPL2 | 1687 | 6 | CATTTG | [MYC](http://bioinformatics.psb.ugent.be/webtools/plantcare/cgi-bin/show_site_info.htpl?QWhere=ID_of_Site%20like%20%27CATTTG%27&StartAt=0&NbRecs=10) |  |  |
| SbSPL2 | 1066 | 6 | TAACTG | [Myb](http://bioinformatics.psb.ugent.be/webtools/plantcare/cgi-bin/show_site_info.htpl?QWhere=ID_of_Site%20like%20%27TAACTG%27&StartAt=0&NbRecs=10) |  |  |
| SbSPL2 | 1448 | 7 | CCTTTTG | [P-box](http://bioinformatics.psb.ugent.be/webtools/plantcare/cgi-bin/show_site_info.htpl?QWhere=ID_of_Site%20like%20%27CCTTTTG%27&StartAt=0&NbRecs=10) | gibberellin-responsive element | Hormone responsiveness |
| SbSPL2 | 1105 | 5 | AGGGG | [STRE](http://bioinformatics.psb.ugent.be/webtools/plantcare/cgi-bin/show_site_info.htpl?QWhere=ID_of_Site%20like%20%27AGGGG%27&StartAt=0&NbRecs=10) |  |  |
| SbSPL2 | 1579 | 5 | AGGGG | [STRE](http://bioinformatics.psb.ugent.be/webtools/plantcare/cgi-bin/show_site_info.htpl?QWhere=ID_of_Site%20like%20%27AGGGG%27&StartAt=0&NbRecs=10) |  |  |
| SbSPL2 | 1170 | 5 | AGGGG | [STRE](http://bioinformatics.psb.ugent.be/webtools/plantcare/cgi-bin/show_site_info.htpl?QWhere=ID_of_Site%20like%20%27AGGGG%27&StartAt=0&NbRecs=10) |  |  |
| SbSPL2 | 1374 | 4 | TATA | [TATA-box](http://bioinformatics.psb.ugent.be/webtools/plantcare/cgi-bin/show_site_info.htpl?QWhere=ID_of_Site%20like%20%27TATA%27&StartAt=0&NbRecs=10) | core promoter element around -30 of transcription start |  |
| SbSPL2 | 1864 | 4 | TATA | [TATA-box](http://bioinformatics.psb.ugent.be/webtools/plantcare/cgi-bin/show_site_info.htpl?QWhere=ID_of_Site%20like%20%27TATA%27&StartAt=0&NbRecs=10) | core promoter element around -30 of transcription start |  |
| SbSPL2 | 1694 | 4 | TATA | [TATA-box](http://bioinformatics.psb.ugent.be/webtools/plantcare/cgi-bin/show_site_info.htpl?QWhere=ID_of_Site%20like%20%27TATA%27&StartAt=0&NbRecs=10) | core promoter element around -30 of transcription start |  |
| SbSPL2 | 1022 | 4 | TATA | [TATA-box](http://bioinformatics.psb.ugent.be/webtools/plantcare/cgi-bin/show_site_info.htpl?QWhere=ID_of_Site%20like%20%27TATA%27&StartAt=0&NbRecs=10) | core promoter element around -30 of transcription start |  |
| SbSPL2 | 432 | 6 | TATATA | [TATA-box](http://bioinformatics.psb.ugent.be/webtools/plantcare/cgi-bin/show_site_info.htpl?QWhere=ID_of_Site%20like%20%27TATATA%27&StartAt=0&NbRecs=10) | core promoter element around -30 of transcription start |  |
| SbSPL2 | 1809 | 4 | TATA | [TATA-box](http://bioinformatics.psb.ugent.be/webtools/plantcare/cgi-bin/show_site_info.htpl?QWhere=ID_of_Site%20like%20%27TATA%27&StartAt=0&NbRecs=10) | core promoter element around -30 of transcription start |  |
| SbSPL2 | 1800 | 6 | TATACA | [TATA-box](http://bioinformatics.psb.ugent.be/webtools/plantcare/cgi-bin/show_site_info.htpl?QWhere=ID_of_Site%20like%20%27TATACA%27&StartAt=0&NbRecs=10) | core promoter element around -30 of transcription start |  |
| SbSPL2 | 1735 | 4 | TATA | [TATA-box](http://bioinformatics.psb.ugent.be/webtools/plantcare/cgi-bin/show_site_info.htpl?QWhere=ID_of_Site%20like%20%27TATA%27&StartAt=0&NbRecs=10) | core promoter element around -30 of transcription start |  |
| SbSPL2 | 1774 | 8 | TACATAAA | [TATA-box](http://bioinformatics.psb.ugent.be/webtools/plantcare/cgi-bin/show_site_info.htpl?QWhere=ID_of_Site%20like%20%27TACATAAA%27&StartAt=0&NbRecs=10) | core promoter element around -30 of transcription start |  |
| SbSPL2 | 288 | 4 | TATA | [TATA-box](http://bioinformatics.psb.ugent.be/webtools/plantcare/cgi-bin/show_site_info.htpl?QWhere=ID_of_Site%20like%20%27TATA%27&StartAt=0&NbRecs=10) | core promoter element around -30 of transcription start |  |
| SbSPL2 | 1543 | 4 | TATA | [TATA-box](http://bioinformatics.psb.ugent.be/webtools/plantcare/cgi-bin/show_site_info.htpl?QWhere=ID_of_Site%20like%20%27TATA%27&StartAt=0&NbRecs=10) | core promoter element around -30 of transcription start |  |
| SbSPL2 | 1286 | 6 | TATATA | [TATA-box](http://bioinformatics.psb.ugent.be/webtools/plantcare/cgi-bin/show_site_info.htpl?QWhere=ID_of_Site%20like%20%27TATATA%27&StartAt=0&NbRecs=10) | core promoter element around -30 of transcription start |  |
| SbSPL2 | 240 | 4 | TATA | [TATA-box](http://bioinformatics.psb.ugent.be/webtools/plantcare/cgi-bin/show_site_info.htpl?QWhere=ID_of_Site%20like%20%27TATA%27&StartAt=0&NbRecs=10) | core promoter element around -30 of transcription start |  |
| SbSPL2 | 1600 | 4 | TATA | [TATA-box](http://bioinformatics.psb.ugent.be/webtools/plantcare/cgi-bin/show_site_info.htpl?QWhere=ID_of_Site%20like%20%27TATA%27&StartAt=0&NbRecs=10) | core promoter element around -30 of transcription start |  |
| SbSPL2 | 1167 | 4 | TATA | [TATA-box](http://bioinformatics.psb.ugent.be/webtools/plantcare/cgi-bin/show_site_info.htpl?QWhere=ID_of_Site%20like%20%27TATA%27&StartAt=0&NbRecs=10) | core promoter element around -30 of transcription start |  |
| SbSPL2 | 1288 | 4 | TATA | [TATA-box](http://bioinformatics.psb.ugent.be/webtools/plantcare/cgi-bin/show_site_info.htpl?QWhere=ID_of_Site%20like%20%27TATA%27&StartAt=0&NbRecs=10) | core promoter element around -30 of transcription start |  |
| SbSPL2 | 1995 | 6 | ATTATA | [TATA-box](http://bioinformatics.psb.ugent.be/webtools/plantcare/cgi-bin/show_site_info.htpl?QWhere=ID_of_Site%20like%20%27ATTATA%27&StartAt=0&NbRecs=10) | core promoter element around -30 of transcription start |  |
| SbSPL2 | 938 | 4 | TATA | [TATA-box](http://bioinformatics.psb.ugent.be/webtools/plantcare/cgi-bin/show_site_info.htpl?QWhere=ID_of_Site%20like%20%27TATA%27&StartAt=0&NbRecs=10) | core promoter element around -30 of transcription start |  |
| SbSPL2 | 1503 | 5 | TATAA | [TATA-box](http://bioinformatics.psb.ugent.be/webtools/plantcare/cgi-bin/show_site_info.htpl?QWhere=ID_of_Site%20like%20%27TATAA%27&StartAt=0&NbRecs=10) | core promoter element around -30 of transcription start |  |
| SbSPL2 | 1599 | 5 | TATAA | [TATA-box](http://bioinformatics.psb.ugent.be/webtools/plantcare/cgi-bin/show_site_info.htpl?QWhere=ID_of_Site%20like%20%27TATAA%27&StartAt=0&NbRecs=10) | core promoter element around -30 of transcription start |  |
| SbSPL2 | 176 | 4 | TATA | [TATA-box](http://bioinformatics.psb.ugent.be/webtools/plantcare/cgi-bin/show_site_info.htpl?QWhere=ID_of_Site%20like%20%27TATA%27&StartAt=0&NbRecs=10) | core promoter element around -30 of transcription start |  |
| SbSPL2 | 239 | 6 | ATATAT | [TATA-box](http://bioinformatics.psb.ugent.be/webtools/plantcare/cgi-bin/show_site_info.htpl?QWhere=ID_of_Site%20like%20%27ATATAT%27&StartAt=0&NbRecs=10) | core promoter element around -30 of transcription start |  |
| SbSPL2 | 1863 | 5 | TATAA | [TATA-box](http://bioinformatics.psb.ugent.be/webtools/plantcare/cgi-bin/show_site_info.htpl?QWhere=ID_of_Site%20like%20%27TATAA%27&StartAt=0&NbRecs=10) | core promoter element around -30 of transcription start |  |
| SbSPL2 | 1372 | 6 | TATATA | [TATA-box](http://bioinformatics.psb.ugent.be/webtools/plantcare/cgi-bin/show_site_info.htpl?QWhere=ID_of_Site%20like%20%27TATATA%27&StartAt=0&NbRecs=10) | core promoter element around -30 of transcription start |  |
| SbSPL2 | 1598 | 6 | ATTATA | [TATA-box](http://bioinformatics.psb.ugent.be/webtools/plantcare/cgi-bin/show_site_info.htpl?QWhere=ID_of_Site%20like%20%27ATTATA%27&StartAt=0&NbRecs=10) | core promoter element around -30 of transcription start |  |
| SbSPL2 | 434 | 4 | TATA | [TATA-box](http://bioinformatics.psb.ugent.be/webtools/plantcare/cgi-bin/show_site_info.htpl?QWhere=ID_of_Site%20like%20%27TATA%27&StartAt=0&NbRecs=10) | core promoter element around -30 of transcription start |  |
| SbSPL2 | 1285 | 6 | ATATAT | [TATA-box](http://bioinformatics.psb.ugent.be/webtools/plantcare/cgi-bin/show_site_info.htpl?QWhere=ID_of_Site%20like%20%27ATATAT%27&StartAt=0&NbRecs=10) | core promoter element around -30 of transcription start |  |
| SbSPL2 | 630 | 4 | TATA | [TATA-box](http://bioinformatics.psb.ugent.be/webtools/plantcare/cgi-bin/show_site_info.htpl?QWhere=ID_of_Site%20like%20%27TATA%27&StartAt=0&NbRecs=10) | core promoter element around -30 of transcription start |  |
| SbSPL2 | 1996 | 5 | TATAA | [TATA-box](http://bioinformatics.psb.ugent.be/webtools/plantcare/cgi-bin/show_site_info.htpl?QWhere=ID_of_Site%20like%20%27TATAA%27&StartAt=0&NbRecs=10) | core promoter element around -30 of transcription start |  |
| SbSPL2 | 286 | 6 | TATATA | [TATA-box](http://bioinformatics.psb.ugent.be/webtools/plantcare/cgi-bin/show_site_info.htpl?QWhere=ID_of_Site%20like%20%27TATATA%27&StartAt=0&NbRecs=10) | core promoter element around -30 of transcription start |  |
| SbSPL2 | 1734 | 6 | ATATAA | [TATA-box](http://bioinformatics.psb.ugent.be/webtools/plantcare/cgi-bin/show_site_info.htpl?QWhere=ID_of_Site%20like%20%27ATATAA%27&StartAt=0&NbRecs=10) | core promoter element around -30 of transcription start |  |
| SbSPL2 | 431 | 6 | ATATAT | [TATA-box](http://bioinformatics.psb.ugent.be/webtools/plantcare/cgi-bin/show_site_info.htpl?QWhere=ID_of_Site%20like%20%27ATATAT%27&StartAt=0&NbRecs=10) | core promoter element around -30 of transcription start |  |
| SbSPL2 | 1802 | 4 | TATA | [TATA-box](http://bioinformatics.psb.ugent.be/webtools/plantcare/cgi-bin/show_site_info.htpl?QWhere=ID_of_Site%20like%20%27TATA%27&StartAt=0&NbRecs=10) | core promoter element around -30 of transcription start |  |
| SbSPL2 | 1287 | 6 | ATATAT | [TATA-box](http://bioinformatics.psb.ugent.be/webtools/plantcare/cgi-bin/show_site_info.htpl?QWhere=ID_of_Site%20like%20%27ATATAT%27&StartAt=0&NbRecs=10) | core promoter element around -30 of transcription start |  |
| SbSPL2 | 1502 | 6 | ATTATA | [TATA-box](http://bioinformatics.psb.ugent.be/webtools/plantcare/cgi-bin/show_site_info.htpl?QWhere=ID_of_Site%20like%20%27ATTATA%27&StartAt=0&NbRecs=10) | core promoter element around -30 of transcription start |  |
| SbSPL2 | 175 | 5 | TATAA | [TATA-box](http://bioinformatics.psb.ugent.be/webtools/plantcare/cgi-bin/show_site_info.htpl?QWhere=ID_of_Site%20like%20%27TATAA%27&StartAt=0&NbRecs=10) | core promoter element around -30 of transcription start |  |
| SbSPL2 | 1997 | 4 | TATA | [TATA-box](http://bioinformatics.psb.ugent.be/webtools/plantcare/cgi-bin/show_site_info.htpl?QWhere=ID_of_Site%20like%20%27TATA%27&StartAt=0&NbRecs=10) | core promoter element around -30 of transcription start |  |
| SbSPL2 | 507 | 4 | TATA | [TATA-box](http://bioinformatics.psb.ugent.be/webtools/plantcare/cgi-bin/show_site_info.htpl?QWhere=ID_of_Site%20like%20%27TATA%27&StartAt=0&NbRecs=10) | core promoter element around -30 of transcription start |  |
| SbSPL2 | 1622 | 5 | TATAA | [TATA-box](http://bioinformatics.psb.ugent.be/webtools/plantcare/cgi-bin/show_site_info.htpl?QWhere=ID_of_Site%20like%20%27TATAA%27&StartAt=0&NbRecs=10) | core promoter element around -30 of transcription start |  |
| SbSPL2 | 1021 | 6 | ATATAA | [TATA-box](http://bioinformatics.psb.ugent.be/webtools/plantcare/cgi-bin/show_site_info.htpl?QWhere=ID_of_Site%20like%20%27ATATAA%27&StartAt=0&NbRecs=10) | core promoter element around -30 of transcription start |  |
| SbSPL2 | 1504 | 4 | TATA | [TATA-box](http://bioinformatics.psb.ugent.be/webtools/plantcare/cgi-bin/show_site_info.htpl?QWhere=ID_of_Site%20like%20%27TATA%27&StartAt=0&NbRecs=10) | core promoter element around -30 of transcription start |  |
| SbSPL2 | 238 | 6 | TATATA | [TATA-box](http://bioinformatics.psb.ugent.be/webtools/plantcare/cgi-bin/show_site_info.htpl?QWhere=ID_of_Site%20like%20%27TATATA%27&StartAt=0&NbRecs=10) | core promoter element around -30 of transcription start |  |
| SbSPL2 | 1623 | 4 | TATA | [TATA-box](http://bioinformatics.psb.ugent.be/webtools/plantcare/cgi-bin/show_site_info.htpl?QWhere=ID_of_Site%20like%20%27TATA%27&StartAt=0&NbRecs=10) | core promoter element around -30 of transcription start |  |
| SbSPL2 | 1373 | 6 | ATATAA | [TATA-box](http://bioinformatics.psb.ugent.be/webtools/plantcare/cgi-bin/show_site_info.htpl?QWhere=ID_of_Site%20like%20%27ATATAA%27&StartAt=0&NbRecs=10) | core promoter element around -30 of transcription start |  |
| SbSPL2 | 433 | 6 | ATATAA | [TATA-box](http://bioinformatics.psb.ugent.be/webtools/plantcare/cgi-bin/show_site_info.htpl?QWhere=ID_of_Site%20like%20%27ATATAA%27&StartAt=0&NbRecs=10) | core promoter element around -30 of transcription start |  |
| SbSPL2 | 1371 | 6 | ATATAT | [TATA-box](http://bioinformatics.psb.ugent.be/webtools/plantcare/cgi-bin/show_site_info.htpl?QWhere=ID_of_Site%20like%20%27ATATAT%27&StartAt=0&NbRecs=10) | core promoter element around -30 of transcription start |  |
| SbSPL2 | 1586 | 7 | TCTCCCT | [TCCC-motif](http://bioinformatics.psb.ugent.be/webtools/plantcare/cgi-bin/show_site_info.htpl?QWhere=ID_of_Site%20like%20%27TCTCCCT%27&StartAt=0&NbRecs=10) | part of a light responsive element | Light responsiveness |
| SbSPL2 | 1153 | 6 | TCTTAC | [TCT-motif](http://bioinformatics.psb.ugent.be/webtools/plantcare/cgi-bin/show_site_info.htpl?QWhere=ID_of_Site%20like%20%27TCTTAC%27&StartAt=0&NbRecs=10) | part of a light responsive element | Light responsiveness |
| SbSPL2 | 617 | 5 | CGTGG | [Unnamed__1](http://bioinformatics.psb.ugent.be/webtools/plantcare/cgi-bin/show_site_info.htpl?QWhere=ID_of_Site%20like%20%27CGTGG%27&StartAt=0&NbRecs=10) |  |  |
| SbSPL2 | 1269 | 4 | CTCC | [Unnamed__4](http://bioinformatics.psb.ugent.be/webtools/plantcare/cgi-bin/show_site_info.htpl?QWhere=ID_of_Site%20like%20%27CTCC%27&StartAt=0&NbRecs=10) |  |  |
| SbSPL2 | 1582 | 4 | CTCC | [Unnamed__4](http://bioinformatics.psb.ugent.be/webtools/plantcare/cgi-bin/show_site_info.htpl?QWhere=ID_of_Site%20like%20%27CTCC%27&StartAt=0&NbRecs=10) |  |  |
| SbSPL2 | 1841 | 4 | CTCC | [Unnamed__4](http://bioinformatics.psb.ugent.be/webtools/plantcare/cgi-bin/show_site_info.htpl?QWhere=ID_of_Site%20like%20%27CTCC%27&StartAt=0&NbRecs=10) |  |  |
| SbSPL2 | 622 | 4 | CTCC | [Unnamed__4](http://bioinformatics.psb.ugent.be/webtools/plantcare/cgi-bin/show_site_info.htpl?QWhere=ID_of_Site%20like%20%27CTCC%27&StartAt=0&NbRecs=10) |  |  |
| SbSPL2 | 54 | 4 | CTCC | [Unnamed__4](http://bioinformatics.psb.ugent.be/webtools/plantcare/cgi-bin/show_site_info.htpl?QWhere=ID_of_Site%20like%20%27CTCC%27&StartAt=0&NbRecs=10) |  |  |
| SbSPL2 | 1588 | 4 | CTCC | [Unnamed__4](http://bioinformatics.psb.ugent.be/webtools/plantcare/cgi-bin/show_site_info.htpl?QWhere=ID_of_Site%20like%20%27CTCC%27&StartAt=0&NbRecs=10) |  |  |
| SbSPL2 | 1926 | 4 | CTCC | [Unnamed__4](http://bioinformatics.psb.ugent.be/webtools/plantcare/cgi-bin/show_site_info.htpl?QWhere=ID_of_Site%20like%20%27CTCC%27&StartAt=0&NbRecs=10) |  |  |
| SbSPL2 | 1453 | 4 | CTCC | [Unnamed__4](http://bioinformatics.psb.ugent.be/webtools/plantcare/cgi-bin/show_site_info.htpl?QWhere=ID_of_Site%20like%20%27CTCC%27&StartAt=0&NbRecs=10) |  |  |
| SbSPL2 | 1720 | 6 | CCACCT | [WRE3](http://bioinformatics.psb.ugent.be/webtools/plantcare/cgi-bin/show_site_info.htpl?QWhere=ID_of_Site%20like%20%27CCACCT%27&StartAt=0&NbRecs=10) |  |  |
| SbSPL2 | 166 | 6 | CCACCT | [WRE3](http://bioinformatics.psb.ugent.be/webtools/plantcare/cgi-bin/show_site_info.htpl?QWhere=ID_of_Site%20like%20%27CCACCT%27&StartAt=0&NbRecs=10) |  |  |
| SbSPL2 | 885 | 9 | CAATTACAT | [WUN-motif](http://bioinformatics.psb.ugent.be/webtools/plantcare/cgi-bin/show_site_info.htpl?QWhere=ID_of_Site%20like%20%27CAATTACAT%27&StartAt=0&NbRecs=10) |  |  |
| SbSPL2 | 44 | 8 | AAATTACT | [WUN-motif](http://bioinformatics.psb.ugent.be/webtools/plantcare/cgi-bin/show_site_info.htpl?QWhere=ID_of_Site%20like%20%27AAATTACT%27&StartAt=0&NbRecs=10) |  |  |
| SbSPL2 | 1870 | 8 | TTACTTAA | [chs-CMA1a](http://bioinformatics.psb.ugent.be/webtools/plantcare/cgi-bin/show_site_info.htpl?QWhere=ID_of_Site%20like%20%27TTACTTAA%27&StartAt=0&NbRecs=10) | TTACTTAA |  |
| SbSPL3 | 1053 | 6 | CCGTCC | [A-box](http://bioinformatics.psb.ugent.be/webtools/plantcare/cgi-bin/show_site_info.htpl?QWhere=ID_of_Site%20like%20%27CCGTCC%27&StartAt=0&NbRecs=10) | cis-acting regulatory element |  |
| SbSPL3 | 822 | 7 | GAAAGAA | [AAGAA-motif](http://bioinformatics.psb.ugent.be/webtools/plantcare/cgi-bin/show_site_info.htpl?QWhere=ID_of_Site%20like%20%27GAAAGAA%27&StartAt=0&NbRecs=10) |  |  |
| SbSPL3 | 826 | 7 | GAAAGAA | [AAGAA-motif](http://bioinformatics.psb.ugent.be/webtools/plantcare/cgi-bin/show_site_info.htpl?QWhere=ID_of_Site%20like%20%27GAAAGAA%27&StartAt=0&NbRecs=10) |  |  |
| SbSPL3 | 818 | 7 | GAAAGAA | [AAGAA-motif](http://bioinformatics.psb.ugent.be/webtools/plantcare/cgi-bin/show_site_info.htpl?QWhere=ID_of_Site%20like%20%27GAAAGAA%27&StartAt=0&NbRecs=10) |  |  |
| SbSPL3 | 492 | 7 | GAAAGAA | [AAGAA-motif](http://bioinformatics.psb.ugent.be/webtools/plantcare/cgi-bin/show_site_info.htpl?QWhere=ID_of_Site%20like%20%27GAAAGAA%27&StartAt=0&NbRecs=10) |  |  |
| SbSPL3 | 753 | 5 | ACGTG | [ABRE](http://bioinformatics.psb.ugent.be/webtools/plantcare/cgi-bin/show_site_info.htpl?QWhere=ID_of_Site%20like%20%27ACGTG%27&StartAt=0&NbRecs=10) | cis-acting element involved in the abscisic acid responsiveness | Hormone responsiveness |
| SbSPL3 | 405 | 5 | ACGTG | [ABRE](http://bioinformatics.psb.ugent.be/webtools/plantcare/cgi-bin/show_site_info.htpl?QWhere=ID_of_Site%20like%20%27ACGTG%27&StartAt=0&NbRecs=10) | cis-acting element involved in the abscisic acid responsiveness | Hormone responsiveness |
| SbSPL3 | 402 | 9 | GACACGTATG | [ACE](http://bioinformatics.psb.ugent.be/webtools/plantcare/cgi-bin/show_site_info.htpl?QWhere=ID_of_Site%20like%20%27GACACGTATG%27&StartAt=0&NbRecs=10) | cis-acting element involved in light responsiveness | Light responsiveness |
| SbSPL3 | 615 | 6 | TATATA | [AT~TATA-box](http://bioinformatics.psb.ugent.be/webtools/plantcare/cgi-bin/show_site_info.htpl?QWhere=ID_of_Site%20like%20%27TATATA%27&StartAt=0&NbRecs=10) |  |  |
| SbSPL3 | 613 | 6 | TATATA | [AT~TATA-box](http://bioinformatics.psb.ugent.be/webtools/plantcare/cgi-bin/show_site_info.htpl?QWhere=ID_of_Site%20like%20%27TATATA%27&StartAt=0&NbRecs=10) |  |  |
| SbSPL3 | 619 | 6 | TATATA | [AT~TATA-box](http://bioinformatics.psb.ugent.be/webtools/plantcare/cgi-bin/show_site_info.htpl?QWhere=ID_of_Site%20like%20%27TATATA%27&StartAt=0&NbRecs=10) |  |  |
| SbSPL3 | 621 | 6 | TATATA | [AT~TATA-box](http://bioinformatics.psb.ugent.be/webtools/plantcare/cgi-bin/show_site_info.htpl?QWhere=ID_of_Site%20like%20%27TATATA%27&StartAt=0&NbRecs=10) |  |  |
| SbSPL3 | 599 | 6 | TATATA | [AT~TATA-box](http://bioinformatics.psb.ugent.be/webtools/plantcare/cgi-bin/show_site_info.htpl?QWhere=ID_of_Site%20like%20%27TATATA%27&StartAt=0&NbRecs=10) |  |  |
| SbSPL3 | 597 | 6 | TATATA | [AT~TATA-box](http://bioinformatics.psb.ugent.be/webtools/plantcare/cgi-bin/show_site_info.htpl?QWhere=ID_of_Site%20like%20%27TATATA%27&StartAt=0&NbRecs=10) |  |  |
| SbSPL3 | 603 | 6 | TATATA | [AT~TATA-box](http://bioinformatics.psb.ugent.be/webtools/plantcare/cgi-bin/show_site_info.htpl?QWhere=ID_of_Site%20like%20%27TATATA%27&StartAt=0&NbRecs=10) |  |  |
| SbSPL3 | 601 | 6 | TATATA | [AT~TATA-box](http://bioinformatics.psb.ugent.be/webtools/plantcare/cgi-bin/show_site_info.htpl?QWhere=ID_of_Site%20like%20%27TATATA%27&StartAt=0&NbRecs=10) |  |  |
| SbSPL3 | 595 | 6 | TATATA | [AT~TATA-box](http://bioinformatics.psb.ugent.be/webtools/plantcare/cgi-bin/show_site_info.htpl?QWhere=ID_of_Site%20like%20%27TATATA%27&StartAt=0&NbRecs=10) |  |  |
| SbSPL3 | 593 | 6 | TATATA | [AT~TATA-box](http://bioinformatics.psb.ugent.be/webtools/plantcare/cgi-bin/show_site_info.htpl?QWhere=ID_of_Site%20like%20%27TATATA%27&StartAt=0&NbRecs=10) |  |  |
| SbSPL3 | 617 | 6 | TATATA | [AT~TATA-box](http://bioinformatics.psb.ugent.be/webtools/plantcare/cgi-bin/show_site_info.htpl?QWhere=ID_of_Site%20like%20%27TATATA%27&StartAt=0&NbRecs=10) |  |  |
| SbSPL3 | 607 | 6 | TATATA | [AT~TATA-box](http://bioinformatics.psb.ugent.be/webtools/plantcare/cgi-bin/show_site_info.htpl?QWhere=ID_of_Site%20like%20%27TATATA%27&StartAt=0&NbRecs=10) |  |  |
| SbSPL3 | 605 | 6 | TATATA | [AT~TATA-box](http://bioinformatics.psb.ugent.be/webtools/plantcare/cgi-bin/show_site_info.htpl?QWhere=ID_of_Site%20like%20%27TATATA%27&StartAt=0&NbRecs=10) |  |  |
| SbSPL3 | 611 | 6 | TATATA | [AT~TATA-box](http://bioinformatics.psb.ugent.be/webtools/plantcare/cgi-bin/show_site_info.htpl?QWhere=ID_of_Site%20like%20%27TATATA%27&StartAt=0&NbRecs=10) |  |  |
| SbSPL3 | 591 | 6 | TATATA | [AT~TATA-box](http://bioinformatics.psb.ugent.be/webtools/plantcare/cgi-bin/show_site_info.htpl?QWhere=ID_of_Site%20like%20%27TATATA%27&StartAt=0&NbRecs=10) |  |  |
| SbSPL3 | 609 | 6 | TATATA | [AT~TATA-box](http://bioinformatics.psb.ugent.be/webtools/plantcare/cgi-bin/show_site_info.htpl?QWhere=ID_of_Site%20like%20%27TATATA%27&StartAt=0&NbRecs=10) |  |  |
| SbSPL3 | 589 | 6 | TATATA | [AT~TATA-box](http://bioinformatics.psb.ugent.be/webtools/plantcare/cgi-bin/show_site_info.htpl?QWhere=ID_of_Site%20like%20%27TATATA%27&StartAt=0&NbRecs=10) |  |  |
| SbSPL3 | 585 | 6 | TATATA | [AT~TATA-box](http://bioinformatics.psb.ugent.be/webtools/plantcare/cgi-bin/show_site_info.htpl?QWhere=ID_of_Site%20like%20%27TATATA%27&StartAt=0&NbRecs=10) |  |  |
| SbSPL3 | 587 | 6 | TATATA | [AT~TATA-box](http://bioinformatics.psb.ugent.be/webtools/plantcare/cgi-bin/show_site_info.htpl?QWhere=ID_of_Site%20like%20%27TATATA%27&StartAt=0&NbRecs=10) |  |  |
| SbSPL3 | 583 | 6 | TATATA | [AT~TATA-box](http://bioinformatics.psb.ugent.be/webtools/plantcare/cgi-bin/show_site_info.htpl?QWhere=ID_of_Site%20like%20%27TATATA%27&StartAt=0&NbRecs=10) |  |  |
| SbSPL3 | 452 | 6 | TATATA | [AT~TATA-box](http://bioinformatics.psb.ugent.be/webtools/plantcare/cgi-bin/show_site_info.htpl?QWhere=ID_of_Site%20like%20%27TATATA%27&StartAt=0&NbRecs=10) |  |  |
| SbSPL3 | 234 | 6 | TATATA | [AT~TATA-box](http://bioinformatics.psb.ugent.be/webtools/plantcare/cgi-bin/show_site_info.htpl?QWhere=ID_of_Site%20like%20%27TATATA%27&StartAt=0&NbRecs=10) |  |  |
| SbSPL3 | 230 | 6 | TATATA | [AT~TATA-box](http://bioinformatics.psb.ugent.be/webtools/plantcare/cgi-bin/show_site_info.htpl?QWhere=ID_of_Site%20like%20%27TATATA%27&StartAt=0&NbRecs=10) |  |  |
| SbSPL3 | 228 | 6 | TATATA | [AT~TATA-box](http://bioinformatics.psb.ugent.be/webtools/plantcare/cgi-bin/show_site_info.htpl?QWhere=ID_of_Site%20like%20%27TATATA%27&StartAt=0&NbRecs=10) |  |  |
| SbSPL3 | 232 | 6 | TATATA | [AT~TATA-box](http://bioinformatics.psb.ugent.be/webtools/plantcare/cgi-bin/show_site_info.htpl?QWhere=ID_of_Site%20like%20%27TATATA%27&StartAt=0&NbRecs=10) |  |  |
| SbSPL3 | 226 | 6 | TATATA | [AT~TATA-box](http://bioinformatics.psb.ugent.be/webtools/plantcare/cgi-bin/show_site_info.htpl?QWhere=ID_of_Site%20like%20%27TATATA%27&StartAt=0&NbRecs=10) |  |  |
| SbSPL3 | 222 | 6 | TATATA | [AT~TATA-box](http://bioinformatics.psb.ugent.be/webtools/plantcare/cgi-bin/show_site_info.htpl?QWhere=ID_of_Site%20like%20%27TATATA%27&StartAt=0&NbRecs=10) |  |  |
| SbSPL3 | 220 | 6 | TATATA | [AT~TATA-box](http://bioinformatics.psb.ugent.be/webtools/plantcare/cgi-bin/show_site_info.htpl?QWhere=ID_of_Site%20like%20%27TATATA%27&StartAt=0&NbRecs=10) |  |  |
| SbSPL3 | 224 | 6 | TATATA | [AT~TATA-box](http://bioinformatics.psb.ugent.be/webtools/plantcare/cgi-bin/show_site_info.htpl?QWhere=ID_of_Site%20like%20%27TATATA%27&StartAt=0&NbRecs=10) |  |  |
| SbSPL3 | 380 | 6 | ATTAAT | [Box 4](http://bioinformatics.psb.ugent.be/webtools/plantcare/cgi-bin/show_site_info.htpl?QWhere=ID_of_Site%20like%20%27ATTAAT%27&StartAt=0&NbRecs=10) | part of a conserved DNA module involved in light responsiveness | Light responsiveness |
| SbSPL3 | 865 | 6 | ATTAAT | [Box 4](http://bioinformatics.psb.ugent.be/webtools/plantcare/cgi-bin/show_site_info.htpl?QWhere=ID_of_Site%20like%20%27ATTAAT%27&StartAt=0&NbRecs=10) | part of a conserved DNA module involved in light responsiveness | Light responsiveness |
| SbSPL3 | 789 | 6 | ATTAAT | [Box 4](http://bioinformatics.psb.ugent.be/webtools/plantcare/cgi-bin/show_site_info.htpl?QWhere=ID_of_Site%20like%20%27ATTAAT%27&StartAt=0&NbRecs=10) | part of a conserved DNA module involved in light responsiveness | Light responsiveness |
| SbSPL3 | 1916 | 5 | CCAAT | [CAAT-box](http://bioinformatics.psb.ugent.be/webtools/plantcare/cgi-bin/show_site_info.htpl?QWhere=ID_of_Site%20like%20%27CCAAT%27&StartAt=0&NbRecs=10) | common cis-acting element in promoter and enhancer regions |  |
| SbSPL3 | 924 | 4 | CAAT | [CAAT-box](http://bioinformatics.psb.ugent.be/webtools/plantcare/cgi-bin/show_site_info.htpl?QWhere=ID_of_Site%20like%20%27CAAT%27&StartAt=0&NbRecs=10) |  |  |
| SbSPL3 | 956 | 4 | CAAT | [CAAT-box](http://bioinformatics.psb.ugent.be/webtools/plantcare/cgi-bin/show_site_info.htpl?QWhere=ID_of_Site%20like%20%27CAAT%27&StartAt=0&NbRecs=10) |  |  |
| SbSPL3 | 1392 | 4 | CAAT | [CAAT-box](http://bioinformatics.psb.ugent.be/webtools/plantcare/cgi-bin/show_site_info.htpl?QWhere=ID_of_Site%20like%20%27CAAT%27&StartAt=0&NbRecs=10) |  |  |
| SbSPL3 | 1289 | 5 | CAAAT | [CAAT-box](http://bioinformatics.psb.ugent.be/webtools/plantcare/cgi-bin/show_site_info.htpl?QWhere=ID_of_Site%20like%20%27CAAAT%27&StartAt=0&NbRecs=10) | common cis-acting element in promoter and enhancer regions |  |
| SbSPL3 | 655 | 5 | CCAAT | [CAAT-box](http://bioinformatics.psb.ugent.be/webtools/plantcare/cgi-bin/show_site_info.htpl?QWhere=ID_of_Site%20like%20%27CCAAT%27&StartAt=0&NbRecs=10) | common cis-acting element in promoter and enhancer regions |  |
| SbSPL3 | 1579 | 4 | CAAT | [CAAT-box](http://bioinformatics.psb.ugent.be/webtools/plantcare/cgi-bin/show_site_info.htpl?QWhere=ID_of_Site%20like%20%27CAAT%27&StartAt=0&NbRecs=10) |  |  |
| SbSPL3 | 440 | 4 | CAAT | [CAAT-box](http://bioinformatics.psb.ugent.be/webtools/plantcare/cgi-bin/show_site_info.htpl?QWhere=ID_of_Site%20like%20%27CAAT%27&StartAt=0&NbRecs=10) |  |  |
| SbSPL3 | 1828 | 4 | CAAT | [CAAT-box](http://bioinformatics.psb.ugent.be/webtools/plantcare/cgi-bin/show_site_info.htpl?QWhere=ID_of_Site%20like%20%27CAAT%27&StartAt=0&NbRecs=10) |  |  |
| SbSPL3 | 909 | 5 | CAAAT | [CAAT-box](http://bioinformatics.psb.ugent.be/webtools/plantcare/cgi-bin/show_site_info.htpl?QWhere=ID_of_Site%20like%20%27CAAAT%27&StartAt=0&NbRecs=10) | common cis-acting element in promoter and enhancer regions |  |
| SbSPL3 | 1461 | 4 | CAAT | [CAAT-box](http://bioinformatics.psb.ugent.be/webtools/plantcare/cgi-bin/show_site_info.htpl?QWhere=ID_of_Site%20like%20%27CAAT%27&StartAt=0&NbRecs=10) |  |  |
| SbSPL3 | 656 | 4 | CAAT | [CAAT-box](http://bioinformatics.psb.ugent.be/webtools/plantcare/cgi-bin/show_site_info.htpl?QWhere=ID_of_Site%20like%20%27CAAT%27&StartAt=0&NbRecs=10) |  |  |
| SbSPL3 | 1953 | 4 | CAAT | [CAAT-box](http://bioinformatics.psb.ugent.be/webtools/plantcare/cgi-bin/show_site_info.htpl?QWhere=ID_of_Site%20like%20%27CAAT%27&StartAt=0&NbRecs=10) |  |  |
| SbSPL3 | 1457 | 4 | CAAT | [CAAT-box](http://bioinformatics.psb.ugent.be/webtools/plantcare/cgi-bin/show_site_info.htpl?QWhere=ID_of_Site%20like%20%27CAAT%27&StartAt=0&NbRecs=10) |  |  |
| SbSPL3 | 916 | 5 | CAAAT | [CAAT-box](http://bioinformatics.psb.ugent.be/webtools/plantcare/cgi-bin/show_site_info.htpl?QWhere=ID_of_Site%20like%20%27CAAAT%27&StartAt=0&NbRecs=10) | common cis-acting element in promoter and enhancer regions |  |
| SbSPL3 | 1952 | 5 | CCAAT | [CAAT-box](http://bioinformatics.psb.ugent.be/webtools/plantcare/cgi-bin/show_site_info.htpl?QWhere=ID_of_Site%20like%20%27CCAAT%27&StartAt=0&NbRecs=10) | common cis-acting element in promoter and enhancer regions |  |
| SbSPL3 | 1122 | 4 | CAAT | [CAAT-box](http://bioinformatics.psb.ugent.be/webtools/plantcare/cgi-bin/show_site_info.htpl?QWhere=ID_of_Site%20like%20%27CAAT%27&StartAt=0&NbRecs=10) |  |  |
| SbSPL3 | 486 | 4 | CAAT | [CAAT-box](http://bioinformatics.psb.ugent.be/webtools/plantcare/cgi-bin/show_site_info.htpl?QWhere=ID_of_Site%20like%20%27CAAT%27&StartAt=0&NbRecs=10) |  |  |
| SbSPL3 | 1712 | 5 | CAAAT | [CAAT-box](http://bioinformatics.psb.ugent.be/webtools/plantcare/cgi-bin/show_site_info.htpl?QWhere=ID_of_Site%20like%20%27CAAAT%27&StartAt=0&NbRecs=10) | common cis-acting element in promoter and enhancer regions |  |
| SbSPL3 | 720 | 4 | CAAT | [CAAT-box](http://bioinformatics.psb.ugent.be/webtools/plantcare/cgi-bin/show_site_info.htpl?QWhere=ID_of_Site%20like%20%27CAAT%27&StartAt=0&NbRecs=10) |  |  |
| SbSPL3 | 1859 | 4 | CAAT | [CAAT-box](http://bioinformatics.psb.ugent.be/webtools/plantcare/cgi-bin/show_site_info.htpl?QWhere=ID_of_Site%20like%20%27CAAT%27&StartAt=0&NbRecs=10) |  |  |
| SbSPL3 | 1345 | 5 | CAAAT | [CAAT-box](http://bioinformatics.psb.ugent.be/webtools/plantcare/cgi-bin/show_site_info.htpl?QWhere=ID_of_Site%20like%20%27CAAAT%27&StartAt=0&NbRecs=10) | common cis-acting element in promoter and enhancer regions |  |
| SbSPL3 | 1544 | 4 | CAAT | [CAAT-box](http://bioinformatics.psb.ugent.be/webtools/plantcare/cgi-bin/show_site_info.htpl?QWhere=ID_of_Site%20like%20%27CAAT%27&StartAt=0&NbRecs=10) |  |  |
| SbSPL3 | 648 | 4 | CAAT | [CAAT-box](http://bioinformatics.psb.ugent.be/webtools/plantcare/cgi-bin/show_site_info.htpl?QWhere=ID_of_Site%20like%20%27CAAT%27&StartAt=0&NbRecs=10) |  |  |
| SbSPL3 | 1640 | 4 | CAAT | [CAAT-box](http://bioinformatics.psb.ugent.be/webtools/plantcare/cgi-bin/show_site_info.htpl?QWhere=ID_of_Site%20like%20%27CAAT%27&StartAt=0&NbRecs=10) |  |  |
| SbSPL3 | 1013 | 5 | CCAAT | [CAAT-box](http://bioinformatics.psb.ugent.be/webtools/plantcare/cgi-bin/show_site_info.htpl?QWhere=ID_of_Site%20like%20%27CCAAT%27&StartAt=0&NbRecs=10) | common cis-acting element in promoter and enhancer regions |  |
| SbSPL3 | 1917 | 4 | CAAT | [CAAT-box](http://bioinformatics.psb.ugent.be/webtools/plantcare/cgi-bin/show_site_info.htpl?QWhere=ID_of_Site%20like%20%27CAAT%27&StartAt=0&NbRecs=10) |  |  |
| SbSPL3 | 161 | 4 | CAAT | [CAAT-box](http://bioinformatics.psb.ugent.be/webtools/plantcare/cgi-bin/show_site_info.htpl?QWhere=ID_of_Site%20like%20%27CAAT%27&StartAt=0&NbRecs=10) |  |  |
| SbSPL3 | 547 | 4 | CAAT | [CAAT-box](http://bioinformatics.psb.ugent.be/webtools/plantcare/cgi-bin/show_site_info.htpl?QWhere=ID_of_Site%20like%20%27CAAT%27&StartAt=0&NbRecs=10) |  |  |
| SbSPL3 | 1418 | 5 | CAAAT | [CAAT-box](http://bioinformatics.psb.ugent.be/webtools/plantcare/cgi-bin/show_site_info.htpl?QWhere=ID_of_Site%20like%20%27CAAAT%27&StartAt=0&NbRecs=10) | common cis-acting element in promoter and enhancer regions |  |
| SbSPL3 | 460 | 4 | CAAT | [CAAT-box](http://bioinformatics.psb.ugent.be/webtools/plantcare/cgi-bin/show_site_info.htpl?QWhere=ID_of_Site%20like%20%27CAAT%27&StartAt=0&NbRecs=10) |  |  |
| SbSPL3 | 1996 | 4 | CAAT | [CAAT-box](http://bioinformatics.psb.ugent.be/webtools/plantcare/cgi-bin/show_site_info.htpl?QWhere=ID_of_Site%20like%20%27CAAT%27&StartAt=0&NbRecs=10) |  |  |
| SbSPL3 | 388 | 4 | CAAT | [CAAT-box](http://bioinformatics.psb.ugent.be/webtools/plantcare/cgi-bin/show_site_info.htpl?QWhere=ID_of_Site%20like%20%27CAAT%27&StartAt=0&NbRecs=10) |  |  |
| SbSPL3 | 1733 | 5 | CAAAT | [CAAT-box](http://bioinformatics.psb.ugent.be/webtools/plantcare/cgi-bin/show_site_info.htpl?QWhere=ID_of_Site%20like%20%27CAAAT%27&StartAt=0&NbRecs=10) | common cis-acting element in promoter and enhancer regions |  |
| SbSPL3 | 1421 | 4 | CAAT | [CAAT-box](http://bioinformatics.psb.ugent.be/webtools/plantcare/cgi-bin/show_site_info.htpl?QWhere=ID_of_Site%20like%20%27CAAT%27&StartAt=0&NbRecs=10) |  |  |
| SbSPL3 | 1053 | 6 | CCGTCC | [CCGTCC motif](http://bioinformatics.psb.ugent.be/webtools/plantcare/cgi-bin/show_site_info.htpl?QWhere=ID_of_Site%20like%20%27CCGTCC%27&StartAt=0&NbRecs=10) |  |  |
| SbSPL3 | 1053 | 6 | CCGTCC | [CCGTCC-box](http://bioinformatics.psb.ugent.be/webtools/plantcare/cgi-bin/show_site_info.htpl?QWhere=ID_of_Site%20like%20%27CCGTCC%27&StartAt=0&NbRecs=10) |  |  |
| SbSPL3 | 685 | 5 | CGTCA | [CGTCA-motif](http://bioinformatics.psb.ugent.be/webtools/plantcare/cgi-bin/show_site_info.htpl?QWhere=ID_of_Site%20like%20%27CGTCA%27&StartAt=0&NbRecs=10) | cis-acting regulatory element involved in the MeJA-responsiveness | Hormone responsiveness |
| SbSPL3 | 1219 | 5 | CGTCA | [CGTCA-motif](http://bioinformatics.psb.ugent.be/webtools/plantcare/cgi-bin/show_site_info.htpl?QWhere=ID_of_Site%20like%20%27CGTCA%27&StartAt=0&NbRecs=10) | cis-acting regulatory element involved in the MeJA-responsiveness | Hormone responsiveness |
| SbSPL3 | 1167 | 5 | CGTCA | [CGTCA-motif](http://bioinformatics.psb.ugent.be/webtools/plantcare/cgi-bin/show_site_info.htpl?QWhere=ID_of_Site%20like%20%27CGTCA%27&StartAt=0&NbRecs=10) | cis-acting regulatory element involved in the MeJA-responsiveness | Hormone responsiveness |
| SbSPL3 | 404 | 6 | CACGTT | [G-Box](http://bioinformatics.psb.ugent.be/webtools/plantcare/cgi-bin/show_site_info.htpl?QWhere=ID_of_Site%20like%20%27CACGTT%27&StartAt=0&NbRecs=10) | cis-acting regulatory element involved in light responsiveness |  |
| SbSPL3 | 752 | 6 | CACGTT | [G-Box](http://bioinformatics.psb.ugent.be/webtools/plantcare/cgi-bin/show_site_info.htpl?QWhere=ID_of_Site%20like%20%27CACGTT%27&StartAt=0&NbRecs=10) | cis-acting regulatory element involved in light responsiveness |  |
| SbSPL3 | 299 | 6 | CACGAC | [G-box](http://bioinformatics.psb.ugent.be/webtools/plantcare/cgi-bin/show_site_info.htpl?QWhere=ID_of_Site%20like%20%27CACGAC%27&StartAt=0&NbRecs=10) | cis-acting regulatory element involved in light responsiveness |  |
| SbSPL3 | 443 | 6 | GGTTAA | [GT1-motif](http://bioinformatics.psb.ugent.be/webtools/plantcare/cgi-bin/show_site_info.htpl?QWhere=ID_of_Site%20like%20%27GGTTAA%27&StartAt=0&NbRecs=10) | light responsive element | Light responsiveness |
| SbSPL3 | 442 | 7 | GGTTAAT | [GT1-motif](http://bioinformatics.psb.ugent.be/webtools/plantcare/cgi-bin/show_site_info.htpl?QWhere=ID_of_Site%20like%20%27GGTTAAT%27&StartAt=0&NbRecs=10) | light responsive element | Light responsiveness |
| SbSPL3 | 800 | 9 | gGATAAGGTG | [I-box](http://bioinformatics.psb.ugent.be/webtools/plantcare/cgi-bin/show_site_info.htpl?QWhere=ID_of_Site%20like%20%27gGATAAGGTG%27&StartAt=0&NbRecs=10) | part of a light responsive element | Light responsiveness |
| SbSPL3 | 1757 | 6 | CAACCA | [MYB](http://bioinformatics.psb.ugent.be/webtools/plantcare/cgi-bin/show_site_info.htpl?QWhere=ID_of_Site%20like%20%27CAACCA%27&StartAt=0&NbRecs=10) |  |  |
| SbSPL3 | 1761 | 6 | CAACCA | [MYB](http://bioinformatics.psb.ugent.be/webtools/plantcare/cgi-bin/show_site_info.htpl?QWhere=ID_of_Site%20like%20%27CAACCA%27&StartAt=0&NbRecs=10) |  |  |
| SbSPL3 | 1208 | 6 | TAACCA | [MYB](http://bioinformatics.psb.ugent.be/webtools/plantcare/cgi-bin/show_site_info.htpl?QWhere=ID_of_Site%20like%20%27TAACCA%27&StartAt=0&NbRecs=10) |  |  |
| SbSPL3 | 640 | 6 | CAACAG | [MYB](http://bioinformatics.psb.ugent.be/webtools/plantcare/cgi-bin/show_site_info.htpl?QWhere=ID_of_Site%20like%20%27CAACAG%27&StartAt=0&NbRecs=10) |  |  |
| SbSPL3 | 1892 | 6 | TAACCA | [MYB](http://bioinformatics.psb.ugent.be/webtools/plantcare/cgi-bin/show_site_info.htpl?QWhere=ID_of_Site%20like%20%27TAACCA%27&StartAt=0&NbRecs=10) |  |  |
| SbSPL3 | 64 | 6 | CAACCA | [MYB](http://bioinformatics.psb.ugent.be/webtools/plantcare/cgi-bin/show_site_info.htpl?QWhere=ID_of_Site%20like%20%27CAACCA%27&StartAt=0&NbRecs=10) |  |  |
| SbSPL3 | 1892 | 6 | TAACCA | [MYB-like sequence](http://bioinformatics.psb.ugent.be/webtools/plantcare/cgi-bin/show_site_info.htpl?QWhere=ID_of_Site%20like%20%27TAACCA%27&StartAt=0&NbRecs=10) |  |  |
| SbSPL3 | 1208 | 6 | TAACCA | [MYB-like sequence](http://bioinformatics.psb.ugent.be/webtools/plantcare/cgi-bin/show_site_info.htpl?QWhere=ID_of_Site%20like%20%27TAACCA%27&StartAt=0&NbRecs=10) |  |  |
| SbSPL3 | 908 | 6 | CATTTG | [MYC](http://bioinformatics.psb.ugent.be/webtools/plantcare/cgi-bin/show_site_info.htpl?QWhere=ID_of_Site%20like%20%27CATTTG%27&StartAt=0&NbRecs=10) |  |  |
| SbSPL3 | 268 | 6 | CATGTG | [MYC](http://bioinformatics.psb.ugent.be/webtools/plantcare/cgi-bin/show_site_info.htpl?QWhere=ID_of_Site%20like%20%27CATGTG%27&StartAt=0&NbRecs=10) |  |  |
| SbSPL3 | 1711 | 6 | CATTTG | [MYC](http://bioinformatics.psb.ugent.be/webtools/plantcare/cgi-bin/show_site_info.htpl?QWhere=ID_of_Site%20like%20%27CATTTG%27&StartAt=0&NbRecs=10) |  |  |
| SbSPL3 | 270 | 6 | CATGTG | [MYC](http://bioinformatics.psb.ugent.be/webtools/plantcare/cgi-bin/show_site_info.htpl?QWhere=ID_of_Site%20like%20%27CATGTG%27&StartAt=0&NbRecs=10) |  |  |
| SbSPL3 | 186 | 6 | TAACTG | [Myb](http://bioinformatics.psb.ugent.be/webtools/plantcare/cgi-bin/show_site_info.htpl?QWhere=ID_of_Site%20like%20%27TAACTG%27&StartAt=0&NbRecs=10) |  |  |
| SbSPL3 | 640 | 6 | CAACAG | [Myb-binding site](http://bioinformatics.psb.ugent.be/webtools/plantcare/cgi-bin/show_site_info.htpl?QWhere=ID_of_Site%20like%20%27CAACAG%27&StartAt=0&NbRecs=10) |  |  |
| SbSPL3 | 523 | 7 | TCTCTTA | [Myc](http://bioinformatics.psb.ugent.be/webtools/plantcare/cgi-bin/show_site_info.htpl?QWhere=ID_of_Site%20like%20%27TCTCTTA%27&StartAt=0&NbRecs=10) |  |  |
| SbSPL3 | 1074 | 5 | AGGGG | [STRE](http://bioinformatics.psb.ugent.be/webtools/plantcare/cgi-bin/show_site_info.htpl?QWhere=ID_of_Site%20like%20%27AGGGG%27&StartAt=0&NbRecs=10) |  |  |
| SbSPL3 | 31 | 5 | AGGGG | [STRE](http://bioinformatics.psb.ugent.be/webtools/plantcare/cgi-bin/show_site_info.htpl?QWhere=ID_of_Site%20like%20%27AGGGG%27&StartAt=0&NbRecs=10) |  |  |
| SbSPL3 | 609 | 6 | TATATA | [TATA-box](http://bioinformatics.psb.ugent.be/webtools/plantcare/cgi-bin/show_site_info.htpl?QWhere=ID_of_Site%20like%20%27TATATA%27&StartAt=0&NbRecs=10) | core promoter element around -30 of transcription start |  |
| SbSPL3 | 607 | 6 | TATATA | [TATA-box](http://bioinformatics.psb.ugent.be/webtools/plantcare/cgi-bin/show_site_info.htpl?QWhere=ID_of_Site%20like%20%27TATATA%27&StartAt=0&NbRecs=10) | core promoter element around -30 of transcription start |  |
| SbSPL3 | 1568 | 4 | TATA | [TATA-box](http://bioinformatics.psb.ugent.be/webtools/plantcare/cgi-bin/show_site_info.htpl?QWhere=ID_of_Site%20like%20%27TATA%27&StartAt=0&NbRecs=10) | core promoter element around -30 of transcription start |  |
| SbSPL3 | 786 | 4 | TATA | [TATA-box](http://bioinformatics.psb.ugent.be/webtools/plantcare/cgi-bin/show_site_info.htpl?QWhere=ID_of_Site%20like%20%27TATA%27&StartAt=0&NbRecs=10) | core promoter element around -30 of transcription start |  |
| SbSPL3 | 1663 | 4 | TATA | [TATA-box](http://bioinformatics.psb.ugent.be/webtools/plantcare/cgi-bin/show_site_info.htpl?QWhere=ID_of_Site%20like%20%27TATA%27&StartAt=0&NbRecs=10) | core promoter element around -30 of transcription start |  |
| SbSPL3 | 603 | 6 | TATATA | [TATA-box](http://bioinformatics.psb.ugent.be/webtools/plantcare/cgi-bin/show_site_info.htpl?QWhere=ID_of_Site%20like%20%27TATATA%27&StartAt=0&NbRecs=10) | core promoter element around -30 of transcription start |  |
| SbSPL3 | 1936 | 4 | TATA | [TATA-box](http://bioinformatics.psb.ugent.be/webtools/plantcare/cgi-bin/show_site_info.htpl?QWhere=ID_of_Site%20like%20%27TATA%27&StartAt=0&NbRecs=10) | core promoter element around -30 of transcription start |  |
| SbSPL3 | 1304 | 4 | TATA | [TATA-box](http://bioinformatics.psb.ugent.be/webtools/plantcare/cgi-bin/show_site_info.htpl?QWhere=ID_of_Site%20like%20%27TATA%27&StartAt=0&NbRecs=10) | core promoter element around -30 of transcription start |  |
| SbSPL3 | 599 | 6 | TATATA | [TATA-box](http://bioinformatics.psb.ugent.be/webtools/plantcare/cgi-bin/show_site_info.htpl?QWhere=ID_of_Site%20like%20%27TATATA%27&StartAt=0&NbRecs=10) | core promoter element around -30 of transcription start |  |
| SbSPL3 | 1303 | 5 | TATAA | [TATA-box](http://bioinformatics.psb.ugent.be/webtools/plantcare/cgi-bin/show_site_info.htpl?QWhere=ID_of_Site%20like%20%27TATAA%27&StartAt=0&NbRecs=10) | core promoter element around -30 of transcription start |  |
| SbSPL3 | 1567 | 5 | TATAA | [TATA-box](http://bioinformatics.psb.ugent.be/webtools/plantcare/cgi-bin/show_site_info.htpl?QWhere=ID_of_Site%20like%20%27TATAA%27&StartAt=0&NbRecs=10) | core promoter element around -30 of transcription start |  |
| SbSPL3 | 601 | 6 | TATATA | [TATA-box](http://bioinformatics.psb.ugent.be/webtools/plantcare/cgi-bin/show_site_info.htpl?QWhere=ID_of_Site%20like%20%27TATATA%27&StartAt=0&NbRecs=10) | core promoter element around -30 of transcription start |  |
| SbSPL3 | 1547 | 4 | TATA | [TATA-box](http://bioinformatics.psb.ugent.be/webtools/plantcare/cgi-bin/show_site_info.htpl?QWhere=ID_of_Site%20like%20%27TATA%27&StartAt=0&NbRecs=10) | core promoter element around -30 of transcription start |  |
| SbSPL3 | 623 | 4 | TATA | [TATA-box](http://bioinformatics.psb.ugent.be/webtools/plantcare/cgi-bin/show_site_info.htpl?QWhere=ID_of_Site%20like%20%27TATA%27&StartAt=0&NbRecs=10) | core promoter element around -30 of transcription start |  |
| SbSPL3 | 232 | 6 | TATATA | [TATA-box](http://bioinformatics.psb.ugent.be/webtools/plantcare/cgi-bin/show_site_info.htpl?QWhere=ID_of_Site%20like%20%27TATATA%27&StartAt=0&NbRecs=10) | core promoter element around -30 of transcription start |  |
| SbSPL3 | 621 | 6 | TATATA | [TATA-box](http://bioinformatics.psb.ugent.be/webtools/plantcare/cgi-bin/show_site_info.htpl?QWhere=ID_of_Site%20like%20%27TATATA%27&StartAt=0&NbRecs=10) | core promoter element around -30 of transcription start |  |
| SbSPL3 | 236 | 4 | TATA | [TATA-box](http://bioinformatics.psb.ugent.be/webtools/plantcare/cgi-bin/show_site_info.htpl?QWhere=ID_of_Site%20like%20%27TATA%27&StartAt=0&NbRecs=10) | core promoter element around -30 of transcription start |  |
| SbSPL3 | 200 | 4 | TATA | [TATA-box](http://bioinformatics.psb.ugent.be/webtools/plantcare/cgi-bin/show_site_info.htpl?QWhere=ID_of_Site%20like%20%27TATA%27&StartAt=0&NbRecs=10) | core promoter element around -30 of transcription start |  |
| SbSPL3 | 605 | 6 | TATATA | [TATA-box](http://bioinformatics.psb.ugent.be/webtools/plantcare/cgi-bin/show_site_info.htpl?QWhere=ID_of_Site%20like%20%27TATATA%27&StartAt=0&NbRecs=10) | core promoter element around -30 of transcription start |  |
| SbSPL3 | 589 | 6 | TATATA | [TATA-box](http://bioinformatics.psb.ugent.be/webtools/plantcare/cgi-bin/show_site_info.htpl?QWhere=ID_of_Site%20like%20%27TATATA%27&StartAt=0&NbRecs=10) | core promoter element around -30 of transcription start |  |
| SbSPL3 | 454 | 4 | TATA | [TATA-box](http://bioinformatics.psb.ugent.be/webtools/plantcare/cgi-bin/show_site_info.htpl?QWhere=ID_of_Site%20like%20%27TATA%27&StartAt=0&NbRecs=10) | core promoter element around -30 of transcription start |  |
| SbSPL3 | 45 | 4 | TATA | [TATA-box](http://bioinformatics.psb.ugent.be/webtools/plantcare/cgi-bin/show_site_info.htpl?QWhere=ID_of_Site%20like%20%27TATA%27&StartAt=0&NbRecs=10) | core promoter element around -30 of transcription start |  |
| SbSPL3 | 228 | 6 | TATATA | [TATA-box](http://bioinformatics.psb.ugent.be/webtools/plantcare/cgi-bin/show_site_info.htpl?QWhere=ID_of_Site%20like%20%27TATATA%27&StartAt=0&NbRecs=10) | core promoter element around -30 of transcription start |  |
| SbSPL3 | 615 | 6 | TATATA | [TATA-box](http://bioinformatics.psb.ugent.be/webtools/plantcare/cgi-bin/show_site_info.htpl?QWhere=ID_of_Site%20like%20%27TATATA%27&StartAt=0&NbRecs=10) | core promoter element around -30 of transcription start |  |
| SbSPL3 | 593 | 6 | TATATA | [TATA-box](http://bioinformatics.psb.ugent.be/webtools/plantcare/cgi-bin/show_site_info.htpl?QWhere=ID_of_Site%20like%20%27TATATA%27&StartAt=0&NbRecs=10) | core promoter element around -30 of transcription start |  |
| SbSPL3 | 1226 | 6 | ATTATA | [TATA-box](http://bioinformatics.psb.ugent.be/webtools/plantcare/cgi-bin/show_site_info.htpl?QWhere=ID_of_Site%20like%20%27ATTATA%27&StartAt=0&NbRecs=10) | core promoter element around -30 of transcription start |  |
| SbSPL3 | 453 | 6 | ATATAA | [TATA-box](http://bioinformatics.psb.ugent.be/webtools/plantcare/cgi-bin/show_site_info.htpl?QWhere=ID_of_Site%20like%20%27ATATAA%27&StartAt=0&NbRecs=10) | core promoter element around -30 of transcription start |  |
| SbSPL3 | 1723 | 5 | TATAA | [TATA-box](http://bioinformatics.psb.ugent.be/webtools/plantcare/cgi-bin/show_site_info.htpl?QWhere=ID_of_Site%20like%20%27TATAA%27&StartAt=0&NbRecs=10) | core promoter element around -30 of transcription start |  |
| SbSPL3 | 1724 | 4 | TATA | [TATA-box](http://bioinformatics.psb.ugent.be/webtools/plantcare/cgi-bin/show_site_info.htpl?QWhere=ID_of_Site%20like%20%27TATA%27&StartAt=0&NbRecs=10) | core promoter element around -30 of transcription start |  |
| SbSPL3 | 452 | 6 | TATATA | [TATA-box](http://bioinformatics.psb.ugent.be/webtools/plantcare/cgi-bin/show_site_info.htpl?QWhere=ID_of_Site%20like%20%27TATATA%27&StartAt=0&NbRecs=10) | core promoter element around -30 of transcription start |  |
| SbSPL3 | 220 | 6 | TATATA | [TATA-box](http://bioinformatics.psb.ugent.be/webtools/plantcare/cgi-bin/show_site_info.htpl?QWhere=ID_of_Site%20like%20%27TATATA%27&StartAt=0&NbRecs=10) | core promoter element around -30 of transcription start |  |
| SbSPL3 | 230 | 6 | TATATA | [TATA-box](http://bioinformatics.psb.ugent.be/webtools/plantcare/cgi-bin/show_site_info.htpl?QWhere=ID_of_Site%20like%20%27TATATA%27&StartAt=0&NbRecs=10) | core promoter element around -30 of transcription start |  |
| SbSPL3 | 604 | 6 | ATATAT | [TATA-box](http://bioinformatics.psb.ugent.be/webtools/plantcare/cgi-bin/show_site_info.htpl?QWhere=ID_of_Site%20like%20%27ATATAT%27&StartAt=0&NbRecs=10) | core promoter element around -30 of transcription start |  |
| SbSPL3 | 591 | 6 | TATATA | [TATA-box](http://bioinformatics.psb.ugent.be/webtools/plantcare/cgi-bin/show_site_info.htpl?QWhere=ID_of_Site%20like%20%27TATATA%27&StartAt=0&NbRecs=10) | core promoter element around -30 of transcription start |  |
| SbSPL3 | 1935 | 5 | TATAA | [TATA-box](http://bioinformatics.psb.ugent.be/webtools/plantcare/cgi-bin/show_site_info.htpl?QWhere=ID_of_Site%20like%20%27TATAA%27&StartAt=0&NbRecs=10) | core promoter element around -30 of transcription start |  |
| SbSPL3 | 198 | 6 | TATAAA | [TATA-box](http://bioinformatics.psb.ugent.be/webtools/plantcare/cgi-bin/show_site_info.htpl?QWhere=ID_of_Site%20like%20%27TATAAA%27&StartAt=0&NbRecs=10) | core promoter element around -30 of transcription start |  |
| SbSPL3 | 611 | 6 | TATATA | [TATA-box](http://bioinformatics.psb.ugent.be/webtools/plantcare/cgi-bin/show_site_info.htpl?QWhere=ID_of_Site%20like%20%27TATATA%27&StartAt=0&NbRecs=10) | core promoter element around -30 of transcription start |  |
| SbSPL3 | 585 | 6 | TATATA | [TATA-box](http://bioinformatics.psb.ugent.be/webtools/plantcare/cgi-bin/show_site_info.htpl?QWhere=ID_of_Site%20like%20%27TATATA%27&StartAt=0&NbRecs=10) | core promoter element around -30 of transcription start |  |
| SbSPL3 | 370 | 4 | TATA | [TATA-box](http://bioinformatics.psb.ugent.be/webtools/plantcare/cgi-bin/show_site_info.htpl?QWhere=ID_of_Site%20like%20%27TATA%27&StartAt=0&NbRecs=10) | core promoter element around -30 of transcription start |  |
| SbSPL3 | 610 | 6 | ATATAT | [TATA-box](http://bioinformatics.psb.ugent.be/webtools/plantcare/cgi-bin/show_site_info.htpl?QWhere=ID_of_Site%20like%20%27ATATAT%27&StartAt=0&NbRecs=10) | core promoter element around -30 of transcription start |  |
| SbSPL3 | 613 | 6 | TATATA | [TATA-box](http://bioinformatics.psb.ugent.be/webtools/plantcare/cgi-bin/show_site_info.htpl?QWhere=ID_of_Site%20like%20%27TATATA%27&StartAt=0&NbRecs=10) | core promoter element around -30 of transcription start |  |
| SbSPL3 | 234 | 6 | TATATA | [TATA-box](http://bioinformatics.psb.ugent.be/webtools/plantcare/cgi-bin/show_site_info.htpl?QWhere=ID_of_Site%20like%20%27TATATA%27&StartAt=0&NbRecs=10) | core promoter element around -30 of transcription start |  |
| SbSPL3 | 224 | 6 | TATATA | [TATA-box](http://bioinformatics.psb.ugent.be/webtools/plantcare/cgi-bin/show_site_info.htpl?QWhere=ID_of_Site%20like%20%27TATATA%27&StartAt=0&NbRecs=10) | core promoter element around -30 of transcription start |  |
| SbSPL3 | 1175 | 6 | ATATAT | [TATA-box](http://bioinformatics.psb.ugent.be/webtools/plantcare/cgi-bin/show_site_info.htpl?QWhere=ID_of_Site%20like%20%27ATATAT%27&StartAt=0&NbRecs=10) | core promoter element around -30 of transcription start |  |
| SbSPL3 | 706 | 4 | TATA | [TATA-box](http://bioinformatics.psb.ugent.be/webtools/plantcare/cgi-bin/show_site_info.htpl?QWhere=ID_of_Site%20like%20%27TATA%27&StartAt=0&NbRecs=10) | core promoter element around -30 of transcription start |  |
| SbSPL3 | 1176 | 4 | TATA | [TATA-box](http://bioinformatics.psb.ugent.be/webtools/plantcare/cgi-bin/show_site_info.htpl?QWhere=ID_of_Site%20like%20%27TATA%27&StartAt=0&NbRecs=10) | core promoter element around -30 of transcription start |  |
| SbSPL3 | 598 | 6 | ATATAT | [TATA-box](http://bioinformatics.psb.ugent.be/webtools/plantcare/cgi-bin/show_site_info.htpl?QWhere=ID_of_Site%20like%20%27ATATAT%27&StartAt=0&NbRecs=10) | core promoter element around -30 of transcription start |  |
| SbSPL3 | 597 | 6 | TATATA | [TATA-box](http://bioinformatics.psb.ugent.be/webtools/plantcare/cgi-bin/show_site_info.htpl?QWhere=ID_of_Site%20like%20%27TATATA%27&StartAt=0&NbRecs=10) | core promoter element around -30 of transcription start |  |
| SbSPL3 | 219 | 6 | ATATAT | [TATA-box](http://bioinformatics.psb.ugent.be/webtools/plantcare/cgi-bin/show_site_info.htpl?QWhere=ID_of_Site%20like%20%27ATATAT%27&StartAt=0&NbRecs=10) | core promoter element around -30 of transcription start |  |
| SbSPL3 | 1228 | 4 | TATA | [TATA-box](http://bioinformatics.psb.ugent.be/webtools/plantcare/cgi-bin/show_site_info.htpl?QWhere=ID_of_Site%20like%20%27TATA%27&StartAt=0&NbRecs=10) | core promoter element around -30 of transcription start |  |
| SbSPL3 | 226 | 6 | TATATA | [TATA-box](http://bioinformatics.psb.ugent.be/webtools/plantcare/cgi-bin/show_site_info.htpl?QWhere=ID_of_Site%20like%20%27TATATA%27&StartAt=0&NbRecs=10) | core promoter element around -30 of transcription start |  |
| SbSPL3 | 600 | 6 | ATATAT | [TATA-box](http://bioinformatics.psb.ugent.be/webtools/plantcare/cgi-bin/show_site_info.htpl?QWhere=ID_of_Site%20like%20%27ATATAT%27&StartAt=0&NbRecs=10) | core promoter element around -30 of transcription start |  |
| SbSPL3 | 587 | 6 | TATATA | [TATA-box](http://bioinformatics.psb.ugent.be/webtools/plantcare/cgi-bin/show_site_info.htpl?QWhere=ID_of_Site%20like%20%27TATATA%27&StartAt=0&NbRecs=10) | core promoter element around -30 of transcription start |  |
| SbSPL3 | 1722 | 6 | TATAAA | [TATA-box](http://bioinformatics.psb.ugent.be/webtools/plantcare/cgi-bin/show_site_info.htpl?QWhere=ID_of_Site%20like%20%27TATAAA%27&StartAt=0&NbRecs=10) | core promoter element around -30 of transcription start |  |
| SbSPL3 | 222 | 6 | TATATA | [TATA-box](http://bioinformatics.psb.ugent.be/webtools/plantcare/cgi-bin/show_site_info.htpl?QWhere=ID_of_Site%20like%20%27TATATA%27&StartAt=0&NbRecs=10) | core promoter element around -30 of transcription start |  |
| SbSPL3 | 595 | 6 | TATATA | [TATA-box](http://bioinformatics.psb.ugent.be/webtools/plantcare/cgi-bin/show_site_info.htpl?QWhere=ID_of_Site%20like%20%27TATATA%27&StartAt=0&NbRecs=10) | core promoter element around -30 of transcription start |  |
| SbSPL3 | 617 | 6 | TATATA | [TATA-box](http://bioinformatics.psb.ugent.be/webtools/plantcare/cgi-bin/show_site_info.htpl?QWhere=ID_of_Site%20like%20%27TATATA%27&StartAt=0&NbRecs=10) | core promoter element around -30 of transcription start |  |
| SbSPL3 | 250 | 6 | ATTATA | [TATA-box](http://bioinformatics.psb.ugent.be/webtools/plantcare/cgi-bin/show_site_info.htpl?QWhere=ID_of_Site%20like%20%27ATTATA%27&StartAt=0&NbRecs=10) | core promoter element around -30 of transcription start |  |
| SbSPL3 | 619 | 6 | TATATA | [TATA-box](http://bioinformatics.psb.ugent.be/webtools/plantcare/cgi-bin/show_site_info.htpl?QWhere=ID_of_Site%20like%20%27TATATA%27&StartAt=0&NbRecs=10) | core promoter element around -30 of transcription start |  |
| SbSPL3 | 583 | 6 | TATATA | [TATA-box](http://bioinformatics.psb.ugent.be/webtools/plantcare/cgi-bin/show_site_info.htpl?QWhere=ID_of_Site%20like%20%27TATATA%27&StartAt=0&NbRecs=10) | core promoter element around -30 of transcription start |  |
| SbSPL3 | 704 | 6 | ATTATA | [TATA-box](http://bioinformatics.psb.ugent.be/webtools/plantcare/cgi-bin/show_site_info.htpl?QWhere=ID_of_Site%20like%20%27ATTATA%27&StartAt=0&NbRecs=10) | core promoter element around -30 of transcription start |  |
| SbSPL3 | 199 | 5 | TATAA | [TATA-box](http://bioinformatics.psb.ugent.be/webtools/plantcare/cgi-bin/show_site_info.htpl?QWhere=ID_of_Site%20like%20%27TATAA%27&StartAt=0&NbRecs=10) | core promoter element around -30 of transcription start |  |
| SbSPL3 | 606 | 6 | ATATAT | [TATA-box](http://bioinformatics.psb.ugent.be/webtools/plantcare/cgi-bin/show_site_info.htpl?QWhere=ID_of_Site%20like%20%27ATATAT%27&StartAt=0&NbRecs=10) | core promoter element around -30 of transcription start |  |
| SbSPL3 | 251 | 5 | TATAA | [TATA-box](http://bioinformatics.psb.ugent.be/webtools/plantcare/cgi-bin/show_site_info.htpl?QWhere=ID_of_Site%20like%20%27TATAA%27&StartAt=0&NbRecs=10) | core promoter element around -30 of transcription start |  |
| SbSPL3 | 705 | 5 | TATAA | [TATA-box](http://bioinformatics.psb.ugent.be/webtools/plantcare/cgi-bin/show_site_info.htpl?QWhere=ID_of_Site%20like%20%27TATAA%27&StartAt=0&NbRecs=10) | core promoter element around -30 of transcription start |  |
| SbSPL3 | 231 | 6 | ATATAT | [TATA-box](http://bioinformatics.psb.ugent.be/webtools/plantcare/cgi-bin/show_site_info.htpl?QWhere=ID_of_Site%20like%20%27ATATAT%27&StartAt=0&NbRecs=10) | core promoter element around -30 of transcription start |  |
| SbSPL3 | 602 | 6 | ATATAT | [TATA-box](http://bioinformatics.psb.ugent.be/webtools/plantcare/cgi-bin/show_site_info.htpl?QWhere=ID_of_Site%20like%20%27ATATAT%27&StartAt=0&NbRecs=10) | core promoter element around -30 of transcription start |  |
| SbSPL3 | 592 | 6 | ATATAT | [TATA-box](http://bioinformatics.psb.ugent.be/webtools/plantcare/cgi-bin/show_site_info.htpl?QWhere=ID_of_Site%20like%20%27ATATAT%27&StartAt=0&NbRecs=10) | core promoter element around -30 of transcription start |  |
| SbSPL3 | 1302 | 6 | ATTATA | [TATA-box](http://bioinformatics.psb.ugent.be/webtools/plantcare/cgi-bin/show_site_info.htpl?QWhere=ID_of_Site%20like%20%27ATTATA%27&StartAt=0&NbRecs=10) | core promoter element around -30 of transcription start |  |
| SbSPL3 | 1279 | 4 | TATA | [TATA-box](http://bioinformatics.psb.ugent.be/webtools/plantcare/cgi-bin/show_site_info.htpl?QWhere=ID_of_Site%20like%20%27TATA%27&StartAt=0&NbRecs=10) | core promoter element around -30 of transcription start |  |
| SbSPL3 | 225 | 6 | ATATAT | [TATA-box](http://bioinformatics.psb.ugent.be/webtools/plantcare/cgi-bin/show_site_info.htpl?QWhere=ID_of_Site%20like%20%27ATATAT%27&StartAt=0&NbRecs=10) | core promoter element around -30 of transcription start |  |
| SbSPL3 | 252 | 4 | TATA | [TATA-box](http://bioinformatics.psb.ugent.be/webtools/plantcare/cgi-bin/show_site_info.htpl?QWhere=ID_of_Site%20like%20%27TATA%27&StartAt=0&NbRecs=10) | core promoter element around -30 of transcription start |  |
| SbSPL3 | 616 | 6 | ATATAT | [TATA-box](http://bioinformatics.psb.ugent.be/webtools/plantcare/cgi-bin/show_site_info.htpl?QWhere=ID_of_Site%20like%20%27ATATAT%27&StartAt=0&NbRecs=10) | core promoter element around -30 of transcription start |  |
| SbSPL3 | 233 | 6 | ATATAT | [TATA-box](http://bioinformatics.psb.ugent.be/webtools/plantcare/cgi-bin/show_site_info.htpl?QWhere=ID_of_Site%20like%20%27ATATAT%27&StartAt=0&NbRecs=10) | core promoter element around -30 of transcription start |  |
| SbSPL3 | 608 | 6 | ATATAT | [TATA-box](http://bioinformatics.psb.ugent.be/webtools/plantcare/cgi-bin/show_site_info.htpl?QWhere=ID_of_Site%20like%20%27ATATAT%27&StartAt=0&NbRecs=10) | core promoter element around -30 of transcription start |  |
| SbSPL3 | 1227 | 5 | TATAA | [TATA-box](http://bioinformatics.psb.ugent.be/webtools/plantcare/cgi-bin/show_site_info.htpl?QWhere=ID_of_Site%20like%20%27TATAA%27&StartAt=0&NbRecs=10) | core promoter element around -30 of transcription start |  |
| SbSPL3 | 588 | 6 | ATATAT | [TATA-box](http://bioinformatics.psb.ugent.be/webtools/plantcare/cgi-bin/show_site_info.htpl?QWhere=ID_of_Site%20like%20%27ATATAT%27&StartAt=0&NbRecs=10) | core promoter element around -30 of transcription start |  |
| SbSPL3 | 44 | 5 | TATAA | [TATA-box](http://bioinformatics.psb.ugent.be/webtools/plantcare/cgi-bin/show_site_info.htpl?QWhere=ID_of_Site%20like%20%27TATAA%27&StartAt=0&NbRecs=10) | core promoter element around -30 of transcription start |  |
| SbSPL3 | 612 | 6 | ATATAT | [TATA-box](http://bioinformatics.psb.ugent.be/webtools/plantcare/cgi-bin/show_site_info.htpl?QWhere=ID_of_Site%20like%20%27ATATAT%27&StartAt=0&NbRecs=10) | core promoter element around -30 of transcription start |  |
| SbSPL3 | 586 | 6 | ATATAT | [TATA-box](http://bioinformatics.psb.ugent.be/webtools/plantcare/cgi-bin/show_site_info.htpl?QWhere=ID_of_Site%20like%20%27ATATAT%27&StartAt=0&NbRecs=10) | core promoter element around -30 of transcription start |  |
| SbSPL3 | 622 | 6 | ATATAT | [TATA-box](http://bioinformatics.psb.ugent.be/webtools/plantcare/cgi-bin/show_site_info.htpl?QWhere=ID_of_Site%20like%20%27ATATAT%27&StartAt=0&NbRecs=10) | core promoter element around -30 of transcription start |  |
| SbSPL3 | 227 | 6 | ATATAT | [TATA-box](http://bioinformatics.psb.ugent.be/webtools/plantcare/cgi-bin/show_site_info.htpl?QWhere=ID_of_Site%20like%20%27ATATAT%27&StartAt=0&NbRecs=10) | core promoter element around -30 of transcription start |  |
| SbSPL3 | 614 | 6 | ATATAT | [TATA-box](http://bioinformatics.psb.ugent.be/webtools/plantcare/cgi-bin/show_site_info.htpl?QWhere=ID_of_Site%20like%20%27ATATAT%27&StartAt=0&NbRecs=10) | core promoter element around -30 of transcription start |  |
| SbSPL3 | 594 | 6 | ATATAT | [TATA-box](http://bioinformatics.psb.ugent.be/webtools/plantcare/cgi-bin/show_site_info.htpl?QWhere=ID_of_Site%20like%20%27ATATAT%27&StartAt=0&NbRecs=10) | core promoter element around -30 of transcription start |  |
| SbSPL3 | 618 | 6 | ATATAT | [TATA-box](http://bioinformatics.psb.ugent.be/webtools/plantcare/cgi-bin/show_site_info.htpl?QWhere=ID_of_Site%20like%20%27ATATAT%27&StartAt=0&NbRecs=10) | core promoter element around -30 of transcription start |  |
| SbSPL3 | 221 | 6 | ATATAT | [TATA-box](http://bioinformatics.psb.ugent.be/webtools/plantcare/cgi-bin/show_site_info.htpl?QWhere=ID_of_Site%20like%20%27ATATAT%27&StartAt=0&NbRecs=10) | core promoter element around -30 of transcription start |  |
| SbSPL3 | 620 | 6 | ATATAT | [TATA-box](http://bioinformatics.psb.ugent.be/webtools/plantcare/cgi-bin/show_site_info.htpl?QWhere=ID_of_Site%20like%20%27ATATAT%27&StartAt=0&NbRecs=10) | core promoter element around -30 of transcription start |  |
| SbSPL3 | 451 | 6 | ATATAT | [TATA-box](http://bioinformatics.psb.ugent.be/webtools/plantcare/cgi-bin/show_site_info.htpl?QWhere=ID_of_Site%20like%20%27ATATAT%27&StartAt=0&NbRecs=10) | core promoter element around -30 of transcription start |  |
| SbSPL3 | 235 | 6 | ATATAT | [TATA-box](http://bioinformatics.psb.ugent.be/webtools/plantcare/cgi-bin/show_site_info.htpl?QWhere=ID_of_Site%20like%20%27ATATAT%27&StartAt=0&NbRecs=10) | core promoter element around -30 of transcription start |  |
| SbSPL3 | 590 | 6 | ATATAT | [TATA-box](http://bioinformatics.psb.ugent.be/webtools/plantcare/cgi-bin/show_site_info.htpl?QWhere=ID_of_Site%20like%20%27ATATAT%27&StartAt=0&NbRecs=10) | core promoter element around -30 of transcription start |  |
| SbSPL3 | 223 | 6 | ATATAT | [TATA-box](http://bioinformatics.psb.ugent.be/webtools/plantcare/cgi-bin/show_site_info.htpl?QWhere=ID_of_Site%20like%20%27ATATAT%27&StartAt=0&NbRecs=10) | core promoter element around -30 of transcription start |  |
| SbSPL3 | 582 | 6 | ATATAT | [TATA-box](http://bioinformatics.psb.ugent.be/webtools/plantcare/cgi-bin/show_site_info.htpl?QWhere=ID_of_Site%20like%20%27ATATAT%27&StartAt=0&NbRecs=10) | core promoter element around -30 of transcription start |  |
| SbSPL3 | 229 | 6 | ATATAT | [TATA-box](http://bioinformatics.psb.ugent.be/webtools/plantcare/cgi-bin/show_site_info.htpl?QWhere=ID_of_Site%20like%20%27ATATAT%27&StartAt=0&NbRecs=10) | core promoter element around -30 of transcription start |  |
| SbSPL3 | 596 | 6 | ATATAT | [TATA-box](http://bioinformatics.psb.ugent.be/webtools/plantcare/cgi-bin/show_site_info.htpl?QWhere=ID_of_Site%20like%20%27ATATAT%27&StartAt=0&NbRecs=10) | core promoter element around -30 of transcription start |  |
| SbSPL3 | 584 | 6 | ATATAT | [TATA-box](http://bioinformatics.psb.ugent.be/webtools/plantcare/cgi-bin/show_site_info.htpl?QWhere=ID_of_Site%20like%20%27ATATAT%27&StartAt=0&NbRecs=10) | core promoter element around -30 of transcription start |  |
| SbSPL3 | 193 | 9 | TCATCTTCAT | [TCA](http://bioinformatics.psb.ugent.be/webtools/plantcare/cgi-bin/show_site_info.htpl?QWhere=ID_of_Site%20like%20%27TCATCTTCAT%27&StartAt=0&NbRecs=10) |  |  |
| SbSPL3 | 107 | 7 | TCTCCCT | [TCCC-motif](http://bioinformatics.psb.ugent.be/webtools/plantcare/cgi-bin/show_site_info.htpl?QWhere=ID_of_Site%20like%20%27TCTCCCT%27&StartAt=0&NbRecs=10) | part of a light responsive element |  |
| SbSPL3 | 1167 | 5 | TGACG | [TGACG-motif](http://bioinformatics.psb.ugent.be/webtools/plantcare/cgi-bin/show_site_info.htpl?QWhere=ID_of_Site%20like%20%27TGACG%27&StartAt=0&NbRecs=10) | cis-acting regulatory element involved in the MeJA-responsiveness | Hormone responsiveness |
| SbSPL3 | 1219 | 5 | TGACG | [TGACG-motif](http://bioinformatics.psb.ugent.be/webtools/plantcare/cgi-bin/show_site_info.htpl?QWhere=ID_of_Site%20like%20%27TGACG%27&StartAt=0&NbRecs=10) | cis-acting regulatory element involved in the MeJA-responsiveness | Hormone responsiveness |
| SbSPL3 | 685 | 5 | TGACG | [TGACG-motif](http://bioinformatics.psb.ugent.be/webtools/plantcare/cgi-bin/show_site_info.htpl?QWhere=ID_of_Site%20like%20%27TGACG%27&StartAt=0&NbRecs=10) | cis-acting regulatory element involved in the MeJA-responsiveness | Hormone responsiveness |
| SbSPL3 | 1921 | 9 | AACCTAACCT | [Unnamed__2](http://bioinformatics.psb.ugent.be/webtools/plantcare/cgi-bin/show_site_info.htpl?QWhere=ID_of_Site%20like%20%27AACCTAACCT%27&StartAt=0&NbRecs=10) |  |  |
| SbSPL3 | 109 | 4 | CTCC | [Unnamed__4](http://bioinformatics.psb.ugent.be/webtools/plantcare/cgi-bin/show_site_info.htpl?QWhere=ID_of_Site%20like%20%27CTCC%27&StartAt=0&NbRecs=10) |  |  |
| SbSPL3 | 478 | 4 | CTCC | [Unnamed__4](http://bioinformatics.psb.ugent.be/webtools/plantcare/cgi-bin/show_site_info.htpl?QWhere=ID_of_Site%20like%20%27CTCC%27&StartAt=0&NbRecs=10) |  |  |
| SbSPL3 | 12 | 4 | CTCC | [Unnamed__4](http://bioinformatics.psb.ugent.be/webtools/plantcare/cgi-bin/show_site_info.htpl?QWhere=ID_of_Site%20like%20%27CTCC%27&StartAt=0&NbRecs=10) |  |  |
| SbSPL3 | 1868 | 6 | CCACCT | [WRE3](http://bioinformatics.psb.ugent.be/webtools/plantcare/cgi-bin/show_site_info.htpl?QWhere=ID_of_Site%20like%20%27CCACCT%27&StartAt=0&NbRecs=10) |  |  |
| SbSPL3 | 1105 | 8 | AAATTACT | [WUN-motif](http://bioinformatics.psb.ugent.be/webtools/plantcare/cgi-bin/show_site_info.htpl?QWhere=ID_of_Site%20like%20%27AAATTACT%27&StartAt=0&NbRecs=10) |  |  |
| SbSPL3 | 1219 | 5 | TGACG | [as-1](http://bioinformatics.psb.ugent.be/webtools/plantcare/cgi-bin/show_site_info.htpl?QWhere=ID_of_Site%20like%20%27TGACG%27&StartAt=0&NbRecs=10) |  |  |
| SbSPL3 | 685 | 5 | TGACG | [as-1](http://bioinformatics.psb.ugent.be/webtools/plantcare/cgi-bin/show_site_info.htpl?QWhere=ID_of_Site%20like%20%27TGACG%27&StartAt=0&NbRecs=10) |  |  |
| SbSPL3 | 1167 | 5 | TGACG | [as-1](http://bioinformatics.psb.ugent.be/webtools/plantcare/cgi-bin/show_site_info.htpl?QWhere=ID_of_Site%20like%20%27TGACG%27&StartAt=0&NbRecs=10) |  |  |
| SbSPL4 | 681 | 5 | ACGTG | ABRE | cis-acting element involved in the abscisic acid responsiveness | Hormone responsiveness |
| SbSPL4 | 678 | 9 | GACACGTGGC | ABRE | cis-acting element involved in the abscisic acid responsiveness | Hormone responsiveness |
| SbSPL4 | 680 | 6 | CACGTG | ABRE | cis-acting element involved in the abscisic acid responsiveness | Hormone responsiveness |
| SbSPL4 | 219 | 8 | AGAAACTT |  | AE-box | Light responsiveness |
| SbSPL4 | 1554 | 6 | AAACCA | ARE | cis-acting regulatory element essential for the anaerobic induction | Abioticstress |
| SbSPL4 | 1864 | 6 | AAACCA | ARE | cis-acting regulatory element essential for the anaerobic induction | Abioticstress |
| SbSPL4 | 481 | 10 | ATAGAAATCAA |  | AT-rich element | Promoter-related |
| SbSPL4 | 120 | 10 | ATAGAAATCAA | AT-rich element | binding site of AT-rich DNA binding protein (ATBP-1) | Promoter-related |
| SbSPL4 | 1987 | 10 | ATAGAAATCAA | AT-rich element | binding site of AT-rich DNA binding protein (ATBP-1) | Promoter-related |
| SbSPL4 | 156 | 6 | ATTAAT | Box 4 | part of a conserved DNA module involved in light responsiveness | Light responsiveness |
| SbSPL4 | 423 | 6 | ATTAAT | Box 4 | part of a conserved DNA module involved in light responsiveness | Light responsiveness |
| SbSPL4 | 1027 | 6 | ATTAAT | Box 4 | part of a conserved DNA module involved in light responsiveness | Light responsiveness |
| SbSPL4 | 1916 | 6 | ATTAAT | Box 4 | part of a conserved DNA module involved in light responsiveness | Light responsiveness |
| SbSPL4 | 1638 | 6 | GCCACT | CAT-box | cis-acting regulatory element related to meristem expression | Development |
| SbSPL4 | 701 | 5 | CGTCA | CGTCA-motif | cis-acting regulatory element involved in the MeJA-responsiveness | Hormone responsiveness |
| SbSPL4 | 1818 | 5 | CGTCA | CGTCA-motif | cis-acting regulatory element involved in the MeJA-responsiveness | Hormone responsiveness |
| SbSPL4 | 680 | 6 | CACGTG | G-Box | cis-acting regulatory element involved in light responsiveness | Light responsiveness |
| SbSPL4 | 680 | 6 | CACGTG | G-box | cis-acting regulatory element involved in light responsiveness | Light responsiveness |
| SbSPL4 | 678 | 9 | GCCACGTGGA | G-box | cis-acting regulatory element involved in light responsiveness | Light responsiveness |
| SbSPL4 | 141 | 6 | GGTTAA | GT1-motif | light responsive element | Light responsiveness |
| SbSPL4 | 109 | 6 | GGTTAA | GT1-motif | light responsive element | Light responsiveness |
| SbSPL4 | 1063 | 9.5 | GTAAT(G/C)ATTAC | HD-Zip 3 | protein binding site | Development |
| SbSPL4 | 1046 | 8 | CTTTATCA | LAMP-element | part of a light responsive element | Light responsiveness |
| SbSPL4 | 142 | 6 | TAACCA | MYB | / | Abioticstress |
| SbSPL4 | 142 | 6 | TAACCA | MYB-like sequence | / | Development |
| SbSPL4 | 1944 | 6 | CATGTG | MYC | / | Abioticstress |
| SbSPL4 | 117 | 6 | CATTTG | MYC | / | Abioticstress |
| SbSPL4 | 1429 | 6 | CATGTG | MYC | / | Abioticstress |
| SbSPL4 | 328 | 6 | CATGTG | MYC | / | Abioticstress |
| SbSPL4 | 1751 | 6 | CATTTG | MYC | / | Abioticstress |
| SbSPL4 | 1879 | 6 | TAACTG | Myb | / | Unknown function |
| SbSPL4 | 516 | 6 | TCTTAC | TCT-motif | part of a light responsive element | Light responsiveness |
| SbSPL4 | 1818 | 5 | TGACG | TGACG-motif | cis-acting regulatory element involved in the MeJA-responsiveness | Hormone responsiveness |
| SbSPL4 | 701 | 5 | TGACG | TGACG-motif | cis-acting regulatory element involved in the MeJA-responsiveness | Hormone responsiveness |
| SbSPL4 | 1986 | 9 | CAAAGATATC | circadian | cis-acting regulatory element involved in circadian control | Development |
| SbSPL5 | 870 | 7 | GAAAGAA | AAGAA-motif | / | Development |
| SbSPL5 | 518 | 5 | ACGTG | ABRE | cis-acting element involved in the abscisic acid responsiveness | Hormone responsiveness |
| SbSPL5 | 879 | 6 | AAACCA | ARE | cis-acting regulatory element essential for the anaerobic induction | Abioticstress |
| SbSPL5 | 1142 | 6 | AAACCA | ARE | cis-acting regulatory element essential for the anaerobic induction | Abioticstress |
| SbSPL5 | 1052 | 7 | GGTCCAT | AuxRR-core | cis-acting regulatory element involved in auxin responsiveness | Hormone responsiveness |
| SbSPL5 | 34 | 6 | ATTAAT | Box 4 | part of a conserved DNA module involved in light responsiveness | Light responsiveness |
| SbSPL5 | 1615 | 6 | ATTAAT | Box 4 | part of a conserved DNA module involved in light responsiveness | Light responsiveness |
| SbSPL5 | 1337 | 11 | atCATTTTCACt | Box III | protein binding site | Unknown function |
| SbSPL5 | 1535 | 6 | GCCACT | CAT-box | cis-acting regulatory element related to meristem expression | Development |
| SbSPL5 | 1041 | 5 | CGTCA | CGTCA-motif | cis-acting regulatory element involved in the MeJA-responsiveness | Hormone responsiveness |
| SbSPL5 | 1133 | 5 | CGTCA | CGTCA-motif | cis-acting regulatory element involved in the MeJA-responsiveness | Hormone responsiveness |
| SbSPL5 | 518 | 6 | CACGTT | G-Box | cis-acting regulatory element involved in light responsiveness | Light responsiveness |
| SbSPL5 | 1864 | 7 | TCTGTTG | GARE-motif | gibberellin-responsive element | Hormone responsiveness |
| SbSPL5 | 708 | 7 | GGTTAAT | GT1-motif | light responsive element | Light responsiveness |
| SbSPL5 | 668 | 6 | GGTTAA | GT1-motif | light responsive element | Light responsiveness |
| SbSPL5 | 259 | 7 | AACCTAA | MRE | MYB binding site involved in light responsiveness | Light responsiveness |
| SbSPL5 | 475 | 7 | AACCTAA | MRE | MYB binding site involved in light responsiveness | Light responsiveness |
| SbSPL5 | 590 | 7 | AACCTAA | MRE | MYB binding site involved in light responsiveness | Light responsiveness |
| SbSPL5 | 669 | 6 | TAACCA | MYB | / | Abioticstress |
| SbSPL5 | 707 | 6 | TAACCA | MYB | / | Abioticstress |
| SbSPL5 | 227 | 6 | TAACCA | MYB | / | Abioticstress |
| SbSPL5 | 263 | 6 | TAACCA | MYB | / | Abioticstress |
| SbSPL5 | 1694 | 6 | CAACCA | MYB | / | Abioticstress |
| SbSPL5 | 1694 | 6 | CAACCA | MYB-like sequence | / | Development |
| SbSPL5 | 707 | 6 | TAACCA | MYB-like sequence | / | Development |
| SbSPL5 | 227 | 6 | TAACCA | MYB-like sequence | / | Development |
| SbSPL5 | 669 | 6 | TAACCA | MYB-like sequence | / | Development |
| SbSPL5 | 263 | 6 | TAACCA | MYC | / | Abioticstress |
| SbSPL5 | 1014 | 6 | CAATTG | MYC | / | Abioticstress |
| SbSPL5 | 1592 | 6 | CATTTG | MYC | / | Abioticstress |
| SbSPL5 | 1812 | 6 | CATTTG | MYC | / | Abioticstress |
| SbSPL5 | 1698 | 6 | CAATTG | MYC | / | Abioticstress |
| SbSPL5 | 1664 | 6 | TAACTG | Myb | / | Unknown function |
| SbSPL5 | 1865 | 6 | CAACAG | Myb-binding site | / | Abioticstress |
| SbSPL5 | 867 | 9 | GTTTTCTTAC | TC-rich repeats | cis-acting element involved in defense and stress responsiveness | Abioticstress |
| SbSPL5 | 1493 | 6 | TCTTAC | TCT-motif | part of a light responsive element | Light responsiveness |
| SbSPL5 | 510 | 6 | TCTTAC | TCT-motif | part of a light responsive element | Light responsiveness |
| SbSPL5 | 638 | 6 | AACGAC | TGA-element | auxin-responsive element | Hormone responsiveness |
| SbSPL5 | 436 | 6 | AACGAC | TGA-element | auxin-responsive element | Hormone responsiveness |
| SbSPL5 | 1133 | 5 | TGACG | TGACG-motif | cis-acting regulatory element involved in the MeJA-responsiveness | Hormone responsiveness |
| SbSPL5 | 1041 | 5 | TGACG | TGACG-motif | cis-acting regulatory element involved in the MeJA-responsiveness | Hormone responsiveness |
| SbSPL6 | 905 | 10 | TAAGAGAGGAA | 3-AF1 binding site | light responsive element | Light responsiveness |
| SbSPL6 | 1855 | 5 | ACGTG | ABRE | cis-acting element involved in the abscisic acid responsiveness | Hormone responsiveness |
| SbSPL6 | 1491 | 6 | AAACCA | ARE | cis-acting regulatory element essential for the anaerobic induction | Abioticstress |
| SbSPL6 | 944 | 6 | AAACCA | ARE | cis-acting regulatory element essential for the anaerobic induction | Abioticstress |
| SbSPL6 | 719 | 9 | TAAAATACT | AT-rich sequence | element for maximal elicitor-mediated activation (2copies) | Abioticstress |
| SbSPL6 | 818 | 8 | AGTAATCT | ATC-motif | part of a conserved DNA module involved in light responsiveness | Light responsiveness |
| SbSPL6 | 1714 | 6 | ATTAAT | Box 4 | part of a conserved DNA module involved in light responsiveness | Light responsiveness |
| SbSPL6 | 1570 | 6 | ATTAAT | Box 4 | part of a conserved DNA module involved in light responsiveness | Light responsiveness |
| SbSPL6 | 1507 | 6 | ATTAAT | Box 4 | part of a conserved DNA module involved in light responsiveness | Light responsiveness |
| SbSPL6 | 597 | 6 | ATTAAT | Box 4 | part of a conserved DNA module involved in light responsiveness | Light responsiveness |
| SbSPL6 | 1284 | 6 | GCCACT | CAT-box | cis-acting regulatory element related to meristem expression | Development |
| SbSPL6 | 1855 | 6 | CACGTC | G-box | cis-acting regulatory element involved in light responsiveness | Light responsiveness |
| SbSPL6 | 959 | 8 | CTTTATCA | LAMP-element | part of a light responsive element | Light responsiveness |
| SbSPL6 | 1747 | 6 | TAACCA | MYB | / | Abioticstress |
| SbSPL6 | 1487 | 6 | CAACCA | MYB | / | Abioticstress |
| SbSPL6 | 621 | 6 | CAACCA | MYB | / | Abioticstress |
| SbSPL6 | 489 | 6 | CAACCA | MYB | / | Abioticstress |
| SbSPL6 | 1747 | 6 | TAACCA | MYB-like sequence | MYB-like sequence | Development |
| SbSPL6 | 1277 | 6 | CATGTG | MYC | / | Abioticstress |
| SbSPL6 | 485 | 6 | CATTTG | MYC | / | Abioticstress |
| SbSPL6 | 116 | 6 | CATTTG | MYC | / | Abioticstress |
| SbSPL6 | 51 | 6 | CATGTG | MYC | / | Abioticstress |
| SbSPL6 | 1093 | 6 | TAACTG | Myb | / | Unknown function |
| SbSPL6 | 445 | 6 | TAACTG | Myb | / | Unknown function |
| SbSPL6 | 354 | 5 | AGGGG | STRE | / | Abioticstress |
| SbSPL6 | 1332 | 8 | TATAAAAT | TATA | / | Promoter-related |
| SbSPL6 | 389 | 7 | TCTCCCT | TCCC-motif | part of a light responsive element | Light responsiveness |
| SbSPL6 | 359 | 7 | TCTCCCT | TCCC-motif | part of a light responsive element | Light responsiveness |
| SbSPL6 | 613 | 6 | TCTTAC | TCT-motif | part of a light responsive element | Light responsiveness |
| SbSPL7 | 544 | 10 | TAAGAGAGGAA | 3-AF1 binding site | light responsive element | Light responsiveness |
| SbSPL7 | 931 | 6 | CACGTG | ABRE | cis-acting element involved in the abscisic acid responsiveness | Hormone responsiveness |
| SbSPL7 | 717 | 5 | ACGTG | ABRE | cis-acting element involved in the abscisic acid responsiveness | Hormone responsiveness |
| SbSPL7 | 1763 | 5 | ACGTG | ABRE | cis-acting element involved in the abscisic acid responsiveness | Hormone responsiveness |
| SbSPL7 | 932 | 5 | ACGTG | ABRE | cis-acting element involved in the abscisic acid responsiveness | Hormone responsiveness |
| SbSPL7 | 1516 | 6 | AAACCA | ARE | cis-acting regulatory element essential for the anaerobic induction | Abioticstress |
| SbSPL7 | 1503 | 6 | TATATA | AT-TATA-box | / | Promoter-related |
| SbSPL7 | 1994 | 6 | ATTAAT | Box 4 | part of a conserved DNA module involved in light responsiveness | Light responsiveness |
| SbSPL7 | 679 | 6 | ATTAAT | Box 4 | part of a conserved DNA module involved in light responsiveness | Light responsiveness |
| SbSPL7 | 1459 | 6 | ATTAAT | Box 4 | part of a conserved DNA module involved in light responsiveness | Light responsiveness |
| SbSPL7 | 1016 | 6 | ATTAAT | Box 4 | part of a conserved DNA module involved in light responsiveness | Light responsiveness |
| SbSPL7 | 1441 | 6 | ATTAAT | Box 4 | part of a conserved DNA module involved in light responsiveness | Light responsiveness |
| SbSPL7 | 1930 | 5 | CGTCA | CGTCA-motif | cis-acting regulatory element involved in the MeJA-responsiveness | Hormone responsiveness |
| SbSPL7 | 264 | 5 | CGTCA | CGTCA-motif | cis-acting regulatory element involved in the MeJA-responsiveness | Hormone responsiveness |
| SbSPL7 | 1765 | 5 | CGTCA | CGTCA-motif | cis-acting regulatory element involved in the MeJA-responsiveness | Hormone responsiveness |
| SbSPL7 | 250 | 9 | ACTAGCAGAA | CTAG-motif | / | Unknown function |
| SbSPL7 | 506 | 8 | TTTGCCGC | E2Fb | / | Unknown function |
| SbSPL7 | 1453 | 8 | ATTTCATA | ERE | / | Hormone responsiveness |
| SbSPL7 | 931 | 6 | CACGTG | G-Box | cis-acting regulatory element involved in light responsiveness | Light responsiveness |
| SbSPL7 | 716 | 6 | CACGTT | G-Box | cis-acting regulatory element involved in light responsiveness | Light responsiveness |
| SbSPL7 | 931 | 6 | CACGTG | G-box | cis-acting regulatory element involved in light responsiveness | Light responsiveness |
| SbSPL7 | 1763 | 6 | CACGTG | G-box | cis-acting regulatory element involved in light responsiveness | Light responsiveness |
| SbSPL7 | 1961 | 8 | ATAGATAA | GA-motif | part of a light responsive element | Light responsiveness |
| SbSPL7 | 1388 | 7 | TGAGTCA | GCN4_motif | cis-regulatory element involved in endosperm expression | Development |
| SbSPL7 | 1946 | 6 | CAACCA | MYB | / | Abioticstress |
| SbSPL7 | 1406 | 6 | CAACCA | MYB | / | Abioticstress |
| SbSPL7 | 608 | 6 | CATTTG | MYC | / | Abioticstress |
| SbSPL7 | 668 | 6 | CATGTG | MYC | / | Abioticstress |
| SbSPL7 | 1719 | 6 | CATGTG | MYC | / | Abioticstress |
| SbSPL7 | 1744 | 6 | CATGTG | MYC | / | Abioticstress |
| SbSPL7 | 548 | 7 | TCTCTTA | Myc | / | Unknown function |
| SbSPL7 | 1538 | 9 | TCATCTTCAT | TCA | / | Unknown function |
| SbSPL7 | 550 | 6 | TCTTAC | TCT-motif | part of a light responsive element | Light responsiveness |
| SbSPL7 | 727 | 6 | AACGAC | TGA-element | auxin-responsive element | Hormone responsiveness |
| SbSPL7 | 264 | 5 | TGACG | TGACG-motif | cis-acting regulatory element involved in the MeJA-responsiveness | Hormone responsiveness |
| SbSPL7 | 1930 | 5 | TGACG | TGACG-motif | cis-acting regulatory element involved in the MeJA-responsiveness | Hormone responsiveness |
| SbSPL7 | 1765 | 5 | TGACG | TGACG-motif | cis-acting regulatory element involved in the MeJA-responsiveness | Hormone responsiveness |
| SbSPL7 | 741 | 8 | TCACTTGA | chs-CMA2a | part of a light responsive element | Light responsiveness |
| SbSPL8 | 41 | 7 | GAAAGAA | AAGAA-motif | / | Development |
| SbSPL8 | 1768 | 7 | GAAAGAA | AAGAA-motif | / | Development |
| SbSPL8 | 1581 | 9 | gGTAAAGAAA | AAGAA-motif | / | Development |
| SbSPL8 | 808 | 6 | AAACCA | ARE | cis-acting regulatory element essential for the anaerobic induction | Abioticstress |
| SbSPL8 | 552 | 6 | ATTAAT | Box 4 | part of a conserved DNA module involved in light responsiveness | Light responsiveness |
| SbSPL8 | 1129 | 6 | ATTAAT | Box 4 | part of a conserved DNA module involved in light responsiveness | Light responsiveness |
| SbSPL8 | 1896 | 6 | ATTAAT | Box 4 | part of a conserved DNA module involved in light responsiveness | Light responsiveness |
| SbSPL8 | 392 | 6 | GCCACT | CAT-box | cis-acting regulatory element related to meristem expression | Development |
| SbSPL8 | 939 | 9 | ACTAGCAGAA | CTAG-motif | / | Unknown function |
| SbSPL8 | 1929 | 8 | ATTTTAAA | ERE | / | Hormone responsiveness |
| SbSPL8 | 1910 | 8 | ATTTTAAA | ERE | / | Hormone responsiveness |
| SbSPL8 | 486 | 7 | GATAGGA | GATA-motif | part of a light responsive element | Light responsiveness |
| SbSPL8 | 412 | 7 | GGTTAAT | GT1-motif | light responsive element | Light responsiveness |
| SbSPL8 | 1780 | 6 | GGTTAA | GT1-motif | light responsive element | Light responsiveness |
| SbSPL8 | 1787 | 6 | CCGAAA | LTR | cis-acting element involved in low-temperature responsiveness | Abioticstress |
| SbSPL8 | 1517 | 6 | CAACTG | MBS | MYB binding site involved in drought-inducibility | Abioticstress |
| SbSPL8 | 1982 | 6 | CAACTG | MBS | MYB binding site involved in drought-inducibility | Abioticstress |
| SbSPL8 | 1680 | 6 | CAACTG | MBS | MYB binding site involved in drought-inducibility | Abioticstress |
| SbSPL8 | 776 | 6 | CAACAG | MYB | / | Abioticstress |
| SbSPL8 | 331 | 6 | CATTTG | MYC | / | Abioticstress |
| SbSPL8 | 1468 | 6 | CAATTG | MYC | / | Abioticstress |
| SbSPL8 | 1610 | 6 | CATTTG | MYC | / | Abioticstress |
| SbSPL8 | 1148 | 6 | CATTTG | MYC | / | Abioticstress |
| SbSPL8 | 1072 | 6 | TAACTG | Myb | / | Unknown function |
| SbSPL8 | 1517 | 6 | CAACTG | Myb | / | Unknown function |
| SbSPL8 | 1680 | 6 | CAACTG | Myb | / | Unknown function |
| SbSPL8 | 1982 | 6 | CAACTG | Myb | / | Unknown function |
| SbSPL8 | 776 | 6 | CAACAG | Myb-binding site | / | Abioticstress |
| SbSPL8 | 851 | 7 | TCTCTTA | Myc | / | Unknown function |
| SbSPL8 | 843 | 8 | GATGA(C/T)(A/G)TG(A/G) | O2-site | cis-acting regulatory element involved in zein metabolism regulation | Development |
| SbSPL8 | 1951 | 7 | TATCCCA | TATC-box | cis-acting element involved in gibberellin-responsiveness | Hormone responsiveness |
| SbSPL8 | 825 | 9 | GTTTTCTTAC |  | TC-rich repeats | Abioticstress |
| SbSPL8 | 1455 | 9 | TCAGAAGAGG | TCA-element | cis-acting element involved in salicylic acid responsiveness | Hormone responsiveness |
| SbSPL8 | 825 | 6 | TCTTAC | TCT-motif | part of a light responsive element | Light responsiveness |
| SbSPL8 | 1390 | 6 | AACGAC | TGA-element | auxin-responsive element | Hormone responsiveness |
| SbSPL9 | 41 | 7 | GAAAGAA | AAGAA-motif | / | Development |
| SbSPL9 | 1768 | 7 | GAAAGAA | AAGAA-motif | / | Development |
| SbSPL9 | 1581 | 9 | gGTAAAGAAA | AAGAA-motif | / | Development |
| SbSPL9 | 808 | 6 | AAACCA | ARE | cis-acting regulatory element essential for the anaerobic induction | Abioticstress |
| SbSPL9 | 552 | 6 | ATTAAT | Box 4 | part of a conserved DNA module involved in light responsiveness | Light responsiveness |
| SbSPL9 | 1129 | 6 | ATTAAT | Box 4 | part of a conserved DNA module involved in light responsiveness | Light responsiveness |
| SbSPL9 | 1896 | 6 | ATTAAT | Box 4 | part of a conserved DNA module involved in light responsiveness | Light responsiveness |
| SbSPL9 | 392 | 6 | GCCACT | CAT-box | cis-acting regulatory element related to meristem expression | Development |
| SbSPL9 | 939 | 9 | ACTAGCAGAA | CTAG-motif | / | Unknown function |
| SbSPL9 | 486 | 7 | GATAGGA | GATA-motif | part of a light responsive element | Light responsiveness |
| SbSPL9 | 412 | 7 | GGTTAAT | GT1-motif | light responsive element | Light responsiveness |
| SbSPL9 | 1780 | 6 | GGTTAA | GT1-motif | light responsive element | Light responsiveness |
| SbSPL9 | 1787 | 6 | CCGAAA | LTR | cis-acting element involved in low-temperature responsiveness | Abioticstress |
| SbSPL9 | 1517 | 6 | CAACTG | MBS | MYB binding site involved in drought-inducibility | Abioticstress |
| SbSPL9 | 1982 | 6 | CAACTG | MBS | MYB binding site involved in drought-inducibility | Abioticstress |
| SbSPL9 | 1680 | 6 | CAACTG | MBS | MYB binding site involved in drought-inducibility | Abioticstress |
| SbSPL9 | 776 | 6 | CAACAG | MYB | / | Abioticstress |
| SbSPL9 | 331 | 6 | CATTTG | MYC | / | Abioticstress |
| SbSPL9 | 1468 | 6 | CAATTG | MYC | / | Abioticstress |
| SbSPL9 | 1610 | 6 | CATTTG | MYC | / | Abioticstress |
| SbSPL9 | 1148 | 6 | CATTTG | MYC | / | Abioticstress |
| SbSPL9 | 1072 | 6 | TAACTG | Myb | / | Unknown function |
| SbSPL9 | 1517 | 6 | CAACTG | Myb | / | Unknown function |
| SbSPL9 | 1680 | 6 | CAACTG | Myb | / | Unknown function |
| SbSPL9 | 1982 | 6 | CAACTG | Myb | / | Unknown function |
| SbSPL9 | 776 | 6 | CAACAG | Myb-binding site | / | Abioticstress |
| SbSPL9 | 851 | 7 | TCTCTTA | Myc | / | Unknown function |
| SbSPL9 | 843 | 8 | GATGA(C/T)(A/G)TG(A/G) | O2-site | / | Development |
| SbSPL9 | 1951 | 7 | TATCCCA | TATC-box | / | Hormone responsiveness |
| SbSPL9 | 825 | 9 | GTTTTCTTAC | TC-rich repeats | cis-acting element involved in defense and stress responsiveness | Abioticstress |
| SbSPL9 | 1445 | 9 | TCATCTTCAT | TCA | / | Unknown function |
| SbSPL9 | 1455 | 9 | TCAGAAGAGG | TCA-element | cis-acting element involved in salicylic acid responsiveness | Hormone responsiveness |
| SbSPL9 | 825 | 6 | TCTTAC | TCT-motif | part of a light responsive element | Light responsiveness |
| SbSPL9 | 1390 | 6 | AACGAC | TGA-element | auxin-responsive element | Hormone responsiveness |
| SbSPL10 | 1062 | 11 | CAATCAAAACCT | AAAC-motif | light responsive element | Light responsiveness |
| SbSPL10 | 1207 | 9 | TAAAATACT | AT-rich sequence | element for maximal elicitor-mediated activation (2copies) | Abioticstress |
| SbSPL10 | 414 | 13 | AATTATTTTTTATT | AT1-motif | part of a light responsive module | Light responsiveness |
| SbSPL10 | 254 | 6 | TATATA | AT-TATA-box | / | Promoter-related |
| SbSPL10 | 1349 | 6 | TATATA | AT-TATA-box | / | Promoter-related |
| SbSPL10 | 604 | 6 | ATTAAT | Box 4 | part of a conserved DNA module involved in light responsiveness | Light responsiveness |
| SbSPL10 | 1606 | 6 | ATTAAT | Box 4 | part of a conserved DNA module involved in light responsiveness | Light responsiveness |
| SbSPL10 | 1155 | 6 | ATTAAT | Box 4 | part of a conserved DNA module involved in light responsiveness | Light responsiveness |
| SbSPL10 | 1892 | 6 | GCCACT | CAT-box | cis-acting regulatory element related to meristem expression | Development |
| SbSPL10 | 83 | 5 | CGTCA | CGTCA-motif | cis-acting regulatory element involved in the MeJA-responsiveness | Hormone responsiveness |
| SbSPL10 | 1620 | 8 | ATTTCATA | ERE | / | Hormone responsiveness |
| SbSPL10 | 1620 | 8 | ATTTCATA | ERE | / | Hormone responsiveness |
| SbSPL10 | 1048 | 8 | ATTTTAAA | ERE | / | Hormone responsiveness |
| SbSPL10 | 823 | 8 | ATTTTAAA | ERE | / | Hormone responsiveness |
| SbSPL10 | 343 | 8 | ATTTTAAA | ERE | / | Hormone responsiveness |
| SbSPL10 | 623 | 8 | ATTTCATA | ERE | / | Hormone responsiveness |
| SbSPL10 | 163 | 6 | CACGAC | G-box | cis-acting regulatory element involved in light responsiveness | Light responsiveness |
| SbSPL10 | 281 | 8 | ATAGATAA | GA-motif | part of a light responsive element | Light responsiveness |
| SbSPL10 | 536 | 7 | GATAGGA | GATA-motif | part of a light responsive element | Light responsiveness |
| SbSPL10 | 502 | 6 | CCGAAA | LTR | cis-acting element involved in low-temperature responsiveness | Abioticstress |
| SbSPL10 | 453 | 6 | CAACTG | MBS | MYB binding site involved in drought-inducibility | Abioticstress |
| SbSPL10 | 1926 | 6 | CAACTG | MBS | MYB binding site involved in drought-inducibility | Abioticstress |
| SbSPL10 | 436 | 7 | AACCTAA | MRE | MYB binding site involved in light responsiveness | Light responsiveness |
| SbSPL10 | 1773 | 6 | CAACCA | MYB | / | Abioticstress |
| SbSPL10 | 1615 | 6 | CAACCA | MYB | / | Abioticstress |
| SbSPL10 | 1441 | 6 | CAACCA | MYB | / | Abioticstress |
| SbSPL10 | 977 | 6 | TAACCA | MYB | / | Abioticstress |
| SbSPL10 | 1279 | 6 | TAACCA | MYB | / | Abioticstress |
| SbSPL10 | 977 | 6 | TAACCA | MYB-like sequence | / | Development |
| SbSPL10 | 1844 | 6 | CAATTG | MYC | / | Abioticstress |
| SbSPL10 | 1664 | 6 | CAATTG | MYC | / | Abioticstress |
| SbSPL10 | 133 | 6 | CATTTG | MYC | / | Abioticstress |
| SbSPL10 | 1246 | 6 | CAATTG | MYC | / | Abioticstress |
| SbSPL10 | 1959 | 6 | TAACTG | Myb | / | Unknown function |
| SbSPL10 | 1926 | 6 | CAACTG | Myb | / | Unknown function |
| SbSPL10 | 453 | 6 | CAACTG | Myb | / | Unknown function |
| SbSPL10 | 978 | 7 | TCTCTTA | Myc | / | Unknown function |
| SbSPL10 | 945 | 9 | GATGATGTGG | O2-site | cis-acting regulatory element involved in zein metabolism regulation | Development |
| SbSPL10 | 10 | 7 | CCTTTTG | P-box | gibberellin-responsive element | Hormone responsiveness |
| SbSPL10 | 8 | 5 | AGGGG | STRE | / | Abioticstress |
| SbSPL10 | 1942 | 9 | ATTCTCTAAC | TC-rich repeats | cis-acting element involved in defense and stress responsiveness | Abioticstress |
| SbSPL10 | 766 | 9 | GTTTTCTTAC | TC-rich repeats | cis-acting element involved in defense and stress responsiveness | Abioticstress |
| SbSPL10 | 212 | 6 | TCTTAC | TCT-motif | part of a light responsive element | Light responsiveness |
| SbSPL10 | 1917 | 6 | AACGAC | TGA-element | auxin-responsive element | Hormone responsiveness |
| SbSPL10 | 83 | 5 | TGACG | TGACG-motif | cis-acting regulatory element involved in the MeJA-responsiveness | Hormone responsiveness |
| SbSPL10 | 1003 | 8 | TTACTTAA | chs-CMA1a | part of a light responsive element | Light responsiveness |
| SbSPL10 | 1822 | 9 | CAAAGATATC | circadian | cis-acting regulatory element involved in circadian control | Development |
| SbSPL11 | 1425 | 8 | AGTAATCT | ATC-motif | part of a conserved DNA module involved in light responsiveness | Light responsiveness |
| SbSPL11 | 38 | 7 | GGTCCAT | AuxRR-core | cis-acting regulatory element involved in auxin responsiveness | Hormone responsiveness |
| SbSPL11 | 1097 | 6 | ATTAAT | Box 4 | part of a conserved DNA module involved in light responsiveness | Light responsiveness |
| SbSPL11 | 1824 | 8 | ATTTTAAA | ERE | / | Hormone responsiveness |
| SbSPL11 | 1767 | 8 | ATTTTAAA | ERE | / | Hormone responsiveness |
| SbSPL11 | 1826 | 8 | ATTTTAAA | ERE | / | Hormone responsiveness |
| SbSPL11 | 1737 | 7 | GGTTAAT | GT1-motif | light responsive element | Light responsiveness |
| SbSPL11 | 866 | 7 | AACCTAA | MRE | MYB binding site involved in light responsiveness | Light responsiveness |
| SbSPL11 | 794 | 6 | CAATTG | MYC | / | Abioticstress |
| SbSPL11 | 230 | 6 | CATGTG | MYC | / | Abioticstress |
| SbSPL11 | 663 | 6 | TAACTG | Myb | / | Unknown function |
| SbSPL11 | 147 | 5 | AGGGG | STRE | / | Abioticstress |
| SbSPL11 | 1351 | 9 | CCATCTTTTT | TCA-element | cis-acting element involved in salicylic acid responsiveness | Hormone responsiveness |
| SbSPL11 | 977 | 6 | TCTTAC | TCT-motif | part of a light responsive element | Light responsiveness |
| SbSPL11 | 1805 | 6 | TCTTAC | TCT-motif | part of a light responsive element | Light responsiveness |
| SbSPL11 | 1034 | 9 | CAAAGATATC | circadian | cis-acting regulatory element involved in circadian control | Development |
| SbSPL12 | 889 | 7 | GAAAGAA | AAGAA-motif | / | Development |
| SbSPL12 | 1325 | 9 | gGTAAAGAAA | AAGAA-motif | / | Development |
| SbSPL12 | 217 | 5 | ACGTG | ABRE | cis-acting element involved in the abscisic acid responsiveness | Hormone responsiveness |
| SbSPL12 | 1076 | 5 | ACGTG | ABRE | cis-acting element involved in the abscisic acid responsiveness | Hormone responsiveness |
| SbSPL12 | 670 | 5 | ACGTG | ABRE | cis-acting element involved in the abscisic acid responsiveness | Hormone responsiveness |
| SbSPL12 | 216 | 6 | TACGTG | ABRE3a | / | Hormone responsiveness |
| SbSPL12 | 670 | 6 | TACGTG | ABRE3a | / | Hormone responsiveness |
| SbSPL12 | 216 | 6 | CACGTA | ABRE4 | / | Hormone responsiveness |
| SbSPL12 | 670 | 6 | CACGTA | ABRE4 | / | Hormone responsiveness |
| SbSPL12 | 738 | 8 | AGAAACAA | AE-box | part of a module for light response | Light responsiveness |
| SbSPL12 | 1231 | 6 | AAACCA | ARE | cis-acting regulatory element essential for the anaerobic induction | Abioticstress |
| SbSPL12 | 333 | 6 | TATATA | AT-TATA-box | / | Promoter-related |
| SbSPL12 | 478 | 6 | TATATA | AT-TATA-box | / | Promoter-related |
| SbSPL12 | 148 | 8 | TATATAAA | AT-TATA-box | / | Promoter-related |
| SbSPL12 | 1648 | 8 | TATATAAA | AT-TATA-box | / | Promoter-related |
| SbSPL12 | 331 | 6 | TATATA | AT-TATA-box | / | Promoter-related |
| SbSPL12 | 920 | 6 | TATATA | AT-TATA-box | / | Promoter-related |
| SbSPL12 | 476 | 6 | TATATA | AT-TATA-box | / | Promoter-related |
| SbSPL12 | 1650 | 6 | TATATA | AT-TATA-box | / | Promoter-related |
| SbSPL12 | 150 | 6 | TATATA | AT-TATA-box | / | Promoter-related |
| SbSPL12 | 152 | 6 | TATATA | AT-TATA-box | / | Promoter-related |
| SbSPL12 | 1366 | 6 | ATTAAT | Box 4 | part of a conserved DNA module involved in light responsiveness | Light responsiveness |
| SbSPL12 | 1913 | 6 | ATTAAT | Box 4 | part of a conserved DNA module involved in light responsiveness | Light responsiveness |
| SbSPL12 | 1738 | 5 | CGTCA | CGTCA-motif | cis-acting regulatory element involved in the MeJA-responsiveness | Hormone responsiveness |
| SbSPL12 | 1950 | 8 | ATTTTAAA | ERE | / | Hormone responsiveness |
| SbSPL12 | 1952 | 8 | ATTTTAAA | ERE | / | Hormone responsiveness |
| SbSPL12 | 1075 | 6 | CACGTT | G-Box | cis-acting regulatory element involved in light responsiveness | Light responsiveness |
| SbSPL12 | 216 | 6 | TACGTG | G-box | cis-acting regulatory element involved in light responsiveness | Light responsiveness |
| SbSPL12 | 1615 | 6 | CACGAC | G-box | cis-acting regulatory element involved in light responsiveness | Light responsiveness |
| SbSPL12 | 670 | 6 | TACGTG | G-box | cis-acting regulatory element involved in light responsiveness | Light responsiveness |
| SbSPL12 | 1812 | 6 | CAACTG | MBS | MYB binding site involved in drought-inducibility | Abioticstress |
| SbSPL12 | 1079 | 6 | CAACCA | MYB | / | Abioticstress |
| SbSPL12 | 1419 | 6 | TAACCA | MYB | / | Abioticstress |
| SbSPL12 | 1190 | 6 | CAACCA | MYB | / | Abioticstress |
| SbSPL12 | 1587 | 6 | CAACCA | MYB | / | Abioticstress |
| SbSPL12 | 1093 | 6 | CAACCA | MYB | / | Abioticstress |
| SbSPL12 | 1093 | 6 | CAACCA | MYB | / | Abioticstress |
| SbSPL12 | 1419 | 6 | TAACCA | MYB-like sequence | / | Development |
| SbSPL12 | 119 | 6 | CAATTG | MYC | / | Abioticstress |
| SbSPL12 | 1832 | 6 | CATGTG | MYC | / | Abioticstress |
| SbSPL12 | 1089 | 6 | CATGTG | MYC | / | Abioticstress |
| SbSPL12 | 397 | 6 | CAATTG | MYC | / | Abioticstress |
| SbSPL12 | 1289 | 6 | CATGTG | MYC | / | Abioticstress |
| SbSPL12 | 1812 | 6 | CAACTG | Myb | / | Unknown function |
| SbSPL12 | 375 | 5 | AGGGG | STRE | / | Abioticstress |
| SbSPL12 | 850 | 5 | AGGGG | STRE | / | Abioticstress |
| SbSPL12 | 520 | 5 | AGGGG | STRE | / | Abioticstress |
| SbSPL12 | 1778 | 5 | AGGGG | STRE | / | Abioticstress |
| SbSPL12 | 412 | 5 | AGGGG | STRE | / | Abioticstress |
| SbSPL12 | 556 | 5 | AGGGG | STRE | / | Abioticstress |
| SbSPL12 | 1137 | 5 | AGGGG | STRE | / | Abioticstress |
| SbSPL12 | 190 | 9 | TCAGAAGAGG | TCA-element | cis-acting element involved in salicylic acid responsiveness | Hormone responsiveness |
| SbSPL12 | 189 | 6 | TCTTAC | TCT-motif | part of a light responsive element | Light responsiveness |
| SbSPL12 | 1974 | 6 | TCTTAC | TCT-motif | part of a light responsive element | Light responsiveness |
| SbSPL12 | 1738 | 5 | TGACG | TGACG-motif | cis-acting regulatory element involved in the MeJA-responsiveness | Hormone responsiveness |
| SbSPL12 | 999 | 8 | TCACTTGA | chs-CMA2a | part of a light responsive element | Light responsiveness |
| SbSPL13 | 371 | 6 | CCGTCC | A-box | / | Promoter-related |
| SbSPL13 | 403 | 9 | (T/C)C(T/C)(C/T)ACC(T/C)ACC | AC-I | / | Unknown function |
| SbSPL13 | 450 | 6 | AAACCA | ARE | cis-acting regulatory element essential for the anaerobic induction | Abioticstress |
| SbSPL13 | 1522 | 6 | TATATA | AT-TATA-box | / | Promoter-related |
| SbSPL13 | 1307 | 6 | TATATA | AT-TATA-box | / | Promoter-related |
| SbSPL13 | 1307 | 6 | TATATA | AT-TATA-box | / | Promoter-related |
| SbSPL13 | 1084 | 6 | TATATA | AT-TATA-box | / | Promoter-related |
| SbSPL13 | 1082 | 6 | TATATA | AT-TATA-box | / | Promoter-related |
| SbSPL13 | 1080 | 8 | TATATAAA | AT-TATA-box | / | Promoter-related |
| SbSPL13 | 819 | 6 | TATATA | AT-TATA-box | / | Promoter-related |
| SbSPL13 | 1065 | 6 | TATATA | AT-TATA-box | / | Promoter-related |
| SbSPL13 | 750 | 6 | TATATA | AT-TATA-box | / | Promoter-related |
| SbSPL13 | 748 | 8 | TATATAAA |  | AT-TATA-box | Promoter-related |
| SbSPL13 | 1695 | 6 | ATTAAT | Box 4 | part of a conserved DNA module involved in light responsiveness | Light responsiveness |
| SbSPL13 | 1967 | 6 | CAACGG | CCAAT-box | MYBHv1 binding site | Abioticstress |
| SbSPL13 | 1967 | 6 | CAACGG | CCAAT-box | MYBHv1 binding site | Promoter-related |
| SbSPL13 | 371 | 6 | CCGTCC | CCGTCC motif | / | Unknown function |
| SbSPL13 | 371 | 6 | CCGTCC | CCGTCC-box | / | Development |
| SbSPL13 | 82 | 6 | GCCGAC | DRE core | / | Abioticstress |
| SbSPL13 | 1379 | 8 | ATTTTAAA | ERE | / | Hormone responsiveness |
| SbSPL13 | 884 | 8 | ATTTTAAA | ERE | / | Hormone responsiveness |
| SbSPL13 | 794 | 8 | ATTTTAAA | ERE | / | Hormone responsiveness |
| SbSPL13 | 288 | 10 | CTATTCTCATT | F-box | / | Development |
| SbSPL13 | 252 | 7 | TCTGTTG | GARE-motif | gibberellin-responsive element | Hormone responsiveness |
| SbSPL13 | 1242 | 10 | AAGATAAGATT | GATA-motif | part of a light responsive element | Light responsiveness |
| SbSPL13 | 1799 | 7 | TGAGTCA | GCN4_motif | cis-regulatory element involved in endosperm expression | Development |
| SbSPL13 | 1845 | 7 | GGTTAAT | GT1-motif | light responsive element | Light responsiveness |
| SbSPL13 | 216 | 7 | GGTTAAT | GT1-motif | light responsive element | Light responsiveness |
| SbSPL13 | 251 | 10 | GATTCTGTGGC | GTGGC-motif | part of a light responsive element | Light responsiveness |
| SbSPL13 | 974 | 6 | CAACTG | MBS | MYB binding site involved in drought-inducibility | Abioticstress |
| SbSPL13 | 757 | 6 | CAACTG | MBS | MYB binding site involved in drought-inducibility | Abioticstress |
| SbSPL13 | 778 | 6 | CAACCA | MYB | / | Abioticstress |
| SbSPL13 | 1844 | 6 | TAACCA | MYB | / | Abioticstress |
| SbSPL13 | 656 | 6 | TAACCA | MYB | / | Abioticstress |
| SbSPL13 | 252 | 6 | CAACAG | MYB | / | Abioticstress |
| SbSPL13 | 1967 | 6 | CCGTTG | MYB recognition site | / | Abioticstress |
| SbSPL13 | 1844 | 6 | TAACCA | MYB-like sequence | / | Development |
| SbSPL13 | 656 | 6 | TAACCA | MYB-like sequence | / | Development |
| SbSPL13 | 725 | 6 | CATTTG | MYC | / | Abioticstress |
| SbSPL13 | 143 | 6 | CAATTG | MYC | / | Abioticstress |
| SbSPL13 | 1778 | 6 | CATGTG | MYC | / | Abioticstress |
| SbSPL13 | 933 | 6 | CATTTG | MYC | / | Abioticstress |
| SbSPL13 | 1441 | 6 | TAACTG | Myb | / | Unknown function |
| SbSPL13 | 974 | 6 | CAACTG | Myb | / | Unknown function |
| SbSPL13 | 757 | 6 | CAACTG | Myb | / | Unknown function |
| SbSPL13 | 252 | 6 | CAACAG | Myb-binding site | / | Abioticstress |
| SbSPL13 | 430 | 5 | AGGGG | STRE | / | Abioticstress |
| SbSPL13 | 327 | 6 | GGGCGG | Sp1 | light responsive element | Light responsiveness |
| SbSPL13 | 601 | 8 | TATAAAAT | TATA | / | Promoter-related |
| SbSPL13 | 415 | 7 | TATCCCA | TATC-box | cis-acting element involved in gibberellin-responsiveness | Hormone responsiveness |
| SbSPL13 | 1261 | 9 |  | ATTCTCTAAC | TC-rich repeats | Abioticstress |
| SbSPL13 | 206 | 7 | TCTCCCT | TCCC-motif | part of a light responsive element | Light responsiveness |
| SbSPL13 | 807 | 6 | TCTTAC | TCT-motif | part of a light responsive element | Light responsiveness |
| SbSPL14 | 877 | 9 | GACACGTGGC | ABRE | cis-acting element involved in the abscisic acid responsiveness | Hormone responsiveness |
| SbSPL14 | 880 | 5 | ACGTG | ABRE | cis-acting element involved in the abscisic acid responsiveness | Hormone responsiveness |
| SbSPL14 | 879 | 6 | CACGTG | ABRE | cis-acting element involved in the abscisic acid responsiveness | Hormone responsiveness |
| SbSPL14 | 578 | 6 | AAACCA | ARE | cis-acting regulatory element essential for the anaerobic induction | Abioticstress |
| SbSPL14 | 1142 | 9 | TAAAATACT | AT-rich sequence | element for maximal elicitor-mediated activation (2copies) | Abioticstress |
| SbSPL14 | 1142 | 9 | TAAAATACT | AT-rich sequence | element for maximal elicitor-mediated activation (2copies) | Abioticstress |
| SbSPL14 | 328 | 6 | ATTAAT | Box 4 | part of a conserved DNA module involved in light responsiveness | Light responsiveness |
| SbSPL14 | 751 | 6 | ATTAAT | Box 4 | part of a conserved DNA module involved in light responsiveness | Light responsiveness |
| SbSPL14 | 928 | 7 | ACCGAGA | DRE1 | / | Abioticstress |
| SbSPL14 | 879 | 6 | CACGTG | G-Box | cis-acting regulatory element involved in light responsiveness | Light responsiveness |
| SbSPL14 | 878 | 9 | ACACGTGGC | G-box | cis-acting regulatory element involved in light responsiveness | Light responsiveness |
| SbSPL14 | 879 | 6 | CACGTG | G-box | cis-acting regulatory element involved in light responsiveness | Light responsiveness |
| SbSPL14 | 1668 | 7 | TGAGTCA | GCN4_motif | cis-regulatory element involved in endosperm expression | Development |
| SbSPL14 | 243 | 8 | CAAT(A/T)ATTG | HD-Zip 1 | element involved in differentiation of the palisade mesophyll cells | Development |
| SbSPL14 | 137 | 9 | GATAAGGGT | I-box | part of a light responsive element | Light responsiveness |
| SbSPL14 | 698 | 6 | CAACTG | MBS | MYB binding site involved in drought-inducibility | Abioticstress |
| SbSPL14 | 864 | 6 | TAACCA | MYB | / | Abioticstress |
| SbSPL14 | 864 | 6 | TAACCA | MYB-like sequence | / | Development |
| SbSPL14 | 1664 | 6 | CATTTG | MYC | / | Abioticstress |
| SbSPL14 | 1220 | 6 | CATTTG | MYC | / | Abioticstress |
| SbSPL14 | 726 | 6 | CATTTG | MYC | / | Abioticstress |
| SbSPL14 | 698 | 6 | CAACTG | Myb | / | Unknown function |
| SbSPL14 | 546 | 5 | AGGGG | STRE | / | Abioticstress |
| SbSPL14 | 369 | 9 | CCATCTTTTT | TCA-element | cis-acting element involved in salicylic acid responsiveness | Hormone responsiveness |
| SbSPL14 | 1343 | 6 | TCTTAC | TCT-motif | part of a light responsive element | Light responsiveness |
| SbSPL14 | 984 | 6 | AACGAC | TGA-element | auxin-responsive element | Hormone responsiveness |
| SbSPL14 | 1495 | 6 | AACGAC | TGA-element | auxin-responsive element | Hormone responsiveness |

| **Table S5: The Ka/Ks calculation of the duplicated SPL gene pairs in *S.baicalensis*** | | | | | | |
| --- | --- | --- | --- | --- | --- | --- |
| **Duplicated pairs** | **Gene replication modes** | **Ka** | **Ks** | **Ka/Ks** | **Mya** | **Purifying selection** |
| *SbSPL8-SbSPL9* | Proximal replication | 0.240380992 | 0.6833433 | 0.3518 | 52.5649 | Yes |
| *SbSPL4-SbSPL6* | Segmented replication | 0.266384025 | 0.679093893 | 0.3923 | 52.2380 | Yes |
| *SbSPL7-SbSPL10* | Segmented replication | 0.237910188 | 0.710076017 | 0.3350 | 54.6212 | Yes |
| *SbSPL1-SbSPL6* | Transposition replication | 1.001265162 | 1.277318916 | 0.7839 | 98.2553 | Yes |
| *SbSPL5-SbSPL6* | Transposition replication | 0.986877538 | 1.362013498 | 0.7246 | 104.7703 | Yes |
| *SbSPL3-SbSPL6* | Transposition replication | 0.827268654 | 1.295282366 | 0.6387 | 99.6371 | Yes |

| **Table S6 FPKM values of SbSPLs transcriptome sequencing** | | | | | |
| --- | --- | --- | --- | --- | --- |
| **Name** | **Gene Name** | **Root** | **Stem** | **Leaves** | **Flower** |
| GWHPBJEC001224 | SbSPL1 | 510.268 | 4.26595 | 0.415833 | 7.84465 |
| GWHPBJEC001236 | SbSPL2 | 3.21113 | 0.193952 | 0 | 0.0765916 |
| GWHPBJEC008370 | SbSPL4 | 0 | 0 | 0 | 0 |
| GWHPBJEC008863 | SbSPL5 | 0 | 0 | 0 | 0 |
| GWHPBJEC007369 | SbSPL3 | 8.61977 | 2.78123 | 2.40649 | 5.07701 |
| GWHPBJEC015511 | SbSPL6 | 0.0827764 | 0.225017 | 1.25915 | 8.89572 |
| GWHPBJEC021057 | SbSPL7 | 0 | 0 | 0 | 0 |
| GWHPBJEC022781 | SbSPL8 | 19.6527 | 7.96914 | 10.9372 | 11.6337 |
| GWHPBJEC022783 | SbSPL9 | 20.8112 | 37.83 | 22.041 | 32.1105 |
| GWHPBJEC025164 | SbSPL12 | 83.5025 | 27.5183 | 36.3216 | 41.2624 |
| GWHPBJEC024368 | SbSPL10 | 39.4401 | 243.712 | 99.4934 | 466.239 |
| GWHPBJEC024607 | SbSPL11 | 0 | 0 | 0 | 0 |
| GWHPBJEC027144 | SbSPL13 | 1.14261 | 84.9241 | 3.53284 | 25040.9 |
| GWHPBJEC029206 | SbSPL14 | 6.10278 | 3.96637 | 8.01682 | 8.01591 |

| **Table S7 Basic information of SPL gene family and target genes** | | | | | |  |  |  |  |  |  |  |
| --- | --- | --- | --- | --- | --- | --- | --- | --- | --- | --- | --- | --- |
| **SPL family gene locus Id** | **Self-named** | **Transcript Id** | **Protein Id** | **Target gene locus Id** | **Target gene Transcript Id** | **Target gene Protein Id** | **R Value** | **Regulation Model** | **KO number Corresponding To The Target Gene** | **Metabolic Pathways Matched by Target genes** | **Target gene K number** | **Homologous genes on Arabidopsis** |
| evm.model.contig168.577 | SbSPL2 | GWHTBJEC001262 | GWHPBJEC001236 | evm.model.contig515.180 | GWHTBJEC004979 | GWHPBJEC004859 | 0.867744372 | Plus | path:ko00940 | Phenylpropanoid biosynthesis | K09755 | CYP84A, F5H; ferulate-5-hydroxylase; [EC:1.14.-.-] |
| evm.model.contig539.9 | SbSPL3 | GWHTBJEC007527 | GWHPBJEC007369 | evm.model.contig515.180 | GWHTBJEC004979 | GWHPBJEC004859 | -0.51295663 | Minus | path:ko00940 | Phenylpropanoid biosynthesis | K09755 | CYP84A, F5H; ferulate-5-hydroxylase; [EC:1.14.-.-] |
| evm.model.contig269.130 | SbSPL6 | GWHTBJEC015825 | GWHPBJEC015511 | evm.model.contig515.180 | GWHTBJEC004979 | GWHPBJEC004859 | 0.724795305 | Plus | path:ko00940 | Phenylpropanoid biosynthesis | K09755 | CYP84A, F5H; ferulate-5-hydroxylase; [EC:1.14.-.-] |
| evm.model.contig168.577 | SbSPL2 | GWHTBJEC001262 | GWHPBJEC001236 | evm.model.contig522.188 | GWHTBJEC014099 | GWHPBJEC013833 | 0.793996087 | Plus | path:ko00940;path:ko00945;path:ko00941 | Phenylpropanoid biosynthesis\|Stilbenoid, diarylheptanoid and gingerol biosynthesis\|Flavonoid biosynthesis | K00588 | E2.1.1.104; caffeoyl-CoA O-methyltransferase; [EC:2.1.1.104] |
| evm.model.contig269.130 | SbSPL6 | GWHTBJEC015825 | GWHPBJEC015511 | evm.model.contig522.188 | GWHTBJEC014099 | GWHPBJEC013833 | 0.856543476 | Plus | path:ko00940;path:ko00945;path:ko00941 | Phenylpropanoid biosynthesis\|Stilbenoid, diarylheptanoid and gingerol biosynthesis\|Flavonoid biosynthesis | K00588 | E2.1.1.104; caffeoyl-CoA O-methyltransferase; [EC:2.1.1.104] |
| evm.model.contig168.565 | SbSPL1 | GWHTBJEC001250 | GWHPBJEC001224 | evm.model.contig575.1 | GWHTBJEC011156 | GWHPBJEC010935 | 0.80387343 | Plus | path:ko00940 | Phenylpropanoid biosynthesis | K00430 | E1.11.1.7; peroxidase; [EC:1.11.1.7] |
| evm.model.contig273.92 | SbSPL4 | GWHTBJEC008542 | GWHPBJEC008370 | evm.model.contig575.1 | GWHTBJEC011156 | GWHPBJEC010935 | 0.699109098 | Plus | path:ko00940 | Phenylpropanoid biosynthesis | K00430 | E1.11.1.7; peroxidase; [EC:1.11.1.7] |
| evm.model.contig370.303 | SbSPL5 | GWHTBJEC009037 | GWHPBJEC008863 | evm.model.contig575.1 | GWHTBJEC011156 | GWHPBJEC010935 | 0.671667535 | Plus | path:ko00940 | Phenylpropanoid biosynthesis | K00430 | E1.11.1.7; peroxidase; [EC:1.11.1.7] |
| evm.model.contig539.9 | SbSPL3 | GWHTBJEC007527 | GWHPBJEC007369 | evm.model.contig575.1 | GWHTBJEC011156 | GWHPBJEC010935 | -0.582751078 | Minus | path:ko00940 | Phenylpropanoid biosynthesis | K00430 | E1.11.1.7; peroxidase; [EC:1.11.1.7] |
| evm.model.contig464.49 | SbSPL7 | GWHTBJEC021508 | GWHPBJEC021057 | evm.model.contig575.1 | GWHTBJEC011156 | GWHPBJEC010935 | 0.718395367 | Plus | path:ko00940 | Phenylpropanoid biosynthesis | K00430 | E1.11.1.7; peroxidase; [EC:1.11.1.7] |
| evm.model.contig420.12 | SbSPL8 | GWHTBJEC023292 | GWHPBJEC022781 | evm.model.contig575.1 | GWHTBJEC011156 | GWHPBJEC010935 | 0.827103345 | Plus | path:ko00940 | Phenylpropanoid biosynthesis | K00430 | E1.11.1.7; peroxidase; [EC:1.11.1.7] |
| evm.model.contig420.14 | SbSPL9 | GWHTBJEC023294 | GWHPBJEC022783 | evm.model.contig575.1 | GWHTBJEC011156 | GWHPBJEC010935 | 0.79845875 | Plus | path:ko00940 | Phenylpropanoid biosynthesis | K00430 | E1.11.1.7; peroxidase; [EC:1.11.1.7] |
| evm.model.contig106.94 | SbSPL12 | GWHTBJEC025733 | GWHPBJEC025164 | evm.model.contig575.1 | GWHTBJEC011156 | GWHPBJEC010935 | -0.769453493 | Minus | path:ko00940 | Phenylpropanoid biosynthesis | K00430 | E1.11.1.7; peroxidase; [EC:1.11.1.7] |
| evm.model.contig155.11 | SbSPL10 | GWHTBJEC024923 | GWHPBJEC024368 | evm.model.contig575.1 | GWHTBJEC011156 | GWHPBJEC010935 | 0.801979633 | Plus | path:ko00940 | Phenylpropanoid biosynthesis | K00430 | E1.11.1.7; peroxidase; [EC:1.11.1.7] |
| evm.model.contig155.257 | SbSPL11 | GWHTBJEC025167 | GWHPBJEC024607 | evm.model.contig575.1 | GWHTBJEC011156 | GWHPBJEC010935 | 0.891661173 | Plus | path:ko00940 | Phenylpropanoid biosynthesis | K00430 | E1.11.1.7; peroxidase; [EC:1.11.1.7] |
| evm.model.contig455.712 | SbSPL14 | GWHTBJEC029854 | GWHPBJEC029206 | evm.model.contig575.1 | GWHTBJEC011156 | GWHPBJEC010935 | 0.648339371 | Plus | path:ko00940 | Phenylpropanoid biosynthesis | K00430 | E1.11.1.7; peroxidase; [EC:1.11.1.7] |
| evm.model.contig168.577 | SbSPL2 | GWHTBJEC001262 | GWHPBJEC001236 | evm.model.contig300.287 | GWHTBJEC027402 | GWHPBJEC026792 | 0.91870905 | Plus | path:ko00940 | Phenylpropanoid biosynthesis | K00430 | E1.11.1.7; peroxidase; [EC:1.11.1.7] |
| evm.model.contig539.9 | SbSPL3 | GWHTBJEC007527 | GWHPBJEC007369 | evm.model.contig300.287 | GWHTBJEC027402 | GWHPBJEC026792 | -0.56502338 | Minus | path:ko00940 | Phenylpropanoid biosynthesis | K00430 | E1.11.1.7; peroxidase; [EC:1.11.1.7] |
| evm.model.contig269.130 | SbSPL6 | GWHTBJEC015825 | GWHPBJEC015511 | evm.model.contig300.287 | GWHTBJEC027402 | GWHPBJEC026792 | 0.774114128 | Plus | path:ko00940 | Phenylpropanoid biosynthesis | K00430 | E1.11.1.7; peroxidase; [EC:1.11.1.7] |
| evm.model.contig420.12 | SbSPL8 | GWHTBJEC023292 | GWHPBJEC022781 | evm.model.contig300.287 | GWHTBJEC027402 | GWHPBJEC026792 | 0.534620894 | Plus | path:ko00940 | Phenylpropanoid biosynthesis | K00430 | E1.11.1.7; peroxidase; [EC:1.11.1.7] |
| evm.model.contig168.577 | SbSPL2 | GWHTBJEC001262 | GWHPBJEC001236 | evm.model.contig507.297 | GWHTBJEC028510 | GWHPBJEC027881 | 0.877958428 | Plus | path:ko00940 | Phenylpropanoid biosynthesis | K00430 | E1.11.1.7; peroxidase; [EC:1.11.1.7] |
| evm.model.contig370.303 | SbSPL5 | GWHTBJEC009037 | GWHPBJEC008863 | evm.model.contig507.297 | GWHTBJEC028510 | GWHPBJEC027881 | 0.631787266 | Plus | path:ko00940 | Phenylpropanoid biosynthesis | K00430 | E1.11.1.7; peroxidase; [EC:1.11.1.7] |
| evm.model.contig539.9 | SbSPL3 | GWHTBJEC007527 | GWHPBJEC007369 | evm.model.contig507.297 | GWHTBJEC028510 | GWHPBJEC027881 | -0.70656675 | Minus | path:ko00940 | Phenylpropanoid biosynthesis | K00430 | E1.11.1.7; peroxidase; [EC:1.11.1.7] |
| evm.model.contig269.130 | SbSPL6 | GWHTBJEC015825 | GWHPBJEC015511 | evm.model.contig507.297 | GWHTBJEC028510 | GWHPBJEC027881 | 0.600114564 | Plus | path:ko00940 | Phenylpropanoid biosynthesis | K00430 | E1.11.1.7; peroxidase; [EC:1.11.1.7] |
| evm.model.contig464.49 | SbSPL7 | GWHTBJEC021508 | GWHPBJEC021057 | evm.model.contig507.297 | GWHTBJEC028510 | GWHPBJEC027881 | 0.732165995 | Plus | path:ko00940 | Phenylpropanoid biosynthesis | K00430 | E1.11.1.7; peroxidase; [EC:1.11.1.7] |
| evm.model.contig420.12 | SbSPL8 | GWHTBJEC023292 | GWHPBJEC022781 | evm.model.contig507.297 | GWHTBJEC028510 | GWHPBJEC027881 | 0.81988032 | Plus | path:ko00940 | Phenylpropanoid biosynthesis | K00430 | E1.11.1.7; peroxidase; [EC:1.11.1.7] |
| evm.model.contig420.14 | SbSPL9 | GWHTBJEC023294 | GWHPBJEC022783 | evm.model.contig507.297 | GWHTBJEC028510 | GWHPBJEC027881 | 0.615927361 | Plus | path:ko00940 | Phenylpropanoid biosynthesis | K00430 | E1.11.1.7; peroxidase; [EC:1.11.1.7] |
| evm.model.contig106.94 | SbSPL12 | GWHTBJEC025733 | GWHPBJEC025164 | evm.model.contig507.297 | GWHTBJEC028510 | GWHPBJEC027881 | -0.612517328 | Minus | path:ko00940 | Phenylpropanoid biosynthesis | K00430 | E1.11.1.7; peroxidase; [EC:1.11.1.7] |
| evm.model.contig155.11 | SbSPL10 | GWHTBJEC024923 | GWHPBJEC024368 | evm.model.contig507.297 | GWHTBJEC028510 | GWHPBJEC027881 | 0.754795026 | Plus | path:ko00940 | Phenylpropanoid biosynthesis | K00430 | E1.11.1.7; peroxidase; [EC:1.11.1.7] |
| evm.model.contig455.712 | SbSPL14 | GWHTBJEC029854 | GWHPBJEC029206 | evm.model.contig507.297 | GWHTBJEC028510 | GWHPBJEC027881 | 0.593517012 | Plus | path:ko00940 | Phenylpropanoid biosynthesis | K00430 | E1.11.1.7; peroxidase; [EC:1.11.1.7] |
| evm.model.contig168.577 | SbSPL2 | GWHTBJEC001262 | GWHPBJEC001236 | evm.model.contig321.52 | GWHTBJEC007472 | GWHPBJEC007315 | 0.661143287 | Plus | path:ko00592 | alpha-Linolenic acid metabolism | K05894 | OPR; 12-oxophytodienoic acid reductase; [EC:1.3.1.42] |
| evm.model.contig273.92 | SbSPL4 | GWHTBJEC008542 | GWHPBJEC008370 | evm.model.contig321.52 | GWHTBJEC007472 | GWHPBJEC007315 | -0.500178184 | Minus | path:ko00592 | alpha-Linolenic acid metabolism | K05894 | OPR; 12-oxophytodienoic acid reductase; [EC:1.3.1.42] |
| evm.model.contig269.130 | SbSPL6 | GWHTBJEC015825 | GWHPBJEC015511 | evm.model.contig321.52 | GWHTBJEC007472 | GWHPBJEC007315 | 0.821400572 | Plus | path:ko00592 | alpha-Linolenic acid metabolism | K05894 | OPR; 12-oxophytodienoic acid reductase; [EC:1.3.1.42] |
| evm.model.contig155.257 | SbSPL11 | GWHTBJEC025167 | GWHPBJEC024607 | evm.model.contig321.52 | GWHTBJEC007472 | GWHPBJEC007315 | -0.621462772 | Minus | path:ko00592 | alpha-Linolenic acid metabolism | K05894 | OPR; 12-oxophytodienoic acid reductase; [EC:1.3.1.42] |
| evm.model.contig168.577 | SbSPL2 | GWHTBJEC001262 | GWHPBJEC001236 | evm.model.contig106.199 | GWHTBJEC025838 | GWHPBJEC025267 | 0.8182887 | Plus | path:ko00400 | Phenylalanine, tyrosine and tryptophan biosynthesis | K05359 | ADT,PDT;arogenate/prephenatedehydratase;[EC:4.2.1.914.2.1.51] |
| evm.model.contig273.92 | SbSPL4 | GWHTBJEC008542 | GWHPBJEC008370 | evm.model.contig106.199 | GWHTBJEC025838 | GWHPBJEC025267 | -0.58885581 | Minus | path:ko00400 | Phenylalanine, tyrosine and tryptophan biosynthesis | K05359 | ADT,PDT;arogenate/prephenatedehydratase;[EC:4.2.1.914.2.1.51] |
| evm.model.contig269.130 | SbSPL6 | GWHTBJEC015825 | GWHPBJEC015511 | evm.model.contig106.199 | GWHTBJEC025838 | GWHPBJEC025267 | 0.905694305 | Plus | path:ko00400 | Phenylalanine, tyrosine and tryptophan biosynthesis | K05359 | ADT,PDT;arogenate/prephenatedehydratase;[EC:4.2.1.914.2.1.51] |

| **Table S7 TPM expression of SPL target genes** | | | | | | |  | | | | |
| --- | --- | --- | --- | --- | --- | --- | --- | --- | --- | --- | --- |
| **Target gene Locus Id** | **Target gene Transcript Id** | **Target gene Protein Id** | **Root** | | | **Stem** | | | **Leaves** | | |
|  |  |  | **Root 1** | **Root 2** | **Root 3** | **Stem 1** | **Stem 2** | **Stem 3** | **Leaves 1** | **Leaves 2** | **Leaves 3** |
| evm.model.contig515.180 | GWHTBJEC004979 | GWHPBJEC004859 | 15.3771 | 18.7462 | 13.8044 | 89.3968 | 293.917 | 180.683 | 5.5491 | 2.68664 | 5.88329 |
| evm.model.contig522.188 | GWHTBJEC014099 | GWHPBJEC013833 | 58.2739 | 29.3481 | 60.4707 | 623.017 | 1165.33 | 832.447 | 64.8267 | 51.5167 | 86.6627 |
| evm.model.contig575.1 | GWHTBJEC011156 | GWHPBJEC010935 | 46.1502 | 213.525 | 140.017 | 21.4286 | 1.74844 | 8.85867 | 13.2537 | 2.52676 | 1.28866 |
| evm.model.contig300.287 | GWHTBJEC027402 | GWHPBJEC026792 | 18.4805 | 21.8383 | 47.4837 | 99.3211 | 87.6601 | 71.3816 | 13.0539 | 9.05878 | 13.48 |
| evm.model.contig507.297 | GWHTBJEC028510 | GWHPBJEC027881 | 35.8372 | 44.0927 | 46.2053 | 134.003 | 83.8119 | 259.904 | 3.74162 | 0.618941 | 0.248731 |
| evm.model.contig321.52 | GWHTBJEC007472 | GWHPBJEC007315 | 25.0904 | 23.9325 | 27.1928 | 224.286 | 182.935 | 266.672 | 107.374 | 45.2408 | 54.5701 |
| evm.model.contig106.199 | GWHTBJEC025838 | GWHPBJEC025267 | 7.93367 | 12.8821 | 8.34415 | 95.44 | 100.538 | 112.76 | 22.803 | 23.3232 | 7.62944 |
