# Supplementary figures and images for: Genome-wide identification, stress- and hormone-responsive expression characteristics, and regulatory pattern analysis of Scutellaria baicalensis SbSPLs
Source: Plant Mol Biol. 2024 Feb 16;114(2):20. doi: 10.1007/s11103-023-01410-z (PMC10873456; doi:10.1007/s11103-023-01410-z)

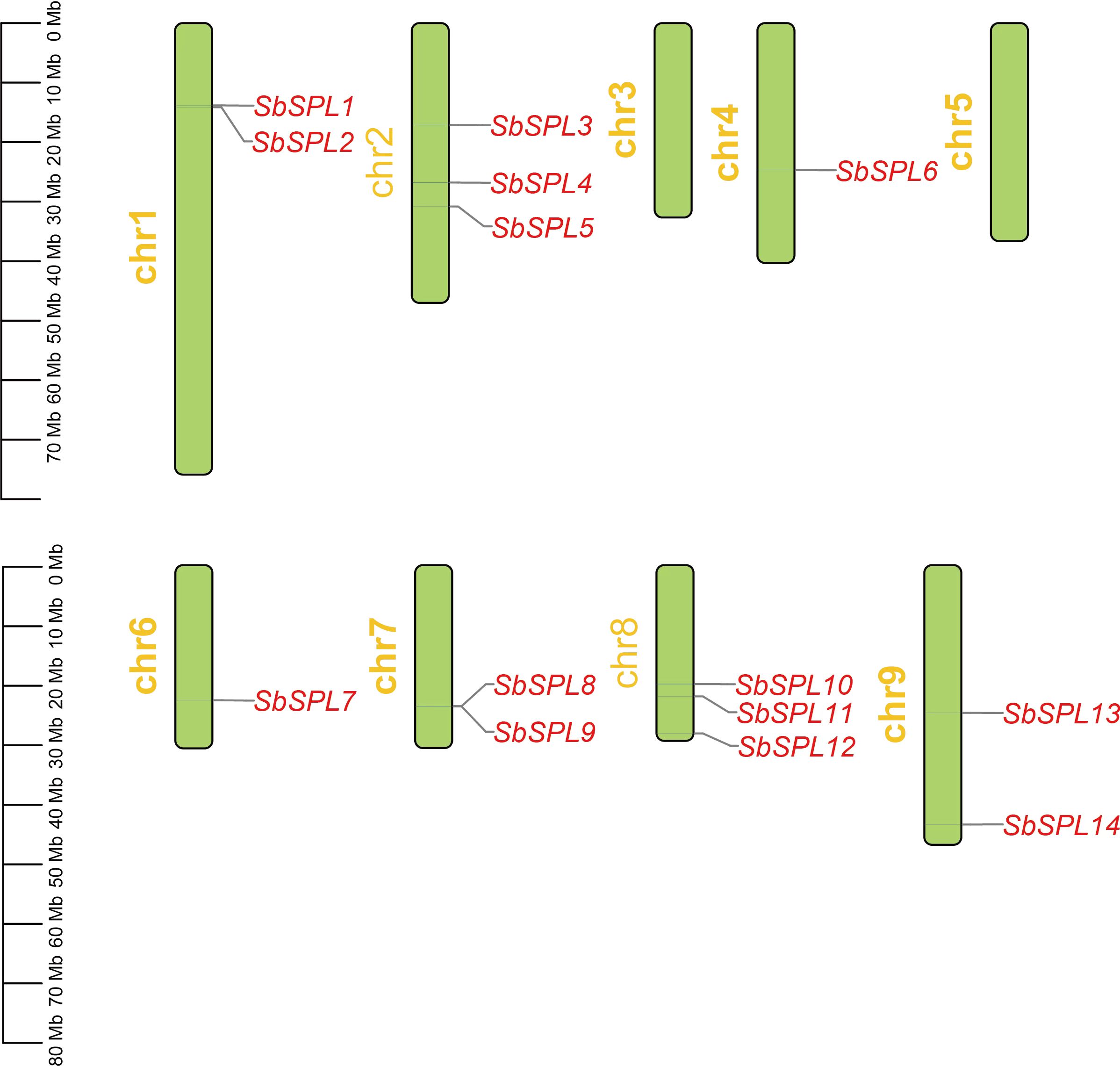

Supplement: Supplementary file 2 — Supplementary file2 (JPG 246 KB) [file 11103_2023_1410_MOESM2_ESM.jpg]

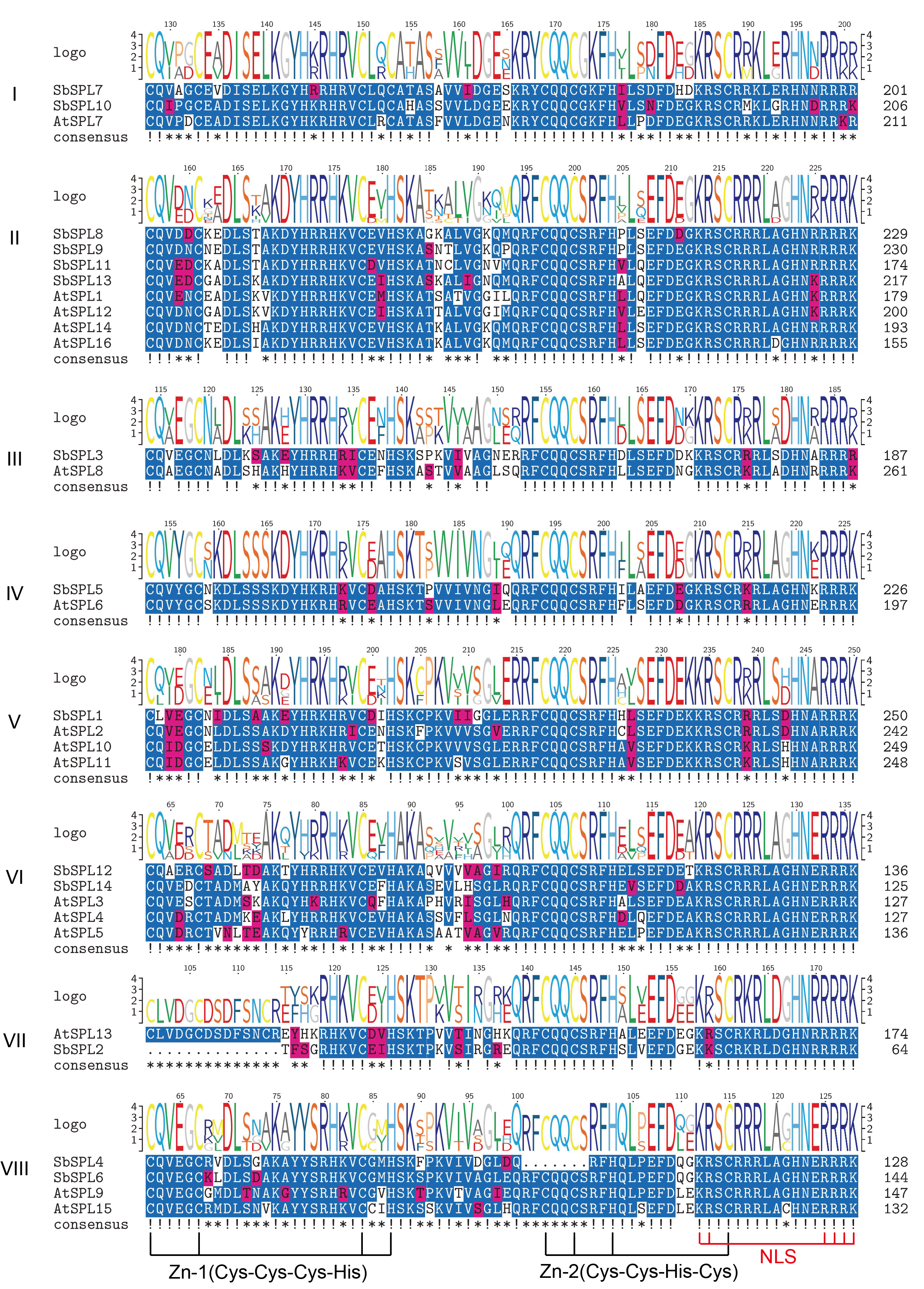

Supplement: Supplementary file 3 — Supplementary file3 (JPG 6287 KB) [file 11103_2023_1410_MOESM3_ESM.jpg]

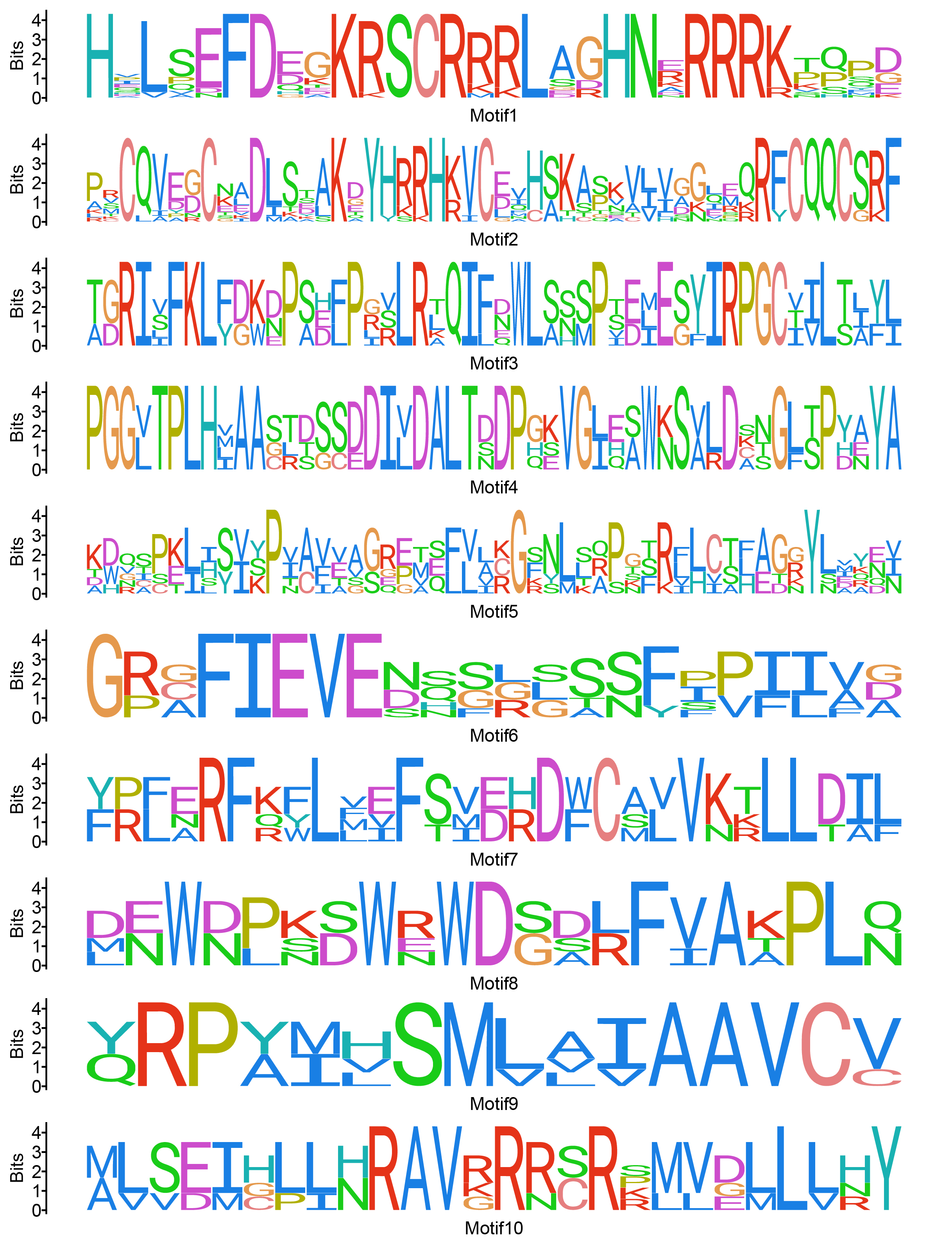

Supplement: Supplementary file 4 — Supplementary file4 (JPG 3054 KB) [file 11103_2023_1410_MOESM4_ESM.jpg]

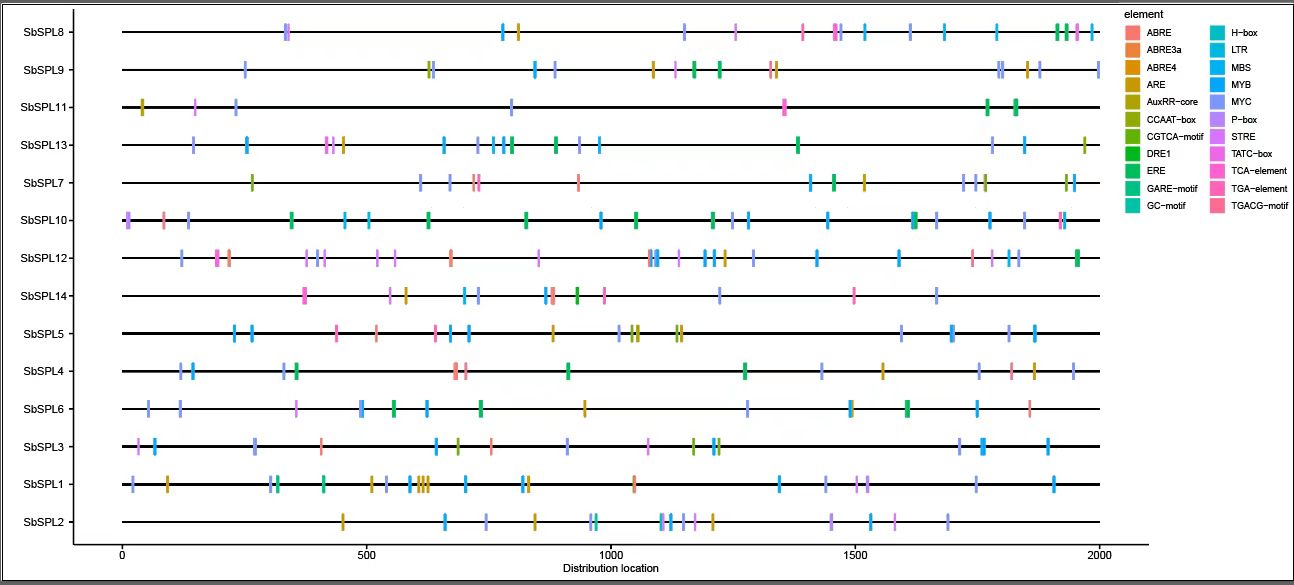

Supplement: Supplementary file 5 — Supplementary file5 (JPG 97 KB) [file 11103_2023_1410_MOESM5_ESM.jpg]

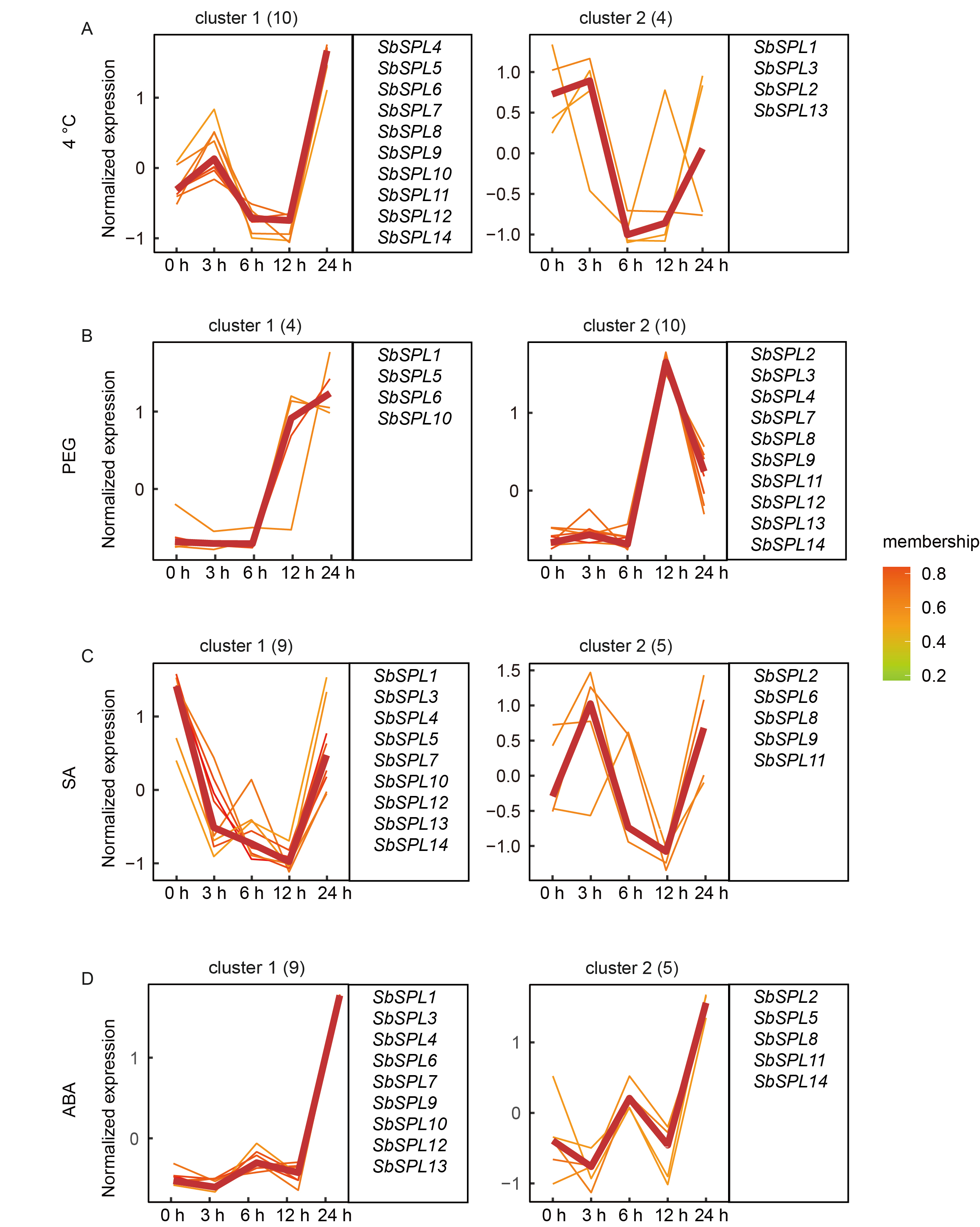

Supplement: Supplementary file 6 — Supplementary file6 (JPG 1484 KB) [file 11103_2023_1410_MOESM6_ESM.jpg]

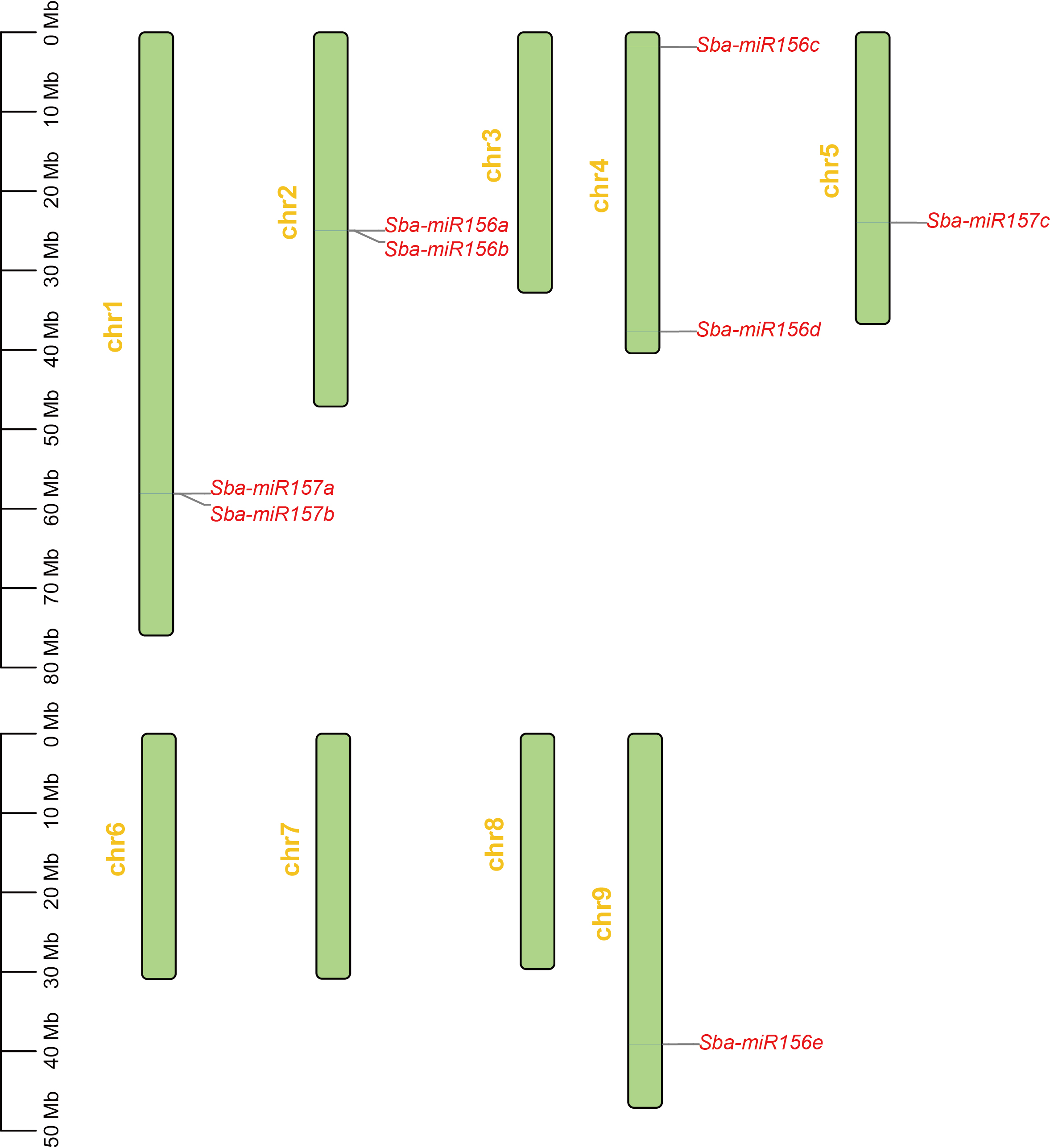

Supplement: Supplementary file 7 — Supplementary file7 (JPG 509 KB) [file 11103_2023_1410_MOESM7_ESM.jpg]

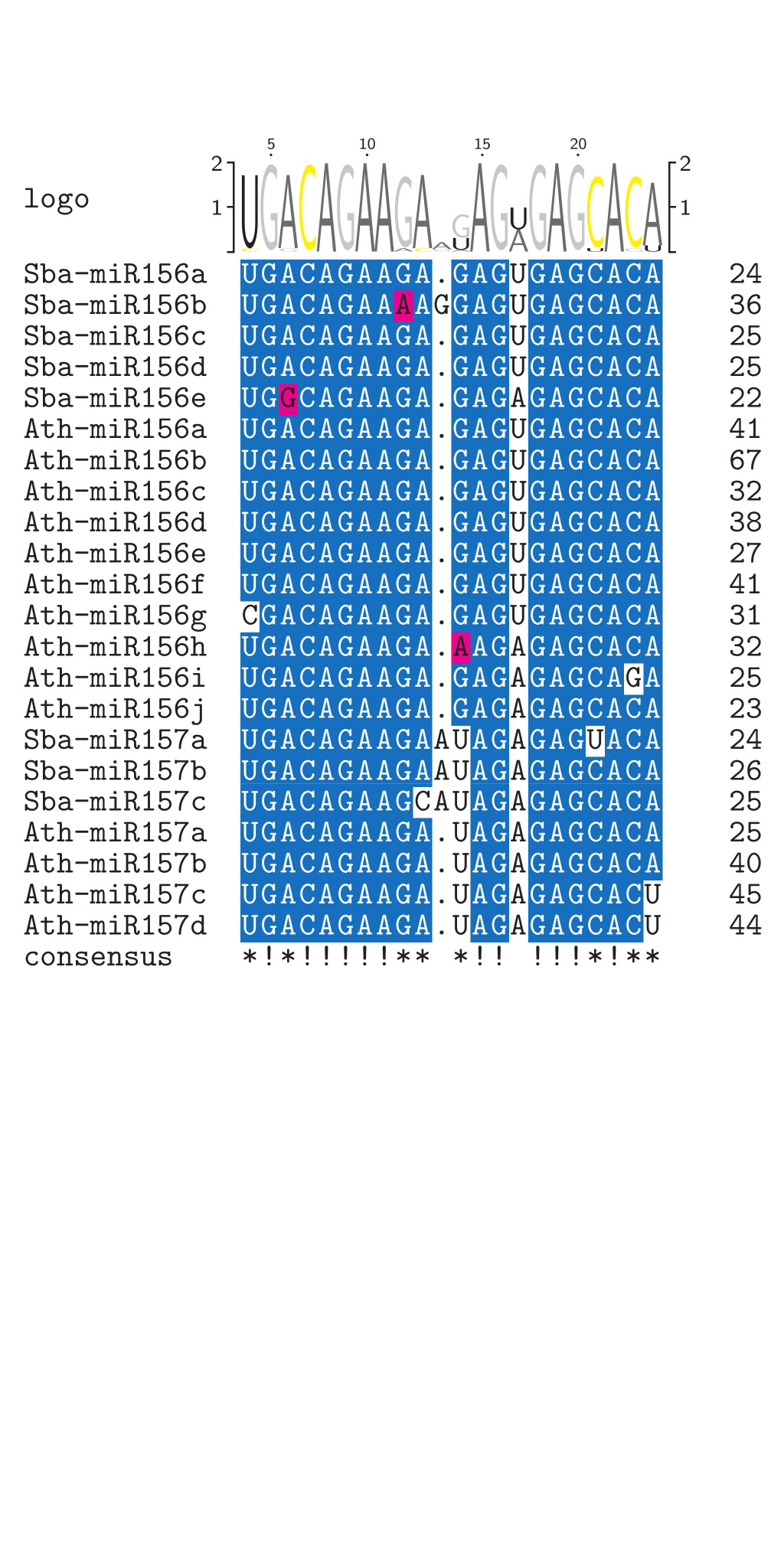

Supplement: Supplementary file 8 — Supplementary file8 (JPG 838 KB) [file 11103_2023_1410_MOESM8_ESM.jpg]

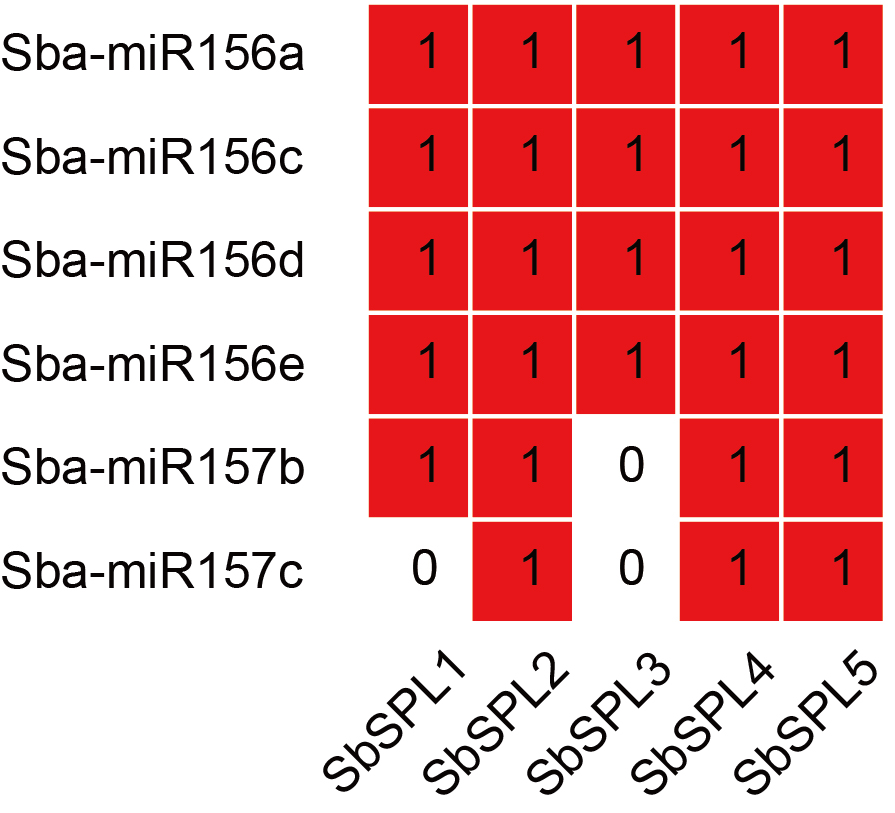

Supplement: Supplementary file 10 — Supplementary file10 (JPG 225 KB) [file 11103_2023_1410_MOESM10_ESM.jpg]
